# Development of the Parental Experience with Care for Children with Serious Illnesses (PRECIOUS) quality of care measure

**DOI:** 10.1186/s12904-024-01401-x

**Published:** 2024-03-08

**Authors:** Felicia Jia Ler Ang, Mihir Gandhi, Truls Ostbye, Chetna Malhotra, Rahul Malhotra, Poh Heng Chong, Zubair Amin, Cristelle Chu-Tian Chow, Teresa Shu Zhen Tan, Komal Tewani, Eric Andrew Finkelstein

**Affiliations:** 1 Lien Centre for Palliative Care, Duke-NUS Medical School, 8 College Road, Singapore, 169857 Singapore; 2 Programme in Health Services & Systems Research, Duke-NUS Medical School, Singapore, Singapore; 3 Biostatistics, Singapore Clinical Research Institute, Singapore, Singapore; 4 Tampere Center for Child, Adolescent, and Maternal Health Research: Global Health Group, Tampere University, Tampere, Finland; 5 Duke Global Health Institute, Duke University, Durham, USA; 6 Centre for Ageing Research and Education, Duke-NUS Medical School, Singapore, Singapore; 7 HCA Hospice Limited, Singapore, Singapore; 8 Department of Neonatology, Khoo Tech Puat-National University Children’s Medical Institute, National University Hospital, Singapore, Singapore; 9 Department of Paediatrics, Yong Loo Lin School of Medicine, National University of Singapore, Singapore, Singapore; 10 Children’s Complex and Home Care Services, KK Women’s & Children’s Hospital, Singapore, Singapore; 11 Department of Paediatrics, Khoo Tech Puat-National University Children’s Medical Institute, National University Hospital, Singapore, Singapore; 12 Department of Gynaecological Oncology, KK Women’s & Children’s Hospital, Singapore, Singapore; 13 Duke Department of Population Health Sciences, Duke University, Durham, USA

**Keywords:** Process assessment, Quality of care, Parent, Process measure, Person-centered care

## Abstract

**Background:**

Parent-reported experience measures are part of pediatric Quality of Care (QoC) assessments. However, existing measures were not developed for use across multiple healthcare settings or throughout the illness trajectory of seriously ill children. Formative work involving in-depth interviews with parents of children with serious illnesses generated 66 draft items describing key QoC processes. Our present aim is to develop a comprehensive parent-reported experience measure of QoC for children with serious illnesses and evaluate its content validity and feasibility.

**Methods:**

For evaluating content validity, we conducted a three-round Delphi expert panel review with 24 multi-disciplinary experts. Next, we pre-tested the items and instructions with 12 parents via cognitive interviews to refine clarity and understandability. Finally, we pilot-tested the full measure with 30 parents using self-administered online surveys to finalize the structure and content.

**Results:**

The Delphi expert panel review reached consensus on 68 items. Pre-testing with parents of seriously ill children led to consolidation of some items. Pilot-testing supported feasibility of the measure, resulting in a comprehensive measure comprising 56 process assessment items, categorized under ten subthemes and four themes: (1) *Professional qualities of healthcare workers*, (2) *Supporting parent-caregivers*, (3) *Collaborative and holistic care*, and (4) *Efficient healthcare structures and standards*. We named this measure the *PaRental Experience with care for Children with serIOUS illnesses (PRECIOUS).*

**Conclusions:**

PRECIOUS is the first comprehensive measure and has the potential to standardize assessment of QoC for seriously ill children from parental perspectives. PRECIOUS allows for QoC process evaluation across contexts (such as geographic location or care setting), different healthcare workers, and over the illness trajectory for children suffering from a range of serious illnesses.

**Supplementary Information:**

The online version contains supplementary material available at 10.1186/s12904-024-01401-x.

## Background

Each year, over 21 million children globally suffer from serious life-threatening and life-limiting illnesses [1, 2], placing significant burden of care on their parents and the health system. Parents of these children often need to navigate convoluted healthcare systems to make crucial medical and financial decisions [3]. As a result, they too are susceptible to adverse health and psychosocial outcomes [4]. Given the established relationship between parental and child well-being [5, 6], healthcare workers (HCWs) should strive to meet the needs of both groups and approach the parent/child dyad as a single unit of care [7–9].

According to the Donabedian Quality of Care (QoC) model [10], process measures evaluate the activities that revolve around care delivery, such as communication between patients and HCWs and timely notifications of clinical or lab test results. Parent-Reported Experience Measures (PaREMs) are process measures that help us to understand “what” and “how” care activities occurred from the perspective of parents [11, 12], acknowledging them not only as proxies of the child-patient but also as the other key recipient of care services [13–15]. PaREMs are invaluable for reporting and driving process improvements in, within, and across health and social care settings, including homes, hospices, and clinics [16, 17]. They support parental engagement, which can in turn educate both parents and HCWs, inform policymakers, and improve service delivery and governance [18].

Most existing validated experience measures are designed for adults with serious illnesses. Moreover, other measures intended for children, such as the widely used Consumer Assessment of Healthcare Providers and Systems surveys, of which only a couple have child versions, were not designed for parents of seriously ill children with unique care needs. Rather, most measures are for children who are not necessarily seriously ill or are otherwise healthy but hospitalized episodically. Our published scoping review, which mapped and evaluated PaREMs for parents of seriously ill children, showed that there is no single PaREM that is applicable across the illness trajectory and diverse service providers involved in a seriously ill child’s care network [19]. For example, while the Measure of Processes of Care (MPOC) addresses family-centred behaviors of HCWs from community-based centres, it is not intended for acute or hospital care. Conversely, the Quality of Children’s Palliative Care Instrument (QCPCI) assesses specialist hospital care throughout the illness, but excludes community-based services. The Pediatric Integrated Care Survey [20] captures parental experiences of care coordination across settings, but omits some QoC domains crucial for the chronic, serious illness journey such as parental empowerment, caregiving stress reduction, and access to financial and informational resources – which are vital for parents managing prolonged child illnesses with high caregiving demands.

Given the complex and multidisciplinary nature of pediatric serious illnesses, there is a need for a comprehensive PaREM that can assess quality of care across diverse settings and HCWs for seriously ill children and their parents. Such measures should also strive to be widely relevant both across contexts (such as geographic location or care setting) and throughout the illness trajectory for children suffering from a range of serious illnesses. Involving a series of steps, we first started with a qualitative study to identify key care processes which are important to parents of seriously ill children yet relevant across various care settings and their HCWs, and over the illness trajectory [21]. Through this formative work, we drafted 66 preliminary QoC items categorized into four themes and 10 subthemes (Table 1). Each care process described parental priorities regarding care practices, interactions, services and procedures throughout the illness trajectory and across HCWs. Presently, to address limitations in existing PaREMs, we aim to *establish content validity*, *assess feasibility* (through survey response rates and time) and provide *preliminary evidence of construct validity* for a newly developed measure which standardizes QoC evaluation from the parental perspective across the care continuum and over time.

**Table 1:** Themes, subthemes, and number of quality of care items resulting from the inductive qualitative study which were tested in the modified Delphi expert panel review

Theme	Subtheme	Number of items
1. Professional qualities of healthcare workers	1.1 Responsive and sensitive communication	11
	1.2 Competency of healthcare delivery	5
2. Supporting parent-caregivers	2.1 Empowering parent-caregivers	7
	2.2 Providing psychosocial support to parents and family	6
	2.3 Reducing caregiving stress and burdens	5
3. Collaborative and holistic care	3.1 Shared decision-making	8
	3.2 Holistic approach to care for the child	6
4. Efficient healthcare structures and standards	4.1 Accessible medical care	6
	4.2 Effective administration and facilities	6
	4.3 Coordination and continuity of care	6
Total number of items		66

## Methods

The study was conducted in Singapore, a multi-cultural and multi-ethnic country in Southeast Asia. Singapore’s healthcare system is a mixed financing model that blends individual responsibility through compulsory savings (‘Medisave’) and government subsidies with national insurance (‘Medishield’) for comprehensive coverage [22]. We first revised the initial 66 QoC items to present them from a first-person perspective and added standardized stems (e.g., Over the past 12 months, our child's healthcare workers…), response options (i.e., Always to Never) and real-world examples to clarify complex terminologies (e.g., …advised us on how to reduce our child's medical expenses, such as access to subsidies or financing schemes). We then adopted a multi-method, multi-stakeholder approach, incorporating modified Delphi expert panel review, establishment of a steering committee, and pre-testing and pilot-testing with parents.

### Delphi expert panel review

The Delphi technique is a structured group communication and consensus building process, which iteratively engages experts to evaluate complex real-world issues [23, 24]. This established and effective method gathers and synthesizes informed opinions on focused areas of interest [25]. The approach, widely used to develop guidelines, criteria, quality indicators, and policy frameworks, is grounded in expert opinions and lived experiences [26, 27]. We used a modified online Delphi approach to determine the content validity of the drafted items [28], which has been similarly used to determine the content validity of items in adult patient-reported experience measures [29], quality appraisal tools [30], and surveys measuring evidence-based practice [31]. This technique was particularly helpful for us to aggregate existing expertise and the item set developed from earlier formative work.

#### Delphi expert panel review: participants and procedures

We conducted a three-round modified online Delphi expert panel review from April to June 2022, moderated by three facilitators (EAF, CM, FAJL) [32]. While the classic Delphi methodology employs four rounds [33], we employed three rounds because others have found that this enables adequate reflection on group responses and is appropriate to reach consensus [34]. We defined an “expert” as an individual who has over one year of experience working directly with children suffering from serious illnesses and their families, either directly providing care services to them or conducting research related to them. We purposively sampled local experts from multiple disciplines across the major institutions caring for children with serious illnesses. This purposive sampling technique ensured that the content would be relevant to the target parent population and aligned with the local sociocultural and healthcare system. In line with our aim to develop a widely applicable PaREM, we also included international experts with experience in designing parent-reported experience measures in other settings. We invited each expert via email to seek their consent. The panel included HCWs from multiple disciplines and researchers working with children with serious illnesses or pediatric measure development. In addition, in the spirit of family-centered care, we included parent-caregivers as experts as they can provide the most authentic and first-hand experience as end-users. We provided each expert with a handout (Additional file 1) explaining the study’s purpose, procedures, and materials before the review.

During each two-week round, experts independently provided input which the facilitators used to make modifications to the measure during one-week breaks between rounds. We hosted all survey links on Qualtrics (NUS Enterprise). In Round One, experts voted for each item on a three-point Likert scale in response to the question “Does the item appropriately capture the subtheme…”. The response options were: (i) ‘No, not appropriate’; (ii) ‘Yes, with changes to item or response options’ with accompanying free-text input; and (iii) ‘Yes, no changes to recommend’. After voting on items within each subtheme individually, we presented experts with all corresponding items under that theme and asked them if any key processes were missing from a QoC perspective. If they responded ‘Yes’, they were prompted to suggest additional processes using a free-text box.

In Round Two, experts continued to vote on each item in response to the same question. We included a free-text response box with every item to enable experts to elaborate on their responses and offer open-ended input. Thus, the response options were: (i) No, not appropriate; and (ii) Yes, appropriate.

In Round Three, we presented experts with a full list of all items, subthemes and themes and requested that they suggest further improvements to any aspect, such as word choice, redundancies, order of items, etc. To complete the Delphi process, experts had to respond to all three rounds, otherwise we marked their participation as incomplete.

**Fig. 1:**
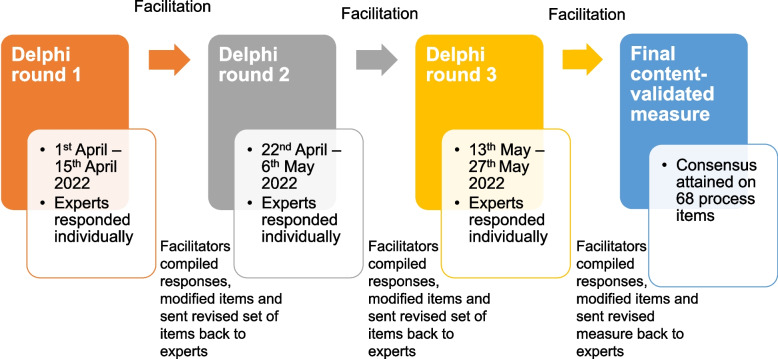
Summary of procedures in the Delphi expert panel review

As outlined in Fig. 1, feedback from each round informed subsequent modifications to the items, exemplifying the Delphi method’s iterative, consensus-building, and expert-driven nature. Items in each round were modified and added from previous rounds, allowing for the items’ iterative evolution. Experts also had access to prior rounds’ results, facilitating reflection and allowing possible adjustment of their own views. Results and expert feedback for each item per round were shared anonymously with all experts to prevent bias and protect participants from potential negative perceptions of their views.

#### Delphi expert panel review analysis

For every round, we compiled aggregated ratings, whether consensus was achieved, and made modifications to previous versions of the measure. We predefined consensus as 70%, as commonly suggested and used in Delphi studies [35], to represent a substantial majority without being impractical. This consensus threshold represented the proportion of experts who responded either ‘Yes, with changes’ or ‘Yes, no changes to recommend’ in the first round, and who responded ‘Yes, appropriate’ in the second round. This aimed to achieve expert agreement that each item appropriately represented each subtheme’s targeted aspect of quality of care (the ‘construct’). Drafted items which had attained consensus were termed “candidate” items and retained for the next phase of pre-testing.

### Establishment of steering committee

After establishing content validity of the candidate items through the modified Delphi process, we formed a multidisciplinary steering committee to guide the development of the full measure. This committee, consisting of experts in measure development (MG), health services research (TO, RM) and palliative care research (EAF, CM), met regularly to refine the measure. Additionally, an experienced former nurse provided input on early drafts, considering local sociocultural and linguistic nuances.

### Pretesting

From July to September 2022, we pretested the measure using cognitive debriefing interviews with parents of seriously ill children [36] to identify potentially imprecise wording, poor ordering of items, and mismatches between intended and interpreted meanings of items [37]. We incrementally evaluated and improved the relevance of the candidate items and instructions. To test whether the QoC processes remained relevant to parents of children at various developmental stages, we staggered the interviews across four batches. Each batch corresponded to children’s age group (early childhood (0 – 5 years), middle childhood (6 – 12 years), adolescence (13 – 17 years), and young adulthood (18 – <21 years)). After each batch of interviews, interim steering committee meetings were held for iterative review and modification of items and instructions.

For pre-testing we targeted a sample size of 12 participants, aiming for three parents per child’s age group. This sample size was selected to provide a 70% chance of detecting any omission that 10% of the population would deem significant, calculated based on the complement rule in probability theory, expressed as (1 – 0.1)^12^ = 0.28 [38]. Thus, with 12 participants, we had a 72% likelihood of identifying important omissions noted by 10% of parents.

#### Pretesting participants and procedures

We recruited adult (>21 years old) parents of seriously ill children (<21 years old) in Singapore through referrals from study collaborators. Parents of young adults (18 to < 21 years) were included as the majority of individuals with developmental disabilities continue to be cared for in their family homes [39]. We conducted face-to-face interviews in parents’ homes or through videoconference based on their preference, which were audio-recorded and transcribed. All interviews were held by the first author, a female health services researcher with experience in qualitative methods who had no prior or dependent relationship with any parent. We used a think-aloud approach during interviews where parents verbalized their thoughts while completing the measure [40]. This allowed us to evaluate the meaning of parent’s answers, the degree of difficulty encountered in completing the measure, and the nature of completion problems. We also employed a concurrent ‘verbal-probe’ method [41], where we asked parents specific questions after each item based on Tourangeau’s four-stage cognitive model of question response [42] to determine how information is understood, retrieved, judged, and reported. An illustration of the verbal probes used in the cognitive interviews is shown in Table 2. After completing the measure, we individually debriefed parents about their general impressions of its length and overall ease, asked them to highlight any difficulty in understanding the items, and explain whether the care processes were relevant to them.

**Table 2:** Verbal probes used in cognitive interviews based on Tourangeau’s four-stage cognitive model of question response

Cognitive or measure component	Verbal probe	Response errors or issues
**Follow up on observations**	Why did you pause on this? What is going through your mind?	
**1) Comprehension**	What does [content or term] mean to you?	Unknown terms, ambiguous concepts, long and overly complex
**2) Retrieval/ recall of information**	What did you remember when you read this?	Recall difficulty
**3) Judgement/estimation**	Describe your experiences with [concept] over the (timeframe)	Biased or sensitive, estimation difficulty
**4) Response Mapping/reporting**	How did you select your [response option?]	Incomplete response options
**Overall feedback**	Are there things that we do not ask about your child’s care that you think are important? What do you think about the length of the measure? Was it difficult for you to complete the measure?	Relevance, length, participant fatigue

#### Pretesting analysis

We conducted cognitive interviews and steering committee discussions iteratively across the four batches. After each cognitive interview, we summarized responses to items and verbal probes from the think-aloud approach. We identified items where parents had difficulty understanding terms or phrasing and highlighted those where they had interpreted differently than intended. Verbal probes helped us to compare parents’ understanding of items to the intended meaning. We also noted items where a high proportion (>25%) of parents selected the ‘Not Applicable’ response option, indicating a lack of clarity or relevance to parental experiences.

### Pilot-testing

After successfully completing pretesting, we conducted pilot-testing [37] from October to December 2022 to identify potential issues in administering the measure during full-scale validation and to generate preliminary data on construct validity and measurement properties in the target population.

#### Pilot-testing participants and procedures

We recruited 30 parents (>21 years old) of seriously ill children (<18 years old) in Singapore, following recommended sample size for pilot-studies in measure development [43]. We recruited a diverse representation of parents through partner organizations, parent-advocates, and co-investigators. Parents were invited to self-administer an online survey hosted on Qualtrics (NUS Enterprise), which included sociodemographic questions and three measures: our new PaREM and two measures previously identified in the scoping review—the MPOC-20 [44] and QCPCI [45]. MPOC-20 measures parents’ perceptions of family-centeredness of community services and QCPCI evaluates hospital-based palliative care for children with life-threatening conditions. Both are QoC measures targeting different care settings but applicable to both short- and long-term care. Thus, MPOC-20 and QCPCI were included since they have some overlapping constructs with our PaREM and were deemed to be useful for evaluating the measurement properties of the new measure in full-scale validation. Higher scores denote better QoC in all measures. We randomized the order of administration of the three measures to mitigate order effects.

#### Pilot-testing analysis

We followed standard scoring, reporting and interpretation protocols for MPOC-20 and QCPCI scores according to CanChild’s and Widger et al.’s guidelines, respectively. PRECIOUS utilized a 5-point Likert scale ranging from Never = 0 to Always = 4, with a “Not Applicable” response option for context-dependent processes. After scoring the measures, we conducted a preliminary assessment of the construct validity of the PRECIOUS subscales by descriptively comparing subscale scores across the three PaREMs. For each PRECIOUS item, we calculated the number of valid responses, percentage of parents choosing ‘Never’ (floor), 25^th^ percentile, mean (SD), 50^th^ percentile, 75^th^ percentile, percentage of parents choosing ‘Always’ (ceiling), and minimum and maximum scores. We also tracked time to complete the entire survey and response rates: 1) proportion eligible, 2) proportion consenting, 3) dropout rate, and 4) data completeness (item-response). To assess convergent validity, we calculated Spearman’s correlation coefficient (ρ) between PRECIOUS, QCPCI and MPOC-20 subscales, and an overall Quality of Care rating from QCPCI. MPOC-20 did not include a global QoC rating.

## Results

### Delphi expert panel review results

While 33 experts were invited and 26 consented to participate, 24 experts completed the Delphi rounds (2 incompletes). Their experience covered various areas: hospice care (*n* = 1), pediatric palliative care (*n* = 2), pediatric complex care (*n* = 1), intensive or critical care (*n* = 2), measure development (*n* = 2), allied health (*n* = 4), pediatric nursing (*n* = 4), home care (*n* = 2), health services research (*n* = 2), and parent-caregiving (*n* = 4). Experts had diverse experience levels, with 8 having over 20 years of experience, 6 having 10-19 years, 4 having 5-9 years, and 6 having 1-4 years of experience. 83% (*N* = 20) of experts were from Singapore and 17% (*N* = 4) were international experts from Canada (*N* = 2), the United Kingdom (*N* = 1), and the United States of America (*N* = 1).

Detailed results specifying all aggregated ratings, consensus and modifications across rounds one and two are presented in Additional file 2. In summary, after Round One, we eliminated one item due to lack of consensus: “I have the ability to choose my child's healthcare workers.” We attained consensus on 65 items and modified 49 items. For instance, we reworded the original item “I receive the same information from different healthcare workers" to "I receive consistent information from different healthcare workers,” based on feedback that HCWs should avoid providing conflicting advice to parents but do not need to provide identical information. Experts proposed 14 new processes for panel review in Round Two that were not covered by existing items, including “…communicate with my child in a way that is sensitive to his/her needs”.

After Round Two, we eliminated 7 items due to lack of consensus or high degree of overlap with other items, including “… make sure my child's medical equipment are properly maintained beyond healthcare facilities”. We attained consensus on 72 items, with 41 being modified.

**Fig. 2:**
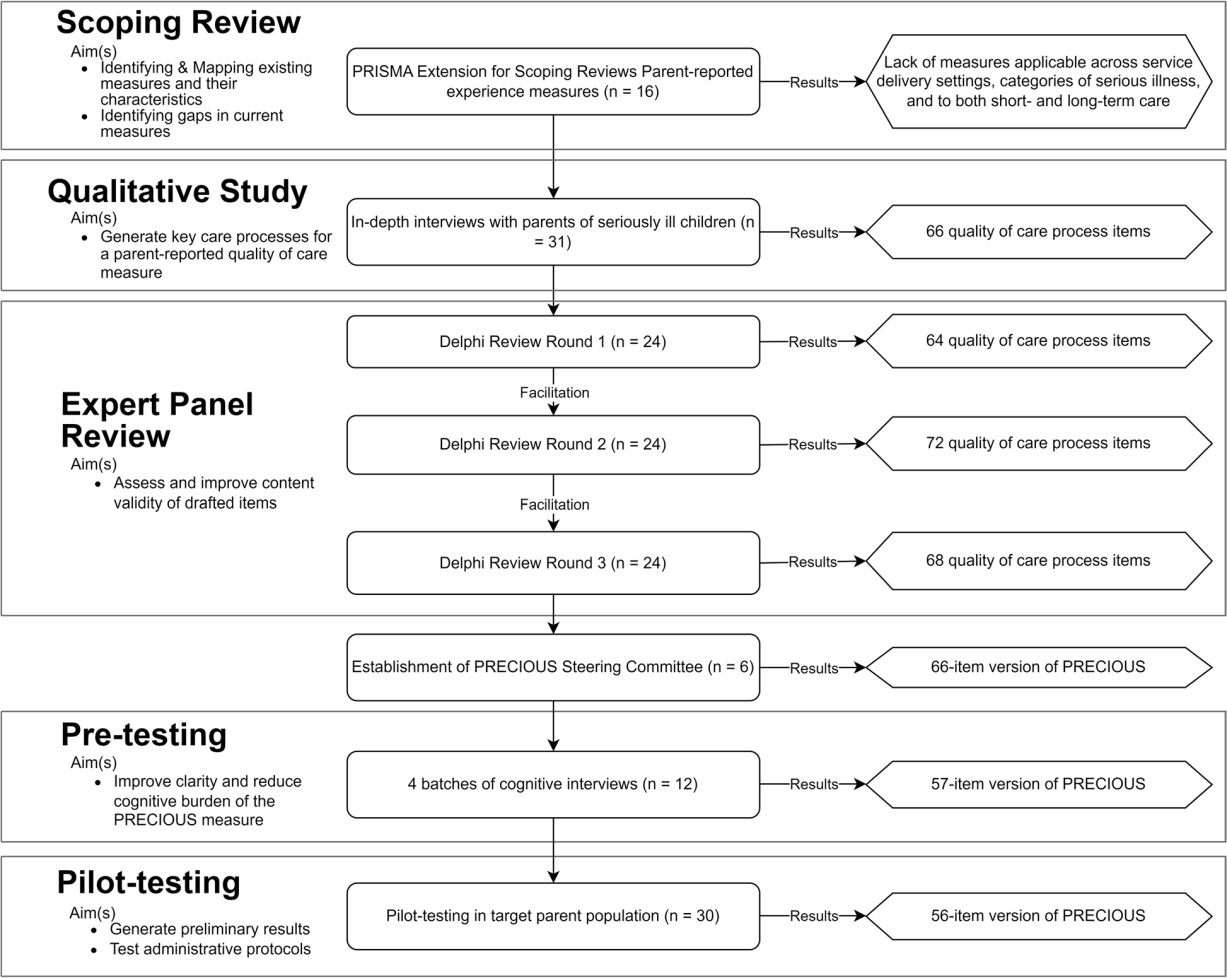
Flowchart summarizing the various aims, procedures and results of various phases of measure development

After Round Three, we consolidated and incorporated expert recommendations, and the facilitators conducted a review to combine overlapping items. For example, “… ensure I am fully informed” and “…give me information on my child's condition in a timely manner” were merged into one item. We thus removed 4 items and modified 20, while 48 remained unchanged. For example, we clarified the wording of the item “… give me all available management options for my child” to “…inform me of all available medical options for my child.” No new items were proposed during Round Three. Overall, consensus was achieved on 68 candidate items under four themes and 10 subthemes at the end of the Delphi review. Figure 2 presents a flowchart summarizing the aims, procedures and results of various phases of the measure development process.

#### Steering committee actions following Delphi expert panel review

After reviewing the 68 candidate items, the steering committee suggested removing two items that were conceptually indistinguishable, standardized item response options, and refined the instructions. These resulted in the full measure with 66 items for pre-testing. We named the measure the *PaRental Experience with care for Children with serIOUS illnesses* (PRECIOUS).

### Pretesting results

We conducted cognitive interviews with 12 parents of seriously ill children (characteristics in Table 3). Additional file 3 contains details of pre-testing feedback and modifications across cognitive interviews. Parents of children in Early childhood, Middle childhood and Adolescence interpreted the items similarly and reported that existing processes were comprehensive and relevant. However, parents of Young Adults felt that their experience with, and hence the relevance of, PRECIOUS items changed substantially after their child left the purview of the pediatric health system.

**Table 3:** Characteristics of participating parents (*n* = 42) and children in pre-testing (*n* = 12) and pilot-testing (*n* = 30)

Parent characteristics (number, %)		Child characteristics (number, %)	
**Men**	14 (33)	**Boy**	28 (67)
**Mean age, years (range)**	42.6 (31—52)	**Mean age, years (range)**	9.3 (2.2 – 19.5)
**Highest education level**		**Age range**	
Post-secondary	38 (90)	0 – 5 (Early Childhood)	18 (43)
Secondary school or Vocational Training	4 (10)	6 – 12 (Middle Childhood)	13 (31)
**Mean number hours spent on caregiving per week (Range)**	93.6 (14—168)	13 – 17 (Adolescence)	7 (17)
**Married**	42 (100)	18 – < 21 (Young Adult)	4 (10)
**Religion**		**Category of diagnosed conditions**^**a**^	
Christianity	8 (19)	Category 1	7 (17)
Buddhism	10 (24)	Category 2	6 (14)
Catholic	11 (26)	Category 3	12 (29)
Free thinker	6 (14)	Category 4	17 (40)
Taoism	2 (5)		
Islam	5 (12)		
**Caregiving roles (Answered ‘Yes’)**			
Physically provide care to child (e.g., help with day-to-day activities)	37 (88)		
Ensure provision of care (e.g., supervise helper to look after child)	37 (88)		
Make decisions about treatments the child receives	41 (98)		
Pay for the medical and health care expenses	37 (88)		
**Employment**			
Stopped working to take care of child	9 (21)		
Full-time job	25 (60)		
Homemaker	4 (9.5)		
Part-time job	4 (9.5)		

^a^Categorization as defined by Together for Short Lives, United Kingdom: Category 1. Life-threatening conditions for which curative treatment may be feasible but can fail; Category 2. Conditions where premature death is inevitable; Category 3. Progressive conditions without curative treatment options; Category 4. Irreversible but non-progressive conditions causing severe disability, leading to susceptibility to health

#### Steering committee actions following pre-testing

Given that parents of Young Adults transitioning from the pediatric to adult health system reported that PRECIOUS items were less relevant to them, the steering committee decided to limit the measure to children under 18 years old in subsequent phases. Additionally, we took extra care to ensure clarity of the statements without modifying the intended construct. For example, the item “…discussed with us how the scope of care could be tailored to provide comfort for my child” was clarified to “…discussed with us how care could be adjusted to improve my child’s comfort”.

Broadly, the modifications made to the items throughout the iterative process of pre-testing reduced the number of items, simplified the terms and sentences used, and reduced the number of examples. For example, parents found the item “…gave us opportunities to advocate or speak up for my child” to be cumbersome and this was streamlined to “…listened to us when we spoke up for my child”. Pre-testing concluded with most items being modified and 9 being removed; *57 items* were retained in the measure (abbreviated items in Table 4, full measure in Additional file 4).

**Table 4:** Abbreviated items of the 57-item content-validated PaRental Experience with care for Children with serIOUS illnesses (PRECIOUS)

Item code	Theme^a^	Subtheme^b^	Indicator
**p1**	ESS	Amc	Access to range of medical expertise needed to manage child's condition(s)
**p2**	ESS	Amc	Access to sufficient financial support for child’s medical expenses so costs did not stop child from receiving recommended medical care
**p3**	ESS	Ccc	Care worker/team that organized child's care across different care services
**p4**	ESS	Ccc	Consistent information from different healthcare workers
**p5**	SPC	Rb	Access to sufficient financial support for child's non-medical expenses so costs did not stop child from receiving recommended non-medical care
**p6**	CHC	Hac	Appropriate allied health support to meet parental goals for child's development
**p7**	CHC	Hac	Child formally received advice or care from palliative or supportive care team or specialist(s)
**p8**	CHC	Hac	**[DISPLAYED IF RESPONSE = ‘YES’ to p7]** Parent introduced to palliative or supportive care team or specialist(s) at appropriate time
**Over the past 12 months, our child’s HCW…**			
**p9**	ESS	Amc	Advised parent on how to obtain medical equipment(s) and supplies
**p10**	ESS	Ccc	Worked together to ensure medical condition(s) are well managed
**p11**	ESS	Amc	Approachable when parent needed advice
**p12**	ESS	Ccc	Worked together towards common goals for child
**p13**	ESS	Ccc	Organized appointments to reduce hospital visits
**p14**	PQ	Rsc	Built a trusting relationship with parents
**p15**	PQ	Rsc	Kept parents well informed about child’s condition
**p16**	PQ	Rsc	Communicated in sensitive way
**p17**	PQ	Rsc	Gave enough time for parent think about decisions
**p18**	PQ	Pcom	Responsive in managing medical issues
**p19**	PQ	Pcom	Avoided treatments and investigations not aligned with parental goals
**p20**	PQ	Pcom	Managed physical symptoms to make sure child was comfortable
**p21**	PQ	Pcom	Ensured child's wellbeing when child was under their care
**p22**	SPC	Emp	Kept parents updated about symptoms of clinical deterioration
**p23**	SPC	Emp	Equipped parents with skills to confidently care for child
**p24**	SPC	Emp	Acknowledged parental efforts
**p25**	SPC	Emp	Listened to parents when they advocated for child
**p26**	SPC	Pss	Showed care and concern
**p27**	SPC	Pss	Helped parents maintain hope
**p28**	SPC	Pss	Prepared parents for what may lie ahead
**p29**	SPC	Pss	Provided a kind listening ear
**p30**	SPC	Rb	Advised parents on how to reduce medical expenses, such as access to subsidies or financing schemes
**p31**	CHC	Hac	Interacted well with child
**p32**	CHC	Hac	Assessed child's physical, cognitive and emotional development
**p33**	CHC	Sdm	Informed parents of available medical options
**p34**	CHC	Sdm	Clearly explained advantages and disadvantages of options for parents to make informed decisions
**p35**	CHC	Sdm	Discussed how care could be adjusted to improve child's comfort
**p36**	CHC	Sdm	Involved parent as much as they wanted in decision-making
**p37**	CHC	Sdm	Considered parental preferences for treatments
**p38**	SPC	Emp	Invited parents to contribute to the community
**p39**	CHC	Hac	Treated child in a kind and respectful way
**p40**	PQ	Rsc	Respectful of spiritual or religious beliefs and practices
**p41**	SPC	Pss	Helped parents access available parent support groups
**p42**	SPC	Rb	Offered information on specialized transport for child
**p43**	SPC	Pss	Supported family's emotional needs related to child's condition
**p44**	SPC	Rb	Found someone to take care of child when parents needed help
**p45**	CHC	Hac	Provided emotional support to child
**p46**	CHC	Hac	Helped child access special needs school/day-care
**p47**	CHC	Hac	Communicated child's medical needs in school/day-care to their staff
**p48**	ESS	Ccc	Ensured smooth transition of care for child across different care settings
**p49**	ESS	Eaf	Attended to child within reasonable amount of time at Emergency Department
**Did your child spend at least 1 night in a hospital in the last 12 months? [DISPLAY LOGIC; IF YES, SHOW p50 – p55]**			
**p50**	ESS	Eaf	Diet provided suited our child’s medical needs
**p51**	ESS	Eaf	Parent able to stay close to child
**p52**	ESS	Eaf	Appropriate action taken to minimize exposure to infectious diseases
**p53**	CHC	Hac	Parent able to bond with child
**p54**	ESS	Eaf	Flexibility to decide who could be at child's bedside in the Intensive Care Unit
**p55**	SPC	Rb	Time to train a long-term caregiver to care for child before discharge
**Has your child been cared for at home in the last 12 months? [DISPLAY LOGIC; IF YES, SHOW p56 – p57]**			
**p56**	SPC	Emp	Enough consultations to support the care of child
**p57**	SPC	Rb	Avoided unnecessary hospitalizations

^a^*ESS* Efficient healthcare structures and standards**,** *SPC* Supporting parent caregivers**,** *CHC* Collaborative & holistic care, *PQ* Professional Qualities of healthcare workers

^b^*Rsc* Responsive and sensitive communication, *Pcom* Competency of healthcare delivery, *Emp* Empowering parent-caregivers, *Pss* Providing psychosocial support to parents and family, *Rb* Reducing caregiving stress and burdens, *Sdm* Shared decision-making, *Hac* Holistic approach to care for the child**,** *Amc* Accessible medical care, *Eaf* Effective administration and facilities, *Ccc* Coordination and continuity of care

### Pilot-testing results

Thirty parents completed the pilot-test (characteristics in Table 3). Summarized pilot-testing results are reported in Table 5. Parents took a median time of 16.6 minutes (Interquartile range: 11.0 – 26.7) to complete consent, sociodemographic data collection, and the three measures (PRECIOUS, QCPCI, and MPOC-20). Dropout rate was 25%, which is commonly seen in web surveys [46–48], suggesting that completing all three PaREMs in full-scale validation is feasible. Four PRECIOUS items had high non-response rates (‘Not Applicable’), ranging from 27% to 57%.

**Table 5:** Results of pilot-testing the PaRental Experience with care for Children with serIOUS illnesses (PRECIOUS) measure

	**Median (Interquartile range)**				
**Time to complete (minutes)**	16.6 (11.0 – 26.6)				
**Response Rate Statistics**	N (%)				
Invited respondents	70 (100)				
Eligible respondents	50 (71)				
Consenting respondents	40 (80)				
Dropouts	10 (25)				
**Scale responses**^**a**^	**Valid responses (N)**	**Mean**	**SD**	**Min**	**Max**
**PRECIOUS**					
Efficient healthcare structures and standards, 15 items	30	2.57	0.69	1.07	4.00
Supporting parent caregivers, 18 items	30	2.25	0.75	1.31	4.00
Collaborative & holistic care, 15 items	30	2.27	0.69	1.29	3.67
Professional Qualities of healthcare workers, 9 items	30	2.80	0.75	1.63	4.00
**Quality of children’s palliative care instrument (QCPCI)**^**b**^					
Connect with Families, 4 items	30	2.42	0.81	1	4
Involve Parents, 5 items	30	2.72	0.70	1.2	4
Share Information Among Health Professionals, 3 items	30	2.45	0.72	0.67	4
Support Siblings, 3 items	14	1.05	0.80	0	2.67
Global rating (Overall quality)	30	2.30	0.95	1	4
**Measure of Processes of Care (MPOC-20)**^**c**^					
Enabling & Partnership, 3 items	30	4.70	1.17	1	7
Providing General Information, 5 items	29	3.89	1.38	1	6.8
Providing Specific information about Child, 3 items	30	4.31	1.26	2.3	7
Coordinated & Comprehensive Care, 4 items	30	4.64	1.22	2.25	7
Respectful & supportive Care, 5 items	30	4.77	1.10	3	7

^a^QCPCI was created to assess the quality of pediatric palliative care and validated amongst children living with cancer. A respondent's data yield four scores, one for each of four subscales. MPOC is a self-report measure of parents’ perceptions of the extent to which the health services they and their child(ren) receive are family-centred

^b^QCPCI uses a 5-point Likert scale; *Never* = *0 / Rarely* = *1 / Sometimes* = *2 / Frequently* = *3 / Always* = *4*, and one additional 5-point global quality-of-care rating on a scale of *Poor* = *0 / Fair* = *1 / Good* = *2 / Very Good* = *3 / Excellent* = *4*. A respondent's data yield five scores, one for each of five subscales

^c^MPOC-20 uses a 7-point Likert scale ranging from *Not at All* = *1 / To a Very Small Extent* = *2 / To a Small Extent* = *3 / To a Moderate Extent* = *4 / To a Fairly Great Extent* = *5 / To a Great Extent* = *6 / To a Very Great Extent* = *7.* Items answered ‘Not applicable’ are assigned a value of ‘0’

All three PaREMs revealed current gaps in care. On QCPCI, overall QoC was rated as 2.3/4.0 on average, just above the ‘Good’ rating of 2.0. Parents reported other subscales between 2.42 – 2.72 out of 4.0. This range corresponded to frequencies between ‘Sometimes’ (2.0) and ‘Frequently’ (3.0). Sibling support was found to be ‘Rarely’ experienced with a mean score of 1.05 among the children with siblings (*n* = 14, 47%). The five MPOC-20 subscales showed that parental needs were ‘Sometimes’ (4.0) met with mean scores ranging from 3.89 – 4.77 out of 7.0. On PRECIOUS, the four subscales ranged from 2.25 – 2.80 out of 4.0, marginally above a frequency of ‘Sometimes’ (2.0). Results for each item of PRECIOUS (full results in Additional file 5) suggested similar gaps in care processes. Thirty-three percent of parents reported ‘Seldom’ having access to sufficient financial support for non-medical expenses, such as therapy. Emotional support for the child was also ‘Seldom’ experienced with a mean score of 1.08/4.0.

Table 6 presents Spearman’s ρ between the four PRECIOUS subscales, MPOC-20 subscales, and QCPCI subscales. The PRECIOUS subscales were significantly correlated with the Global Rating of QoC, MPOC-20 and QCPCI subscales, except for QCPCI’s Sibling Support and MPOC-20’s Providing General Information.

**Table 6:** Correlation of PRECIOUS scales with two similar measures in pilot-testing

Subscale^a^	Efficient healthcare structures and standards	Supporting parent caregivers	Collaborative & holistic care	Professional qualities of healthcare workers
**Supporting parent caregivers**	0.75*	1		
**Collaborative & holistic care**	0.66*	0.83*	1	
**Professional qualities of healthcare workers**	0.76*	0.85*	0.81*	1
**Global rating (Overall quality)**	0.54*	0.57*	0.66*	0.50*
**Quality of Children’s Palliative Care Instrument**				
**Connect with Families**	0.65*	0.69*	0.77*	0.62*
**Involve Parents**	0.88*	0.78*	0.77*	0.77*
**Share Information Among Health Professionals**	0.69*	0.62*	0.66*	0.52*
**Support Siblings**	0.44	0.08	0.16	-0.10
**Measure of Processes of Care**				
**Enabling & Partnership**	0.71*	0.60*	0.45*	0.54*
**Providing General Information**	0.36	0.38*	0.23	0.12
**Providing Specific Information about Child**	0.52*	0.41*	0.45*	0.41*
**Coordinated & Comprehensive Care**	0.56*	0.67*	0.51*	0.55*
**Respectful & supportive Care**	0.64*	0.70*	0.63*	0.63*

^*^p < 0.05

^a^Correlations presented are Spearman's ρ

#### Steering committee actions following pilot-testing

Among the four items with high non-response rates, the committee decided to retain three for future testing with a larger sample size due to the small sample of the pilot-test. One item was removed, “…asked us if we wanted to contribute to the community of seriously ill children, such as letting us support other families or participating in research” (non-response rate of 38%). We created an infographic (Additional file eFigure 1) with selected results and distributed it to the study team and parents who had agreed to be recontacted. These recipients were invited to provide any additional feedback. Finally, based on the steering committee’s discussions, the instructions of PRECIOUS were revised for simplicity. For example, “Sharing your experience will help in improving the quality of care your family and future families will receive” was shortened to “Sharing your views will help improve the quality of care in the future”.

## Discussion

To enhance QoC for seriously ill children, service providers should regularly evaluate care processes that parents consider important. Existing PaREMs for seriously ill children typically focus on specific care settings, resulting in a fragmented assessment of QoC [19]. To address this gap, we have developed the PRECIOUS measure to encourage closer collaboration between and within care teams and settings. PRECIOUS is a comprehensive PaREM that provides HCWs with a holistic view of parental priorities, comprising of 56 specific, well-defined QoC processes that are categorized into four themes (Efficient healthcare structures and standards, Supporting parent caregivers, Collaborative & holistic care, and Professional qualities of healthcare workers) and 10 subthemes. It is applicable to parents of seriously ill children (<18 years old) across multiple care settings and throughout their illness trajectory. Designed as a process measure to capture parental experience, PRECIOUS complements outcome measures and quality indicators related to effectiveness and safety, thereby highlighting potential areas for intervention.

Developing PRECIOUS also revealed that parents of seriously ill children often share common challenges and experiences, irrespective of the child patient’s specific illness or age. This aligns with broader findings indicating shared parental experiences across various clinical scenarios. For instance, research on children with medical complexity [49] and tracheotomized children [50, 51] showed that despite varied diagnoses, the challenges faced by parents are consistent. These findings highlight the value of a versatile measure capable of evaluating care quality across the spectrum of pediatric serious illnesses. PRECIOUS subscales correlated with other QoC measures developed for the illness trajectory —MPOC-20 for (community care) and QCPCI for (hospital care), suggesting that it may be applicable both across illness categories and settings. Furthermore, these correlations highlight the interconnectedness of care experiences, underscoring the importance of introducing comprehensive measures like PRECIOUS in expanding health and social care systems [52–54].

In considering the limitations of our measure, it is crucial to acknowledge the context-specific nature of our findings, which primarily pertain to the healthcare system in a high-income and socio-culturally diverse Southeast Asian country. While PRECIOUS was developed with a global perspective in mind and involved international experts in the Delphi expert panel, whether it can be applied in different healthcare settings is unknown at present [55, 56]. Diversity in pediatric healthcare delivery models and cultural expectations regarding child-patient care across settings needs careful consideration and potential adjustment to items to address context-dependent practices (e.g., availability of universal coverage of insurance, access to general and/or tertiary healthcare) and sociocultural norms (i.e., cultural and religious beliefs in dealing with disability) [57]. Importantly, this paper presents key measure development steps but PRECIOUS should be subjected to further validation, continuous assessment, and refinement before and after it is implemented in varied contexts.

Future research should adapt and validate the measure across different healthcare environments, thereby enhancing its universal applicability and effectiveness in improving care for seriously ill children globally. Exploring differences in parental experiences based on the child's phase of illness and whether the child is communicative could also be part of future efforts. Lastly, developing a shorter version is a key future goal because the comprehensive nature of PRECIOUS may make it cognitively burdensome. Having established the content validity and feasibility of the 56-item PRECIOUS measure, our next step is to establish its measurement properties through a larger study.

## Conclusions

The 56-item PaREM PRECIOUS has been developed to (i) capture key care processes important to parents of seriously ill children; (ii) be applicable across contexts (such as geographic location or care setting) and different HCWs, and (iii) be relevant throughout the illness trajectory for children suffering from a range of serious illnesses. When fully validated and integrated into routine care, PRECIOUS will provide a standardized evaluation of QoC processes across diverse healthcare settings over time.

### Supplementary Information

**Additional file 1.**

**Additional file 2.**

**Additional file 3.**

**Additional file 4.**

**Additional file 5.**

**Additional file 6: eFigure 1.**

## Data Availability

The datasets used and/or analyzed during the current study are available from the corresponding author on reasonable request.

## Abbreviations

QoC: Quality of Care
PRECIOUS: PaRental Experience with care for Children with serIOUS illnesses
HCWs: Healthcare workers
PaREMs: Parent-Reported Experience Measures
MPOC: Measure of Processes of Care
QCPCI: Quality of Children’s Palliative Care Instrument
