# Supplementary material for: Development of the Parental Experience with Care for Children with Serious Illnesses (PRECIOUS) quality of care measure
Source: BMC Palliat Care. 2024 Mar 8;23:66. doi: 10.1186/s12904-024-01401-x (PMC10921687; doi:10.1186/s12904-024-01401-x)
Supplement: Supplementary file 1 — Additional file 1. [file 12904_2024_1401_MOESM1_ESM.pdf]

Additional file 1. Delphi expert panel review handout

PaRental Experience with care for Children with serIOUS illnesses (PRECIOUS)

Recap on PRECIOUS

Purpose of Delphi review

PRECIOUS theoretical framework

Delphi procedures

What you will do

FAQs

Recap on PRECIOUS

What is PRECIOUS? The PaRental Experience with care for Children with serIOUS illnesses (PRECIOUS) is a new measure to assess parent's experiences with healthcare for their child with a serious illness*.

Why do we need these measures? These standardized tools enable parents to systematically evaluate the processes of care and interpersonal aspects of their experience. We can use these measures to report and benchmark quality of care across service delivery settings and over time. This helps us to design high-quality intervention plans and evaluate them to improve quality of care.

**Serious illnesses refer to a spectrum of conditions carrying high risk of mortality, negatively impacting daily function or quality of life, or excessively straining caregivers.*
For further information about these conditions, please visit the Together for Short Lives page.

Purpose of Delphi review

What is this review for? The panel will assess how well ***PRECIOUS*** captures parents' experience with various care processes delivered to them and their seriously ill child, and to improve the clarity of items.

How was PRECIOUS developed? In a prior study, we explored the key care processes from the perspective of parents from over 30 families with seriously ill children. The study led to the development of 65 process indicators across 10 subthemes, and a framework of high-quality care to guide quality improvement. These findings are applicable throughout the disease trajectory and various service providers.

What will experts be reviewing? The items in PRECIOUS describe ***care processes* –** they describe parents' perspective on key services and behaviors delivered by healthcare workers in a specific and well-defined way. This perspective may not be the same as your own opinions. However, we hope you can review the items using both your knowledge and from the perspective of this parent-driven framework ***(see next page)***.

Theoretical framework

PRECIOUS encompasses the enduring care processes prioritized by parents of young children (<8 years old) experiencing a serious illness.

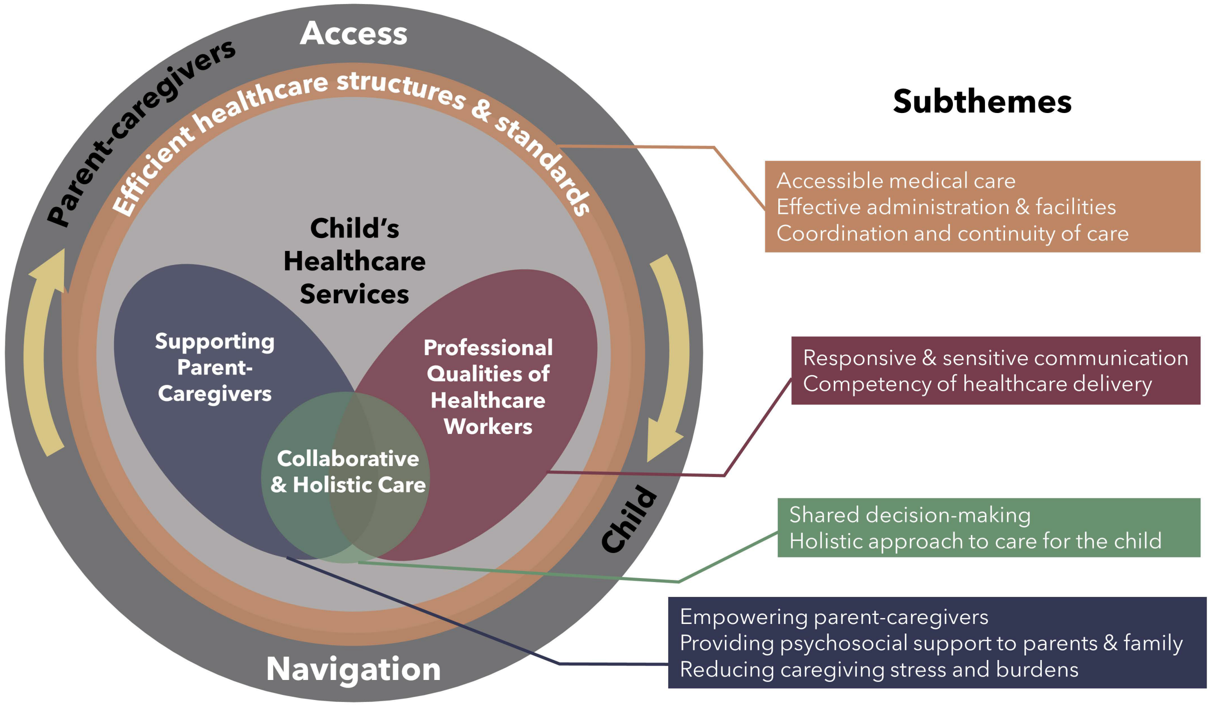

Themes, subthemes, and number of quality of care items resulting from the qualitative study

Theme	Subtheme	Number of items
1. Professional Qualities of healthcare workers	1.1 Responsive and sensitive communication	9
	1.2 Competency of healthcare delivery	6
2. Supporting parent caregivers	2.1 Empowering parent-caregivers	6
	2.2 Providing psychosocial support to parents and family	7
	2.3 Reducing caregiving stress and burdens	6
3. Collaborative & holistic care	3.1 Shared decision-making	5
	3.2 Holistic approach to care for the child	8
4. Efficient healthcare structures and standards	4.1 Accessible medical care	6
	4.2 Effective administration and facilities	5
	4.3 Coordination and continuity of care	6

Delphi procedures

Facilitation round 1
- 15th April – 22nd April

Facilitation round 2
- 6th May – 13th May

Facilitation round 3
- 27th May – 6th June

Delphi round 1
- **1st April – 15th April**
- Experts rate items

→ No expert panel action

Delphi round 2
- **22nd April – 6th May**
- Experts rate items

→ No expert panel action

Delphi round 3
- **13th May – 27th May**
- Experts rate items

→ Expert panel participation completed

Final expert-modified measure
- No expert panel action

• Each round will be open for 2 weeks, and **you can access the link anytime** during that window. You can **leave the survey and resume your progress later,** so long as you return on the **same browser** where you started the survey. You can also edit your previous responses anytime.
• There will be 1 week between rounds for facilitation (modifications, aggregate decisions, prepare next version). You will not have to take any action.
• To complete the Delphi process, experts should respond across all three rounds.

Duke NUS Medical School | Lien Centre for Palliative Care

What you will be doing

You will rate each item across 10 subthemes. A free-text response will be available if you wish to elaborate your thought process or suggest changes.

Example of rating:

Are these appropriate items to capture **Accessible Medical Care**? Are the response options appropriate?

	No, not appropriate	Yes, with changes to item or response options	Yes, no changes to recommend
I have access to multi-disciplinary expertise in my child's range of condition(s) (e.g., medical nursing, allied health professionals, specialists, social workers).<br>***Response options: Never / Seldom / Sometimes / Usually / Always***	○	○	○
I have access to on-demand assistance and advice from healthcare workers.<br>***Response options: Never / Seldom / Sometimes / Usually / Always***	○	○	○

The facilitators will make changes to subsequent versions based on experts' input and/or consensus each round.

*Consensus is defined as >70% (for inclusion)
Each item's aggregate expert ranking and facilitators' modifications will be shown in each subsequent Delphi round.

We look forward to a meaningful review.

Link to Round 1 will be emailed to you on 1st April 2022.

Thank you for sharing your voice.

Other Frequently Asked Questions (FAQs)

Why are we focusing on parents? Evaluating parental perspectives on care is critical in designing high quality treatment plans for seriously ill young children. Parents play uniquely intimate and intense roles over the entire disease trajectory.

Why develop an experience measure? The development of PRECIOUS will enable standardized evaluation of the quality of care, and measure the effect of any interventions for families of seriously ill children.

Why are current PaREMs insufficient? A previous scoping review we conducted found a lack of PaREMs which are applicable to multiple providers and varying lengths of care. Given the multidisciplinary nature of pediatric care and the often-unpredictable disease trajectories of seriously ill children, measures which are narrow in scope are unable to provide a comprehensive assessment of quality of care.

How is PRECIOUS different from existing parent-reported measures? PRECIOUS is uniquely broad in scope. It is the first PaREM applicable to *parents* of seriously ill children throughout the entire disease trajectory *and* multiple service providers. Hence, PRECIOUS provides opportunities for targeted interventions to improve the quality of care along the whole trajectory of a family's care journey, and enables standardized measurement to improve care coordination across all service providers forming a child's care network.
