# Supplementary material for: Development of the Parental Experience with Care for Children with Serious Illnesses (PRECIOUS) quality of care measure
Source: BMC Palliat Care. 2024 Mar 8;23:66. doi: 10.1186/s12904-024-01401-x (PMC10921687; doi:10.1186/s12904-024-01401-x)
Supplement: Supplementary file 2 — Additional file 2. [file 12904_2024_1401_MOESM2_ESM.pdf]

Additional file 2. Full results of round 1 and 2 of Delphi expert panel review

THEME	SUBTHEME		ITEM FROM ROUND 1 (PROCESSES OF CARE)	Yes, appropriate	n	Yes, with changes	n	Not appropriate	n	Total	REVISED ITEM FOR ROUND 2 (PROCESSES OF CARE)	Expert comments	
~~Effective~~ Efficient healthcare structures & standards	Accessible Medical Care	1	I have access to multi-disciplinary expertise in my child's range of condition(s) (e.g., medical nursing, allied health professionals, specialists, social workers, etc). Response options: Never / Seldom / Sometimes / Usually / Always	88%	21	13%	3	0%	0	24	I ~~have~~ **can easily** access ~~to~~ **a range of healthcare workers in many disciplines** ~~multi-disciplinary expertise~~ **to meet my child's needs** ~~in my child's range of condition(s)~~ (e.g., **doctors, nurses, pharmacists, social workers, therapists** ~~medical nursing, allied health professionals, specialists, social workers, etc~~).		
		2	I have access to on-demand assistance and advice from healthcare workers. Response options: Never / Seldom / Sometimes / Usually / Always	67%	16	33%	8	0%	0	24	I ~~have~~ **can easily receive** ~~access to on-demand assistance and~~ advice from healthcare workers **when I need it.**		
		3	Healthcare workers are approachable when I seek their advice. Response options: Never / Seldom / Sometimes / Usually / Always	83%	20	17%	4	0%	0	24	**I feel comfortable asking my child's h**ealthcare workers ~~are approachable when I seek their~~ **for** advice **about my child's care**.		
		4	Healthcare workers offer convenient ways to obtain and manage my child's medical equipment and supplies. Response options: Never / Seldom / Sometimes / Usually /	79%	19	17%	4	4%	1	24	Healthcare workers ~~offer convenient ways to~~ **help me to** obtain ~~and manage~~ my child's medical equipment and supplies.		
		5	Healthcare workers facilitate the process of acquiring high-cost medical equipment (e.g. ventilator, suction machine, buggy) that my child needs. Response options: Never / Seldom / Sometimes / Usually / Always / My child does not need high-cost equipment	75%	18	17%	4	8%	2	24	Healthcare workers **help me in** ~~facilitate~~ the process of acquiring high-cost medical equipment (e.g. ventilator, suction machine, buggy) for my child ~~that my child needs~~.		
		6	I receive financial support to support my child's medical expenses that is based on an individualized assessment of my family's needs. Response options: Never / Seldom / Sometimes / Usually / Always	71%	17	29%	7	0%	0	24	I receive **adequate** financial support **for** ~~to support~~ my child's medical expenses ~~that is based on an individualized assessment of my family's needs~~. ***Response options: Yes / No***		
	Effective administration and facilities	1	Healthcare workers offer flexibility in visitation for my child when he/she is critically ill (e.g. allowing multiple caregivers at bedside). Response options: Strongly Disagree / Disagree / Neither Agree nor Disagree / Agree / Strongly Agree / Not applicable to my child	75%	18	13%	3	13%	3	24	Healthcare workers offer flexibility in **the number of caregivers allowed at bedside** ~~visitation for my child~~ when **my child needs intensive care** ~~he/she is critically ill (e.g. allowing multiple caregivers at bedside)~~.		
			~~I have the ability to choose my child's healthcare workers. Response options: Strongly Disagree / Disagree / Neither Agree nor Disagree / Agree / Strongly Agree~~	46%	11	21%	5	33%	8	24	**ELIMINATE**		
		2	My child is fast tracked at Children's Emergency because of his/her chronic and complex needs. Response options: Never / Seldom / Sometimes / Usually / Always / My child has not been to Children's Emergency	63%	15	25%	6	13%	3	24	My child **is attended to without undue delay** ~~fast tracked~~ **when we present at the emergency department** ~~at Children's Emergency because of his/her chronic and complex needs~~.		
		3	The food provided in hospital is suited to my child's unique needs or preferences. Response options: Never / Seldom / Sometimes / Usually / Always / Not applicable to my	71%	17	8%	2	21%	5	24	The food provided in **healthcare facilities** ~~hospital~~ **can be modified to** ~~is suited to~~ my child's ~~unique~~ needs ~~or preferences~~.		
		4	Healthcare workers give me a private and comfortable space to be close to my child when he/she is admitted to hospital or hospice. Response options: Never / Seldom / Sometimes / Usually / Always	71%	17	17%	4	13%	3	24	~~Healthcare workers give me a private and comfortable space to be~~ **I am able to stay** close to my child when he/she is admitted to hospital or hospice.		
		5	Healthcare workers ensure the environment in which my child is cared for is safe and clean. Response options: Never / Seldom / Sometimes / Usually / Always	79%	19	17%	4	4%	1	24	Healthcare workers ~~ensure the environment in which~~ **take appropriate action to minimize** my child's ~~is~~ **exposure to other diseases when he/she is admitted in healthcare facilities** ~~is safe and clean~~.		
	Coordination and continuity of care	1	Healthcare workers work as a team towards the same goals for my child's care. Response options: Never / Seldom / Sometimes / Usually / Always	92%	22	8%	2	0%	0	24	NO CHANGE		
		2	I have a main healthcare worker or team who has consistent oversight over my child's medical needs. Response options: Yes / No / I don't know	92%	22	8%	2	0%	0	24	I have a main healthcare worker **who** or team **who consistently oversees** ~~has consistent oversight over~~ my child's medical needs.		
		3	I have a healthcare worker or team who coordinates my child's care between different disciplines and services. Response options: Yes / No / I don't know	92%	22	8%	2	0%	0	24	I have a healthcare worker **who** or team ~~who~~ **which** coordinates my child's care between different disciplines, ~~and~~ services**, and agencies**.		
		4	I receive the same information from different healthcare workers. Response options: Never / Seldom / Sometimes / Usually / Always	67%	16	33%	8	0%	0	24	I receive **consistent** ~~the same~~ information from different healthcare workers.		
		5	Healthcare workers ensure a smooth transition of care across the different settings my child receives care (e.g., NICU to PICU, Private to Public, going home, hospital admission). Response options: Never / Seldom / Sometimes / Usually / Always	88%	21	13%	3	0%	0	24	Healthcare workers ensure a smooth transition of care across the different **healthcare** settings ~~my child receives care~~ (e.g., NICU to PICU, Private to Public **facilities**, going home, hospital admission).		
		6	Healthcare workers make sure my child's appointments are well coordinated to reduce our hospital visits. Response options: Never / Seldom / Sometimes / Usually / Always	92%	22	8%	2	0%	0	24	Healthcare workers ~~make sure~~ **coordinate** my child's appointments ~~are well coordinated~~ to reduce our hospital visits.		
Professional Qualities of HCWs	Responsive and sensitive communication	1	Healthcare workers present themselves in an honest and transparent way. Response options: Strongly Disagree / Disagree / Neither Agree nor Disagree / Agree / Strongly Agree	79%	19	17%	4	4%	1	24	Healthcare workers present themselves in an honest ~~and transparent~~ way.		
		2	I trust my child's healthcare workers. Response options: Strongly Disagree / Disagree / Neither Agree nor Disagree / Agree / Strongly Agree	100%	24	0%	0	0%	0	24	**Healthcare workers make an effort to build a trusting relationship with me.** ~~I trust my child's healthcare workers.~~		
		3	Healthcare workers take responsibility for my child's wellbeing and things that happen to my child under their care. Response options: Never / Seldom / Sometimes / Usually /	83%	20	17%	4	0%	0	24	Healthcare workers take responsibility for my child's wellbeing ~~and things that happen to my child~~ **when he/she is** under their care.		
		4	Healthcare workers respect my right to information on my child. Response options: Never / Seldom / Sometimes / Usually / Always	88%	21	13%	3	0%	0	24	Healthcare workers **support** ~~respect~~ my right to information **about** ~~on~~ my child **by ensuring I am always fully informed**.		
		5	Healthcare workers give me information on my child's condition in a timely manner. Response options: Never / Seldom / Sometimes / Usually / Always	92%	22	8%	2	0%	0	24	NO CHANGE		
		6	Healthcare workers communicate with me in a sensitive way. Response options: Never / Seldom / Sometimes / Usually / Always	92%	22	8%	2	0%	0	24	Healthcare workers communicate with me in a ~~sensitive~~ way **that is sensitive to my needs**.		
		7	Healthcare workers communicate with me in a way that I can understand. Response options: Never / Seldom / Sometimes / Usually / Always	100%	24	0%	0	0%	0	24	NO CHANGE		
		8	Healthcare workers give me enough time to think about decisions for my child's care. Response options: Never / Seldom / Sometimes / Usually / Always	100%	24	0%	0	0%	0	24	NO CHANGE		
		9	Healthcare workers have a good sense of urgency so that I am not unneccessarily stressed. Response options: Strongly Disagree / Disagree / Neither Agree nor Disagree / Agree / Strongly Agree	58%	14	29%	7	13%	3	24	Healthcare workers have a**n appropriate** ~~good~~ sense of urgency **when communicating with me** so **that additional stress is not created** ~~I am not unneccessarily stressed~~ **(e.g. not calling multiple times throughout the day for non-urgent issues, informing me about critical news quickly).**		
		10	Healthcare workers show me genuine care and sincerity. Response options: Never / Seldom / Sometimes / Usually / Always	92%	22	8%	2	0%	0	24	NO CHANGE		
		11	Healthcare workers are respectful of my spiritual or religious customs and beliefs. Response options: Never / Seldom / Sometimes / Usually / Always / Not applicable to me	96%	23	4%	1	0%	0	24	NO CHANGE		
	Competency of healthcare delivery	1	Healthcare workers have the necessary knowledge, skills and experience in their fields to meet my child's and family's needs. Response options: Strongly Disagree / Disagree /	79%	19	21%	5	0%	0	24	Healthcare workers **present their** ~~have the necessary~~ knowledge, skills and experience **so that I am confident they can meet my child's needs** ~~in their fields to meet my child's and~~		
		2	Healthcare workers avoid unnecessary treatments and investigations on my child. Response options: Never / Seldom / Sometimes / Usually / Always	83%	20	17%	4	0%	0	24	Healthcare workers avoid unnecessary treatments and investigations on my child. **Response options: Strongly Disagree / Disagree / Neither Agree nor Disagree / Agree / Strongly**		
		3	Healthcare workers deliver timely medical care for my child. Response options: Never / Seldom / Sometimes / Usually / Always	83%	20	17%	4	0%	0	24	Healthcare workers ~~deliver~~ **provide** ~~timely~~ medical care for my child **without undue delay**.		
		4	Healthcare workers are able to identify and solve my child's medical issues. Response options: Never / Seldom / Sometimes / Usually / Always	71%	17	29%	7	0%	0	24	Healthcare workers ~~are able to~~ **do all they can to** identify and ~~solve~~ **treat** my child's medical issues.		
		5	Healthcare workers manage my child's physical symptoms to make sure my child is comfortable. Response options: Never / Seldom / Sometimes / Usually / Always	88%	21	13%	3	0%	0	24	NO CHANGE		
Supporting parent caregivers	Empowering parent-caregivers	1	Healthcare workers tell me what to look out for so that I know what to do when he/she is unwell without needing to go to the hospital. Response options: Never / Seldom / Sometimes / Usually / Always	79%	19	21%	5	0%	0	24	Healthcare workers tell me what to look out for so that I know what to do when **my child** ~~he/she~~ is unwell~~, without needing to go to the hospital~~.		
		2	Healthcare workers equip me with skills so that I can confidently care for my child out of the hospital. Response options: Never / Seldom / Sometimes / Usually / Always	92%	22	8%	2	0%	0	24	NO CHANGE		
		3	Healthcare workers acknowledge and affirm my efforts in caring for my child. Response options: Never / Seldom / Sometimes / Usually / Always	100%	24	0%	0	0%	0	24	NO CHANGE		
		4	Healthcare workers give me opportunities to bond with my child during hospital or hospice admissions. Response options: Never / Seldom / Sometimes / Usually / Always	96%	23	4%	1	0%	0	24	NO CHANGE		
		5	Healthcare workers make home visits to give my child medical treatment or care. Response options: Never / Seldom / Sometimes / Usually / Always	58%	14	25%	6	17%	4	24	Healthcare workers make home visits to **support my caregiving by checking that my child is well taken care of at home** ~~give my child medical treatment or care~~. Response options: Never / Seldom / Sometimes / Usually / Always **/ Not applicable to my child**		
		6	Healthcare workers give me opportunities to advocate or speak up for my child and myself. Response options: Never / Seldom / Sometimes / Usually / Always	96%	23	0%	0	4%	1	24	NO CHANGE		
		7	Healthcare workers provide me with opportunities to give back to the special needs community e.g. supporting other families, research and funding. Response options: Never / Seldom / Sometimes / Usually / Always / I prefer not to be involved	79%	19	17%	4	4%	1	24	Healthcare workers provide me with opportunities to give back to the special needs community **when I approach them** e.g. **letting me** support~~ing~~ other families, **participate in** research and **raising** fund~~s~~ing.		
	Providing psychosocial support to parents AND FAMILY	1	Healthcare workers support my hopes for my child. Response options: Never / Seldom / Sometimes / Usually / Always	100%	24	0%	0	0%	0	24	NO CHANGE		
		2	Healthcare workers prepare me for what may lie ahead. Response options: Never / Seldom / Sometimes / Usually / Always	96%	23	4%	1	0%	0	24	NO CHANGE		
		3	Healthcare workers provide me with a compassionate listening ear. Response options: Never / Seldom / Sometimes / Usually / Always	100%	24	0%	0	0%	0	24	NO CHANGE		
		4	Healthcare workers give me emotional and physical space to be alone when I have received difficult news about my child. Response options: Strongly Disagree / Disagree / Neither Agree nor Disagree / Agree / Strongly Agree	75%	18	17%	4	8%	2	24	Healthcare workers give me **appropriate time and** ~~emotional and physical~~ space **if I wish** to be alone ~~when I have~~ **after** receiv~~ed~~**ing** difficult news about my child.		
		5	Healthcare workers give me access to parent support networks. Response options: Never / Seldom / Sometimes / Usually / Always	83%	20	17%	4	0%	0	24	Healthcare workers **facilitate my** ~~give me~~ access to parent support networks.		
		6	Healthcare workers attend to my entire family's needs. Response options: Never / Seldom / Sometimes / Usually / Always / Not applicable to my family	71%	17	25%	6	4%	1	24	Healthcare workers **assess and where possible help with our** ~~attend to my entire~~ family's **psychosocial** needs **resulting from my child's condition**.		
	Reducing caregiving stress and burdens	1	Healthcare workers give me options for someone skilled to take care of my child so that I can take a break if I need it. Response options: Never / Seldom / Sometimes / Usually /	83%	20	17%	4	0%	0	24	Healthcare workers **help me find** ~~give me~~ options for someone skilled to take care of my child so that I can take a break if I need it.		
		2	Healthcare workers give me practical suggestions on how we can reduce our family's financial burden. Response options: Never / Seldom / Sometimes / Usually / Always	83%	20	13%	3	4%	1	24	Healthcare workers give me practical suggestions on how I ~~we~~ can reduce ~~our~~ **my child's medical costs** ~~family's financial burden~~.		
		3	Healthcare workers provide guidance to available resources to reduce my family's financial burden. Response options: Never / Seldom / Sometimes / Usually / Always	75%	18	21%	5	4%	1	24	Healthcare workers **guide me to** ~~provide guidance to~~ available resources to reduce my family's financial burden.		
		4	Healthcare workers do their best to avoid my child's hospitalization. Response options: Never / Seldom / Sometimes / Usually / Always	75%	18	17%	4	8%	2	24	Healthcare workers do their best to avoid my child's **unnecessary and unplanned** hospitalization.		
		5	Healthcare workers offer specialized transport for my child with mobility needs. Response options: Never / Seldom / Sometimes / Usually / Always / My child does not have mobility needs	71%	17	21%	5	8%	2	24	Healthcare workers offer **information for us to find** specialized transport for my child ~~with~~ **who has** mobility needs.		
Personalized Collaborative and holistic care	Holistic approach to care for the child	1	Healthcare workers put in effort to foster a personal relationship with my child. Response options: Never / Seldom / Sometimes / Usually / Always	100%	24	0%	0	0%	0	24	NO CHANGE		
		2	Healthcare workers provide us with allied health support (e.g. therapists) to meet my goals for my child. Response options: Never / Seldom / Sometimes / Usually / Always	92%	22	8%	2	0%	0	24	Healthcare workers provide us with **appropriate** allied health support (e.g. therapists) to meet my goals for my child**'s development**.		
		3	Healthcare workers create a child-friendly atmosphere in hospital. Response options: Never / Seldom / Sometimes / Usually / Always	88%	21	8%	2	4%	1	24	Healthcare workers **do their best to** create a child-friendly atmosphere in hospital **or hospice**.		
		4	Healthcare workers give my child emotional support and encouragement. Response options: Never / Seldom / Sometimes / Usually / Always / Not applicable to my child	88%	21	13%	3	0%	0	24	Healthcare workers **provide** ~~give~~ my child **with** emotional support ~~and encouragement~~		
		5	I have access to suitable facilities or services for my child's play and engagement. Response options: Strongly Disagree / Disagree / Neither Agree nor Disagree / Agree /	83%	20	17%	4	0%	0	24	**Healthcare workers facilitate my child's** ~~I have~~ access to **appropriate** ~~suitable~~ facilities or services for ~~my child's~~ play and engagement.		
		6	Healthcare workers facilitate my child's access to special needs schools. Response options: Strongly Disagree / Disagree / Neither Agree nor Disagree / Agree / Strongly Agree / Not applicable to my child	88%	21	13%	3	0%	0	24	Healthcare workers facilitate my child's access to special needs schools **when I ask**. Response options: **Yes / No /** Not applicable to my child		
	Shared decision-making	1	Healthcare workers give me complete information on all management options for my child so that I can make informed decisions. Response options: Never / Seldom / Sometimes / Usually / Always	83%	20	13%	3	4%	1	24	NO CHANGE		
		2	Healthcare workers explain both the benefits and burdens of technology and procedures on my child. Response options: Never / Seldom / Sometimes / Usually / Always	75%	18	17%	4	8%	2	24	Healthcare workers **clearly** explain **the pros and cons of different medical** ~~both the benefits and burdens of~~ technologi**es**~~y~~ and procedures (Continuous positive airway pressure (CPAP), tracheostomy) on my child **so that I am aware of the effects it will have on my child's daily life**.		
		3	A palliative care team or specialist(s) is an active part of my child's treatment plan. Response options: Yes / No / I don't know	63%	15	33%	8	4%	1	24	A palliative care team or specialist(s) **participates in** ~~is an active part of~~ my child's treatment plan.		
		4	[If parent responds yes to prior item] Palliative care team or specialist(s) was introduced at an appropriate time. Response options: Too early / Too late / Appropriate time	88%	21	13%	3	0%	0	24	[If parent responds yes to prior item] **Healthcare workers introduced t**he palliative care team or specialist(s) ~~was introduced~~ at an appropriate time. **Response options: Yes / No**		
		5	Healthcare workers discussed the role of comfort care when I was given a poor prognosis of my child's condition. Response options: Yes / No / I don't know / Not applicable to my	75%	18	25%	6	0%	0	24	Healthcare workers discussed **how the scope of** ~~the role of comfort~~ **care can be tailored to provide comfort for my child** when I was given a poor prognosis of my child's condition.		
		6	Healthcare workers are responsive to my inputs regarding my child's care. Response options: Never / Seldom / Sometimes / Usually / Always	79%	19	21%	5	0%	0	24	Healthcare workers **listen to** ~~are responsive to~~ my inputs regarding my child's care.		
		7	Healthcare workers respect my right to make decisions for my child's treatments. Response options: Never / Seldom / Sometimes / Usually / Always	83%	20	17%	4	0%	0	24	**I am as involved as much as I wish to be in decision-making about my child's treatment.** ~~Healthcare workers respect my right to make decisions for my child's treatments.~~		
		8	Healthcare workers recommend and manage my child's care while considering my family's preferences. Response options: Never / Seldom / Sometimes / Usually / Always	83%	20	13%	3	4%	1	24	Healthcare workers ~~recommend and manage~~ **provide care to** my child's ~~care~~ while considering my family's preferences **for treatments**.		

THEME	SUBTHEME		Round 2 REVISED WORKING ITEM (PROCESSES)	Yes, appropriate	n	Not appropriate	n	Total			Round 3 REVISED WORKING ITEM (PROCESSES)	Expert comments (if any)	Facilitator Response (if any)
Efficient healthcare structures & standards	Accessible Medical Care	1	I can easily access a range of healthcare workers in many disciplines to meet my child's needs (e.g., doctors, nurses, pharmacists, social workers, therapists etc).	91.67%	22	8.33%	2	24			Unchanged	Not every discipline / department are accessible<br>complex wording	No changes made in light of majority vote; most panelists found item appropriate.
		2	I can easily receive advice from healthcare workers when I need it.	91.67%	22	8.33%	2	24			I can easily ~~receive~~ **get** advice from healthcare workers when I need it.	I can easily access advice from healthcare workers<br>Seems similar to the item below, but the item below feels more patient/family centered<br>Is the question looking at ease of path to receive or readily receive / receive timely advice which maybe time based focused<br>I can easily receive advice for my child from healthcare workers when I need it.<br>get better than receive	For clarity, 'receive' replaced with 'get'.
		3	I feel comfortable asking my child's healthcare workers for advice about my child's care.	91.67%	22	8.33%	2	24			**Healthcare workers make me** ~~I~~ feel comfortable asking ~~my child's healthcare workers~~ for advice about my child's care.	This one seems to be more about relationships and trust than accessibility<br>Is this asking from the parent's comfort vs My child's healthcare worker makes me feel comfortable to ask advice about my child's care.<br>Why say "my child's healthcare workers" only for this item? (and say not in the previous or next item)	Suggested changes incorporated.
		4	Healthcare workers help me to obtain my child's medical equipment and supplies.	91.67%	22	8.33%	2	24			Healthcare workers **provide the guidance I need** ~~help me~~ to obtain my child's medical equipment and supplies.	Does not quite assess accessibility. Prescription of medical equipment is required of healthcare workers. Procurement and purchase may differ in different models of care, eg direct from vendors, which can be equally accessible.<br>The tend to help within their scope of work<br>Not help to obtain but rather advise on how to obtain, incase it breeds	Changes made for clarity.
		5	Healthcare workers help me in the process of acquiring high-cost medical equipment (e.g. ventilator, suction machine, buggy) for my child.	87.50%	21	12.50%	3	24			**ELIMINATE (due to high overlap with the construct of the previous indicator)**	Healthcare professionals help me in the process of acquiring appropriate medical equipment for my child (it may not be high cost but it needs to be appropriate)<br>This feels similar to the item above<br>...through or with the process...<br>Unclear about intent or extent. "help me in the process.." as in provide $ or faclitate application for financial assistance.<br>drop on the process	Combined with item above and eliminated due to high overlap.
		6	I receive adequate financial support for my child's medical expenses.<br>Response options: Yes / No	91.67%	22	8.33%	2	24			I **have access to** ~~receive adequate~~ financial support for my child's medical expenses **if needed**.<br>Response options: Yes / No **/ I do not need financial assistance**	I have access to financial support for my child's medical expenses if needed. (financial support may come from families, friends, etc). The question may need to be more specific on the source of help.<br>In keeping with the wording of the sub-theme of 'access,' perhaps "I have access to adequate financial support for my child's medical expenses."<br>Qualify where eligible. Unless this question is about perceptions. Most would say not enough in that case.	Most suggested changes incorporated.
	Effective administration and facilities		**Round 2 REVISED WORKING ITEM (PROCESSES)**	**Yes, appropriate**	**n**	**Not appropriate**	**n**	**Total**			**Round 3 REVISED WORKING ITEM (PROCESSES)**		
		1	Healthcare workers offer flexibility in the number of caregivers allowed at bedside when my child needs intensive care.	83.33%	20	16.67%	4	24			Healthcare workers offer flexibility in the number of caregivers allowed at bedside when my child **is in the** ~~needs~~ intensive care **unit**.	Instead of "needs intensive care", how about "is critically ill?<br>Healthcare institutions offer flexibility in the number of caregivers allowed at the bedside in times of need when my child is in the intensive care unit. (effective	Pertaining to concerns over institutional policies and the inapplicability to wider setting: item is retained as HCWs are given certain autonomy to allow caregivers when child is in the ICU in Singapore. Furthermore,
		2	My child is attended to without undue delay when we present at the emergency department.	91.67%	22	8.33%	2	24			Unchanged	Maybe this fits better in the previous one about accessibility?<br>The word "undue" is important<br>As parents may have varied expectations...Will the answers be in a ilkert format?<br>Am not sure if general respondents will understand the use of word undue	No changes made in light of majority vote; most panelists found item appropriate.
		3	The food provided in healthcare facilities can be modified to suit my child's unique needs.	87.50%	21	12.50%	3	24			The food provided in **hospital or hospice** ~~healthcare facilities~~ can be modified to suit my child's unique needs.	its good to have more variety for the children to make them more appetite in eating the meal. Especially western food with a few choices.q<br>HCP are open to discussions with me on type of nutritional/supplementary diets	
		4	I am able to stay close to my child when he/she is admitted to hospital or hospice.	91.67%	22	8.33%	2	24			Unchanged	If this item is retained, I might suggest revising the wording, as "stay close" seems vague.	No changes made in light of majority vote; most panelists found item appropriate.
		5	Healthcare workers take appropriate action to minimize my child's is exposure to other diseases when he/she is admitted in healthcare facilities.	79.17%	19	20.83%	5	24			Healthcare workers take appropriate action to minimize my child's ~~is~~ exposure to other diseases when he/she is admitted in ~~healthcare facilities~~ **hospital**.	minimize my child's exposure<br>I am not sure if this is only applicable to seriously ill patients. It should apply to ALL patients.<br>Healthcare workers take appropriate action to minimise my child's exposure to other diseases when he/she is admitted in healthcare facilities<br>hospital or hospice instead of healthcare facilties, simialr to term used in previous item?<br>ok to include, but edit awkward wordin	Most suggested changes incorporated.
			**[New indicators suggested in Round 1 for testing in Round 2]**	**Yes, appropriate**	**n**	**Yes, with changes**	**n**	**Not appropriate**	**n**	**Total**			
		1	Healthcare workers offer flexibility in visitation hours when my child needs intensive care. Response options: Never / Seldom / Sometimes / Usually / Always	66.67%	16	12.50%	3	20.83%	5	24	**ELIMINATE**	Change healthcare workers to institutions/facilities<br>Same explanation as above -- this is likely a reflection of institutional policies and not a reflection of the healthcare workers.<br>Healthcare facilities or workers offer....	Item eliminated as process is not within the control of HCWs.
		2	Healthcare workers offer flexibility in visitation hours when my child is admitted to the general ward. Response options: Never / Seldom / Sometimes / Usually / Always	54.17%	13	16.67%	4	29.17%	7	24	**ELIMINATE**	Not too sure about this - because I don't think most HCW have the authority to offer such flexibility (unlike when in ICU settings)<br>Have to follow to hospital guidelines for visitation hours. Especially in general ward when child is not in critical stage.<br>Same as above<br>Healthcare facilities or workers offer....<br>is it really the workers who offer flexibility?	Item eliminated as process is not within the control of HCWs.
		3	Healthcare workers offer flexibility in the number of caregivers allowed at bedside when my child admitted to the general ward. Response options: Never / Seldom / Sometimes / Usually / Always	50.00%	12	20.83%	5	29.17%	7	24	**ELIMINATE**	Not too sure about this - because I don't think most HCW have the authority to offer such flexibility (unlike when in ICU settings)<br>when my child is admitted<br>Have to follow to hospital guidelines for visitation hours. Especially in general ward when child is not in critical stage.<br>At times some healthcare worker ignored on some patient's visitors which is more that caregiver at the bed side and pretend as didnt see it as children and caregiver need some rest hope they could expedite visitors except for caregiver after visiting hours.<br>Same as above, and add "when needed"	Item eliminated as process is not within the control of HCWs.
		4	Healthcare workers refer me to specialists as needed. Response options: Never / Seldom / Sometimes / Usually / Always	75.00%	18	12.50%	3	12.50%	3	24	**[MOVE TO ACCESSIBLE MEDICAL CARE]** Healthcare workers refer ~~me~~ **my child** to **relevant** specialists as needed.	I think this is about accessibility<br>Not sure what this question mean. Refer caregivers to specialists for their own health conditions?<br>HCW dicusses with me and refers...<br>Need to be specific about which or what types of specialists. Eg. say relevant or appropriate?<br>This item, if retained, goes better with "Accessible Medical Care.	Most suggested changes incorporated.
			**Round 2 REVISED WORKING ITEM (PROCESSES)**	**Yes, appropriate**	**n**	**Not appropriate**	**n**	**Total**			**Round 3 REVISED WORKING ITEM (PROCESSES)**		
		1	Healthcare workers work as a team towards the same goals for my child's care. (unchanged)	100.00%	24	0.00%	0	24			Unchanged		
		2	I have a main healthcare worker who or team who consistently oversees my child's medical needs.	91.67%	22	8.33%	2	24			I have a ~~main~~ healthcare worker**/** ~~who or~~ team ~~who~~ **that** consistently oversees my child's medical needs.	I have a consistent healthcareworker or team overseeing my child's medical needs<br>main healthcare worker or team (typo)<br>Change to "I have a healthcare worker or team who consistently oversees my child's medical needs."<br>Remove first "who" in the sentence.<br>I have a healthcare worker or a healthcare team who consistently oversees my child's medical needs.	Most suggested changes incorporated.

Theme	Subtheme	No.	Item	%	n	%	n	%	n	Total	Round 3 revised working item	Comments	Response
	Coordination and continuity of care	3	I have a healthcare worker who or team which coordinates my child's care between different disciplines, services, and agencies.	87.50%	21	12.50%	3	24			I have a healthcare worker/ ~~who or~~ team ~~who~~ **that** coordinates my child's care between different disciplines.~~, services, and agencies.~~	healthcare worker or team who (typo)<br>I have a healthcare worker or a healthcare team which coordinates my child's care between different disciplines, services and agencies.<br>Need to differentiate this from above. Call the one above "healthcare professional". And here "a single healthcare worker who coordinates my child's care between..."<br>The wording is awkward. I assume the first "who" is a typo.<br>avoid double barrel	Most suggested changes incorporated.
		4	I receive consistent information from different healthcare workers.	100.00%	24	0.00%	0	24			Unchanged		
		5	Healthcare workers ensure a smooth transition of care across the different healthcare settings (e.g., NICU to PICU, Private to Public facilities, going home, hospital admission).	95.83%	23	4.17%	1	24			Healthcare workers ensure a smooth transition of care across the different healthcare settings (e.g., NICU to PICU, Private to Public facilities, going home, hospital **or hospice** admission).	Add hospice?<br>...transition of care for my child across...<br>too complex?	Retained as-is; care may extend beyond child's medical care to care structures as well.
		6	Healthcare workers coordinate my child's appointments to reduce our hospital visits.	95.83%	23	4.17%	1	24			Healthcare workers **organise** ~~coordinate~~ my child's appointments to reduce our hospital visits.	"organise" instead of coordinate (to keep distinct from above).<br>"health care workers' sound a little impersonal / abstract. would some type of qualifier like 'my', 'at x clinic' , etc make it all read better?	Most suggested changes incorporated.
			**[New items suggested in Round 1 for testing in Round 2]**	**Yes, appropriate**	**n**	**Yes, with changes**	**n**	**Not appropriate**	**n**	**Total**			
		1	Healthcare workers coordinate my child's care across healthcare and education sectors for my child's medical, developmental and education needs. Response options: Never / Seldom / Sometimes / Usually / Always	79.17%	19	8.33%	2	12.50%	3	24	**ELIMINATE (due to high overlap with the constructs captured by other indicators)**	This subtheme should be limited to a medical setting.<br>Overlap with previous question<br>This is a very complex statement and somewhat similar to the care coordination item "I have a healthcare worker or team who..." above. Perhaps "Healthcare workers ensure that my child's medical, developmental, and educational needs are met across settings.'<br>Most of the time they try to coordinate<br>Duplicate of above. We could add the need to coordinate with schools to above if indicated. "Agencies" was mentioned.	Eliminated due to high overlap with existing items.
		2	Healthcare workers make sure my child's medical equipment are properly maintained beyond healthcare facilities. Response options: Never / Seldom / Sometimes / Usually / Always	66.67%	16	12.50%	3	20.83%	5	24	**ELIMINATE**	Not sure what "beyond healthcare facilities" means<br>rather than "beyond" is it "outside" healthcare facilities?<br>Depend on the model of care, this may not be the responsibility of the healthcare worker. Some models are managed by outsourced third parties or vendors and are equally effective.<br>This is likely outside of the scope of most healthcare teams<br>Depends the medical equipment where the family is getting from	Eliminated due to concerns over relevance to HCWs and problems with wider applicability.
			**Round 2 REVISED WORKING ITEM (PROCESSES)**	**Yes, appropriate**	**n**	**Not appropriate**	**n**	**Total**			**Round 3 REVISED WORKING ITEM (PROCESSES)**		
Professional Qualities of HCWs	Responsive and sensitive communication	1	Healthcare workers present themselves in an honest way	91.67%	22	8.33%	2	24			Healthcare workers present themselves in an **authentic** ~~honest~~ way.	Perhaps use "Authentic" rather than honest<br>Healthcare workers present themselves in an honest and non-judgmental way of	Changes made.
		2	Healthcare workers make an effort to build a trusting relationship with me.	100.00%	24	0.00%	0	24			Unchanged		
		3	Healthcare workers take responsibility for my child's wellbeing when he/she is under their care.	95.83%	23	4.17%	1	24			**[MOVE TO COMPETENCY OF HEALTHCARE DELIVERY]**	Not really a "communication" response.	Changes made.
		4	Healthcare workers support my right to information about my child by ensuring I am always fully informed.	100.00%	24	0.00%	0	24			Unchanged	May be difficult for layperson to understand the sentence. Suggest rephrase "Healthcare workers ensure that I am informed or have access to information about my child in a timely manner"<br>This is also a complex statement. Perhaps "Healthcare workers ensure that I am fully informed about my child's care."	No changes made in light of majority vote; most panelists found item appropriate.
		5	Healthcare workers give me information on my child's condition in a timely manner.	95.83%	23	4.17%	1	24			Unchanged		No changes made in light of majority vote; most panelists found item appropriate.
		6	Healthcare workers communicate with me in a way that is sensitive to my needs.	100.00%	24	0.00%	0	24			Unchanged		
		7	(unchanged) Healthcare workers communicate with me in a way that I can understand.	100.00%	24	0.00%	0	24			Unchanged		
		8	(unchanged)Healthcare workers give me enough time to think about decisions for my child's care.	100.00%	24	0.00%	0	24			Unchanged		
		9	Healthcare workers have an appropriate sense of urgency when communicating with me so that additional stress is not created (e.g. not calling multiple times throughout the day for non-urgent issues, informing me about critical news quickly).	83.33%	20	16.67%	4	24			Unchanged	At times some healthcare worker didn't read up the case sheets that is sated in the system especially for the child who is a frequent admission. Some will just woke u up at 3am in the morning to ask a non urgent matter and that makes an anxiety to a parents with a complex care as thinking that the child is in critical for waking up that time. It would be appropriate to read up and not keeping asking the same repeating question<br>"in a way that is sensitive to my needs" should cover this aspect<br>This is a complex statement that I feel is covered by other items.<br>ok item, but too complex wording	No changes made in light of majority vote; most panelists found item appropriate.
		10	(unchanged) Healthcare workers show me genuine care and sincerity.	95.83%	23	4.17%	1	24			[**MOVE TO PROVIDING PSYCHOSOCIAL SUPPORT FOR PARENTS AND FAMILY**] Healthcare workers show me genuine care and ~~sincerity~~ **concern**.	Suggest "genuine care and concern". genuine should be sincere.<br>I don't think that sincerity a requirement for "responsive and sensitive" communication.<br>'show me care"?	Most suggested changes incorporated.
		11	(unchanged) Healthcare workers are respectful of my spiritual or religious customs and beliefs.	100.00%	24	0.00%	0	24			Unchanged		
			**[New items suggested in Round 1 for testing in Round 2]**	**Yes, appropriate**	**n**	**Yes, with changes**	**n**	**Not appropriate**	**n**	**Total**			
		1	Healthcare workers are sensitive to the cultural issues that may affect my role or ability to care for my child. Response options: Strongly Disagree / Disagree / Neither Agree nor Disagree / Agree / Strongly Agree	79.17%	19	8.33%	2	12.50%	3	24	**ELIMINATE (due to high overlap with the constructs captured by other indicators)**	I feel this is covered under "Healthcare workers are respectful of my spiritual or religious customs and beliefs".<br>Difficult to understand. May invite more questions from the responder.<br>Can give examples? Not very clear on issues<br>Duplicate<br>delete "the" before cultural	Eliminated due to high overlap with existing items.
		2	Healthcare workers communicate with my child in a way that is sensitive to his/her needs. Response options: Never / Seldom / Sometimes / Usually / Always	95.83%	23	4.17%	1	0.00%	0	24	Unchanged	Healthcare workers communicate or care for my child in a way that is sensitive to his/her needs	Care not added to focus on subtheme of communication.
			**Round 2 REVISED WORKING ITEM (PROCESSES)**	**Yes, appropriate**	**n**	**Not appropriate**	**n**	**Total**			**Round 3 REVISED WORKING ITEM (PROCESSES)**		
	Competency of Healthcare delivery	1	Healthcare workers present their knowledge, skills and experience so that I am confident they can meet my child's needs	87.50%	21	12.50%	3	24			Healthcare workers **assure me of** ~~present~~ their **capabilities** ~~knowledge, skills and experience~~ so that I am confident that they can meet my child's needs.	Vague. "Present their knowledge" but how?<br>I am not able to get at what the item is trying to ask	Changes made for clarity.
		2	Healthcare workers avoid unnecessary treatments and investigations on my child. Response options: Strongly Disagree / Disagree / Neither	87.50%	21	12.50%	3	24			Healthcare workers avoid ~~unnecessary~~ treatments and investigations ~~on my child~~ **that are not aligned with my goals for my child's care**.	Does not seem to fit with the other items but not sure where it goes.<br>I think this statement is a bit vague. Perhaps "Healthcare workers only provide	Most suggested changes incorporated.
		3	Healthcare workers provide medical care for my child without undue delay.	87.50%	21	12.50%	3	24			Healthcare workers provide medical care for my child **in a timely manner** ~~without undue delay~~.	Is this more about accessibility?<br>Perhaps "...in a timely manner" rather than "without undue delay"?<br>Sounds more like efficiency<br>is this a repeat from page 1	Most suggested changes incorporated.
		4	Healthcare workers do all they can to identify and treat my child's medical issues.	91.67%	22	8.33%	2	24			Healthcare workers do all they can to identify and ~~treat~~ **manage** my child's medical issues.	"do all they can" and "are able to" identify and treat has a subtle, but i feel important difference. I feel the latter might be more appropriate.<br>This is also subject to interpretation. In a palliative care setting, we may not want to identify and "treat" all medical issues but manage symptoms<br>double barrel	Not changed to "are able to" due to feedback froun Round 1 in which majority of experts suggested softer language.
		5	(unchanged) Healthcare workers manage my child's physical symptoms to make sure my child is comfortable.	100.00%	24	0.00%	0	24			Healthcare workers manage my child's physical symptoms to make sure my child is comfortable **and pain-free**.	perhaps add "comfortable and pain-free"<br>Why is there only an item on physical symptoms? What about psychosocial or spiritual?<br>is this 'competency'?	Pain-free' added. Other domains of child's well-being beyond physical are captured in other subthemes.
			**Round 2 REVISED WORKING ITEM (PROCESSES)**	**Yes, appropriate**	**n**	**Not appropriate**	**n**	**Total**			**Round 3 REVISED WORKING ITEM (PROCESSES)**		
		1	Healthcare workers tell me what to look out for so that I know what to do when my child he/she is unwell.	87.50%	21	12.50%	3	24			Healthcare workers tell me what to look out for so that I know ~~what to do~~ when my child ~~he/she~~ is unwell.	Suggest "Healthcare workers tell me what to look out for so that I know when my child is unwell" If not, it will be an overlap with the next question.<br>Feels similar to the item below	Most suggested changes incorporated.
		2	(unchanged) Healthcare workers equip me with skills so that I can confidently care for my child out of the hospital.	100.00%	24	0.00%	0	24			Unchanged		

Supporting parent caregivers	Empowering parent-caregivers	3	(unchanged) Healthcare workers acknowledge and affirm my efforts in caring for my child.	100.00%	24	0.00%	0	24			Unchanged		
		4	(unchanged) Healthcare workers give me opportunities to bond with my child during hospital or hospice admissions.	95.83%	23	4.17%	1	24			Healthcare workers give me opportunities to bond with my child during hospital or hospice admissions **e.g. through my child's daily care**.	"...give me opportunities to bond" feels vague to me.<br>double barrel	Example provided for clarity.
		5	Healthcare workers make home visits to support my caregiving by checking that my child is well taken care of at home. Response options: Never / Seldom / Sometimes / Usually / Always / Not applicable to my child	91.67%	22	8.33%	2	24			Healthcare workers make home visits to support **the care of my child at home**~~my caregiving by checking that my child is well taken care of at home~~.	Awkward wording at the end. Sound like checking up on the parent which is not empowering. I would leave it as ... support my caregiving.<br>The responses are confusing. What does "always" "usually" , etc mean? Some may also take it to mean home nursing.<br>Yes, but also very complex as worded. How about "Healthcare workers make home visits to support the care of my child at home"<br>Need to specify which HCW and which types of patients you are referring to.	Most suggested changes made for clarity.
		6	(unchanged) Healthcare workers give me opportunities to advocate or speak up for my child and myself.	100.00%	24	0.00%	0	24			Healthcare workers give me opportunities to advocate or speak up for my child ~~and myself~~.	double barrel	Change has been made to avoid double-barrel indicator.
		7	Healthcare workers provide me with opportunities to give back to the special needs community when I approach them e.g. letting me support other families, participate in research and raising funds. Response options: Never / Seldom / Sometimes / Usually / Always / I prefer not to be involved	100.00%	24	0.00%	0	24			Unchanged	complex wording	No changes made in light of majority vote; most panelists found item appropriate.
			**[New items suggested in Round 1 for testing in Round 2]**	**Yes, appropriate**	**n**	**Yes, with changes**	**n**	**Not appropriate**	**n**	**Total**			
		1	Healthcare workers provide teleconsultations so that I can confidently make adjustments to my child's medical needs at home e.g. medicine dosage, ventilator settings etc. Response options: Never / Seldom / Sometimes / Usually / Always / Not applicable to my child	87.50%	21	8.33%	2	4.17%	1	24	Healthcare workers provide **remote** consultations so that I can ~~confidently~~ **care for my child at home** ~~make adjustments to my child's medical needs at home e.g. medicine dosage, ventilator settings etc~~.	This overlaps with the responses on accessibility to care. Teleconsult is only a mode of obtaining support.<br>teleconsulations - do you mean just telephone calls, video calls or both	"remote" for clarity; indicator retained as a specific process to enable parent-caregivers' home-caregiving
	Providing psychosocial support to parents and family		**Round 2 REVISED WORKING ITEM (PROCESSES)**	**Yes, appropriate**	**n**	**Not appropriate**	**n**	**Total**			**Round 3 REVISED WORKING ITEM (PROCESSES)**		
		1	(unchanged) Healthcare workers support my hopes for my child.	100.00%	24	0.00%	0	24			Unchanged	what does it mean to support hope	No changes made in light of majority vote; most panelists found item appropriate.
		2	(unchanged) Healthcare workers prepare me for what may lie ahead.	100.00%	24	0.00%	0	24			Unchanged		
		3	(unchanged) Healthcare workers provide me with a compassionate listening ear.	100.00%	24	0.00%	0	24			Unchanged	a little too informal	No changes made in light of majority vote; most panelists found item appropriate.
		4	Healthcare workers give me appropriate time and space if I wish to be alone after receiving difficult news about my child.	100.00%	24	0.00%	0	24			Unchanged		
		5	Healthcare workers facilitate my access to parent support networks.	100.00%	24	0.00%	0	24			Healthcare workers facilitate my access to **available** parent support networks.	"to available parent support networks" these networks may not always be available<br>Social supports network? Not all are parents to the child. What about practical /resources supports ? eg home respite services, daycare, services	Most suggested changes made for clarity. "Parents" retained as this is currently designed as a parent-reported measure.
		6	Healthcare workers assess and where possible help with our family's psychosocial needs resulting from my child's condition.	100.00%	24	0.00%	0	24			Unchanged		
			**[New items suggested in Round 1 for testing in Round 2]**	**Yes, appropriate**	**n**	**Yes, with changes**	**n**	**Not appropriate**	**n**	**Total**			
		1	Healthcare workers refer me to professional psychosocial support if I should need it for myself.	91.67%	22	4.17%	1	4.17%	1	24	Unchanged	perhaps add: "and other family members<br>The response above should cover. Layperson would consider all healthcare workers "professionals"	No changes made in light of majority vote; most panelists found item appropriate.
	Reducing caregiving stress and burdens		**Round 2 REVISED WORKING ITEM (PROCESSES)**	**Yes, appropriate**	**n**	**Not appropriate**	**n**	**Total**			**Round 3 REVISED WORKING ITEM (PROCESSES)**	I have access to someone skilled to take care of my child when I need a break.	
		1	Healthcare workers help me find options for someone skilled to take care of my child so that I can take a break if I need it.	91.67%	22	8.33%	2	24			Unchanged		
		2	Healthcare workers give me practical suggestions on how I can reduce my child's medical costs.	95.83%	23	4.17%	1	24			Unchanged		
		3	Healthcare workers guide me to available resources to reduce my family's financial burden	100.00%	24	0.00%	0	24			Unchanged	overlaps with the one just before	No changes made in light of majority vote; most panelists found item appropriate.
		4	Healthcare workers do their best to avoid my child's unnecessary and unplanned hospitalization.	95.83%	23	4.17%	1	24			Healthcare workers do their best to **help us** avoid my child's unnecessary ~~and unplanned~~ hospitalization.	Suggest "Healthcare workers do their best to prevent unnecessary and unplanned hospitalization for my child"<br>Minor wording suggestion: "Healthcare workers do their best to help us avoid unnecessary hospitalizations for my child."<br>loaded question?	Most suggested changes made for clarity.
		5	Healthcare workers offer information for us to find specialized transport for my child who has mobility needs.	95.83%	23	4.17%	1	24			Healthcare workers offer information **on** ~~for us to find~~ specialized transport for my child who has mobility needs.	...workers help us in finding specilaized...<br>Reword	Most suggested changes made for clarity.
			**[New items suggested in Round 1 for testing in Round 2]**	**Yes, appropriate**	**n**	**Yes, with changes**	**n**	**Not appropriate**	**n**	**Total**			
		1	I am given sufficient time to find and train a long-term caregiver or domestic helper before my child leaves healthcare facilities.	95.83%	23	0.00%	0	4.17%	1	24	Unchanged	I think this is complicated, as it could be dependent upon the facility, the insurance/payor, or the availability of long-term caregivers in the home setting.	No changes made in light of majority vote; most panelists found item appropriate.
	Holistic approach to care for the child		**Round 2 REVISED WORKING ITEM (PROCESSES)**	**Yes, appropriate**	**n**	**Not appropriate**	**n**	**Total**			**Round 3 REVISED WORKING ITEM (PROCESSES)**		
		1	(unchanged) Healthcare workers put in effort to foster a personal relationship with my child.	100.00%	24	0.00%	0	24			Unchanged		
		2	Healthcare workers provide us with appropriate allied health support (e.g. therapists) to meet my goals for my child's development.	91.67%	22	8.33%	2	24			~~Healthcare workers provide us with~~ **I receive** appropriate allied health support (e.g., therapists) to meet my goals for my child's development.	therapists are healthcare workers. And therapy may be provided in the community.<br>To meet my goals is quite different from discussed/ set / or shared goals some goals may or may not be "realistic". Question may not capture this.	Shared goal-setting is captured in other indicators; current indicator specifically focuses on provision of allied health support that parents feel is sufficient for their set-goals.
		3	Healthcare workers do their best to create a child-friendly atmosphere in hospital or hospice.	91.67%	22	8.33%	2	24			Healthcare workers do their best to create a child-friendly atmosphere in **the** hospital or hospice.	This is assuming it is an adult hospital or hospice?<br>the hosipital or hospice	Both children's and adult hospitals and hospices can be sterile and/or non-stimulating for the child.
		4	Healthcare workers provide my child with emotional support.	100.00%	24	0.00%	0	24			Unchanged		
		5	Healthcare workers facilitate my child's access to appropriate facilities or services for play and engagement.	95.83%	23	4.17%	1	24			Unchanged	In the hospital? If in the community, the coordination of care section has taken care of that.	No changes made in light of majority vote; most panelists found item appropriate.
		6	Healthcare workers facilitate my child's access to special needs schools when I ask. Response options: Yes / No / Not applicable to my child	91.67%	22	8.33%	2	24			Healthcare workers facilitate my child's access to special needs schools when I ask. Response options: Yes / No / **My child does not need special needs school**	Suggest change "when I ask" to "when needed". The child may not need special needs schools and healthcare workers would not facilitate that.<br>The question below might be better	Modified response options to allow parents to state whether child needs special needs school.
			**[New items suggested in Round 1 for testing in Round 2]**	**Yes, appropriate**	**n**	**Yes, with changes**	**n**	**Not appropriate**	**n**	**Total**			
		1	[If child attends special needs school] Healthcare workers engage my child's school on my child's medical needs in school. Response options:	83.33%	20	16.67%	4	0.00%	0	24	[If child attends ~~special needs~~ school] Healthcare workers engage my child's school **to support his/her** ~~on my child's~~ medical needs **while** in school. Response options: Never / Seldom /	Not all children with serious illness attend special needs schools. Perhaps [if child attends school] Healthcare workers work with my child's school to support	Most suggested changes made for clarity.
		2	Healthcare workers assess my child's developmental milestones and progression. Response options: Never / Seldom / Sometimes / Usually / Always	91.67%	22	8.33%	2	0.00%	0	24	Healthcare workers assess my child's developmental milestones and progress~~ion~~ **where appropriate.**	Healthcare workers assess my child's development needs<br>Minor wording suggestion: "...milestones and progress, if appropriate."	Most suggested changes made for clarity.
		3	I receive adequate financial support for my child's non-medical expenses e.g., special needs education, therapy, etc. Response options: Yes / No	66.67%	16	12.50%	3	20.83%	5	24	**[MOVE TO REDUCING BURDEN]** I receive ~~adequate~~ financial **assistance** ~~support~~ for my child's non-medical expenses **if needed** e.g., special needs education, therapy, etc.	Additional indicator of 'not fully supported'<br>I think this fits better under the sub theme on reducing burden<br>May not receive if there is no need. Suggest "have access if needed"	Most suggested changes made.
Collaborative and holistic care			**Round 2 REVISED WORKING ITEMS (PROCESSES)**	**Yes, appropriate**	**n**	**Not appropriate**	**n**	**Total**			**Round 3 REVISED WORKING ITEM (PROCESSES)**		
		1	(unchanged) Healthcare workers give me complete information on all management options for my child so that I can make informed decisions.	100.00%	24	0.00%	0	24			Healthcare workers give me ~~complete information on~~ all **possible** management options for my child ~~so that I can make informed decisions~~.		No expert comments; however, upon considering comments in the below indicator, facilitators clarified the items more explicitly.
		2	Healthcare workers clearly explain the pros and cons of different medical technologies and procedures on my child (e.g. Continuous positive airway pressure (CPAP), tracheostomy) so that I am aware of the effects it will have on my child's daily life.	87.50%	21	12.50%	3	24			Healthcare workers clearly explain the pros and cons of **all management options for my child** ~~different medical technologies and procedures on my child~~ so that I **can make informed decisions.** ~~am aware of the effects~~ **they** ~~it will have on my child's daily life e.g. Continuous positive airway pressure (CPAP), tracheostomy,~~ **tube-feeding**~~.~~	Overlap with above question<br>To include feeding care etc as not all kids are on trach or NIVs<br>...effects they will...<br>ok, but too complex wording	This indicator focuses on communicating all possible effects of various management options to parents to enable informed decision-making.
		3	A palliative care team or specialist(s) participates in my child's treatment plan.	83.33%	20	16.67%	4	24			**[MOVE TO HOLISTIC APPROACH TO CARE FOR CHILD]**<br>**I am able to discuss my child's treatment plan with a palliative care team or specialist(s) if I want to.**	Not sure how this relates to decision-making. Maybe it fits under accessibility<br>Why is the purpose of this response? Not all children require palliative care<br>Need to explain "palliative care"?	Indicator has been reworded and shifted to another subtheme. We are retaining the indicator given that all children falling into the spectrum of serious illness categories "would benefit from some elements of the palliative care approach and from knowing about the support that is available from children's palliative care services" (https://www.togetherforshortlives.org.uk/changing-lives/supporting-care-professionals/introduction-childrens-palliative-care/categories-of-life-limiting-conditions/).

	**Shared decision-making**	4	[If parent responds yes to prior item] Healthcare workers introduced the palliative care team or specialist(s) at an appropriate time. Response options: Yes / No	87.50%	21	12.50%	3	24			**[MOVE TO HOLISTIC APPROACH TO CARE FOR CHILD]**<br>[If parent responds yes to prior item] Healthcare workers introduced the palliative care team or specialist(s) at an appropriate time. **Response options: Too late / Late / Neither late nor early / Early / Too early**	Accessibility<br>Suggest response be likert format: e.g. Too late/ Late/ Neither late or early/ Early / Too early<br>Not appropriate but "early".<br>why change to past tense here?	Response options changed. Indicator has shifted to another subtheme. Past tense as this is one of the few indicators that would have already happened at a single time point in the past (if parent had responded yes to the earlier indicator)
		5	Healthcare workers discussed how the scope of care can be tailored to provide comfort for my child when I was given a poor prognosis of my child's condition.	87.50%	21	12.50%	3	24			When I was given **the** ~~a poor~~ prognosis of my child's condition, **healthcare workers discussed with me how the scope of care can be tailored to provide comfort for my child**.	Some overlap with first question<br>This is a very complex sentence and for some parents, the poor prognosis might have been given months or years ago.<br>Suggest to remove the " poor" ....instead ....." was given the news on the prognosis of my child's condition." It may not matter how poor the prognosis be if question is used for all cases with varying prognosis looking at shared decision making.<br>When I was given a poor....condition, healthcare workers discussed how care can be tailored to provide comfort for my child with me<br>complex logic	Most suggested changes made for clarity.
		6	Healthcare workers listen to my inputs regarding my child's care.	100.00%	24	0.00%	0	24			Unchanged	Overlap with previous response on listening	No changes made in light of majority vote; most panelists found item appropriate.
		7	I am as involved as much as I wish to be in decision-making about my child's treatment.	91.67%	22	8.33%	2	24			I am as involved as much as I wish to be in decision-making about my child's **care** ~~treatment~~.<br>**Response options: Strongly agree / Agree / Neither agree nor disagree / Disagree / Strongly disagree**	Suggest : I am involved in the decision-making about the care plan for my child<br>I am involved..	As much as I wish to be' deemed an important component to retain as parents have varying levels of preference for involvement.
		8	Healthcare workers provide care to my child while considering my family's preferences for treatments.	91.67%	22	8.33%	2	24			Healthcare workers consider~~ing~~ my family's preferences for treatments **when** provide~~ing~~ care to my child.	Healthcare workers consider my family's preferences for treatment, when providing care to my child.<br>Overlap with above<br>This is vague. While considering or "according to ..."	Most suggested changes made for clarity.
