# Supplementary material for: Development of the Parental Experience with Care for Children with Serious Illnesses (PRECIOUS) quality of care measure
Source: BMC Palliat Care. 2024 Mar 8;23:66. doi: 10.1186/s12904-024-01401-x (PMC10921687; doi:10.1186/s12904-024-01401-x)
Supplement: Supplementary file 3 — Additional file 3. [file 12904_2024_1401_MOESM3_ESM.pdf]

Additional file 3. Pre-testing results

Batch 1
Italicized font and strikethrough: Suggested revisions after steering committee input

	Over the past 6 months, we…	**P6 (70 mins) transcript summary, Category 4 illness. 2.5 year old female.**	**P3 (115 mins) transcript summary, Category 3 illness. 6.5 year old male.**	**P9 (52 mins) transcript summary, Category 3 illness. 5.5 year old male**	**P8 (35 mins) transcript summary, Category 1 illness. 8.5 year old male.**	**Facilitator comments**	**Steering committee comments**	**Steering committee comments (cont.)**
1	… had access to a range of medical exper*tise* needed to manage my child's condition(s).	When you talk about 'access'… does it mean home care or medical experts that if I need to I can email, for e.g. her [specialists]? Since we're in home care prog I would think yes, ready access. We also have WhatsApp, which I use a lot to get in touch with them.	When I read this… I don't know, I feel there is no 'expert' here for his condition. They only treat whatever that comes up. So it's a simple statement but it opens a pandora's box because all of them don't have any answers and no reference cases.	No issues w indicator	Multi-D team, able to have diff medical experts from diff disciplines. But 'range' is a bit tricky… I would also think is it private or public hospital? So I think a whole multi-d team.	This indicator should be discussed. Is it range of medical experts or range of medical expertise? Is it about access to the advice/medical input?	I like better access to range of medical expertise	… had access to THE medical expertise needed to manage my child's condition(s).
2	… received guidance on how to obtain my child's medical equipment*(s)* and supplies *e.g., medications, medical consumables.*	No issues w indicator	No issues w indicator	No issues w indicator	Didn't know that medications or nursing kits are supplies. 'How to obtain'... Cos always prescribed. Should change to receive, not obtain.	Add examples but leave it as obtain.	obtain better than receive	

3	… had access to *sufficient* financial support for my child's medical expenses.	Not clear what is the source of financial support; what about my family members?	The question itself is easy to understand. But I don't qualify for most of the support. So no.	No issues w indicator; we barely received any support because not eligible.	Not many parents have any financial support… maybe financial assessment or counselling. Everyone needs financial counselling but I don't need financial support.	Focus is on whether families perceive they received 'fair', 'means-tested' support. The perception of 'sufficiency' is more important than absolutely having received or not.	I don't understand the assessment - just having an assessment does not mean you can afford anything	I LIKE THE CHANGE
4	… had a healthcare worker/team that supervised *the management of* my child's medical needs.	Thinks about Home Care team	What does it mean to 'supervise'? Just outpatient follow ups? Is that supervision already? Because when I see supervise I think it should be more daily, but that is all on us. For this and 5 I answer in the context of [Home Hospice].	No issues w indicator	Quite clear. Not sure if across or within teams.	Is 'oversee' less confusing that 'supervise'? Diff between 4 and 5 is that 4 is whether there's a HCW who is familiar with child's complexities. 5 is about coordinating across teams.	I thought they actually provided the care rather than just oversaw it	I LIKE THE CHANGE
5	… had a healthcare worker/team that coordinated my child's care across different care services.	Thinks about Home Care team	No issues w indicator. There is no real coordination. Unless we **ask** for it, e.g. recently we got Covid and child needed to be protected, [Home Hospice] did coordinate, so sometimes.	No issues w indicator. Non-resourceful people cannot handle; it wasn't coordinated at all, my wife is the one who sought everyone out.	No issues w indicator. This is clearly lacking in health system			

6	… received consistent information from different healthcare workers.	No issues w indicator	No. The only consistent info is the 'everything is because of his underlying condition', frustrating.	No issues w indicator	No issue with indicator. Need to seek second opinion, need to coordinate and analyze everything.			
7	… had access to *sufficient* financial support for my child's non-medical expenses e.g., special needs education, ~~*physiotherapy, occupational therapy,*~~ speech therapy etc.	Again, what's the source of financial support? The frequency-based response option is not appropriate. Should be a % e.g. how many % of my child's non-medical expenses are subsidized or covered	Other than [government early intervention prog], the MSW told us to apply for XXX but I don't qualify for anything. I had to jump all the loops just to get some [govt. programme] subsidy. And even then [the govt prog] is only for those 6 and below so he cannot even attend anymore.	No issues w indicator; we barely received any support.	I thought is financial assessment or financial counselling.	Focus is on whether families perceive they received 'fair', 'means-tested' support. The perception of 'sufficiency' is more important than absolutely having received or not.	here also I don't get the ASSESSMENT part.	I LIKE THE WORDING BUT WOULD ARGUE THAT PHYSIOTHERAPY IS MEDICAL, NOT NON-MEDICAL
8	… received appropriate allied health support (e.g., physiotherapists, speech therapists) to meet our goals for my child's development.	Thinks about [govt early intervention program], therapists. No issues	No issues w indicator. The [govt. early intervention] programme didn't work. It is so infrequent and he gets barely any attention, he lay on the floor alone. It cost 700 per month, we then changed to another [private] programme, which was slightly cheaper but too far away. All options are either not accessible, or not appropriate!	No issues w indicator	No issues w indicator			
~~9~~	*ADD SCREENING QUESTION: Is your child receiving any advice or care from a palliative or supportive care team or*	Not applicable to my child, only for terminally ill patients	[Home Hospice]… other than them, hardly able to discuss with any others. Thinks hospitals in SG have no pall team or pall care.	We did but how do I say… they are too enthusiastic, they discuss with us too many times. Not a comfortable convo to have. Opposite problem.	My child doesn't need	Add screening question prior to 10, and remove 9?	either 9 or 10 are fine but don't ask at all if child's conditional is not terminal	I DON'T THINK ALL WILL KNOW THE WORD 'PALLIATIVE' OR WHAT IT REFERS TO.

	specialist? YES / NO<br>*Description: *The service provides a holistic care approach via the multidisciplinary team that encompasses psychological, emotional and spiritual support. Supportive care and palliative medicine is appropriate at any age and at any stage in a serious illness, and it can be provided along with curative treatment.*<br>~~*… were able to discuss my child's treatment plan with a palliative care team or palliative care specialist(s) if we wanted to.*~~							

~~10~~	*… were introduced to a palliative care team or palliative care specialist (s) at an appropriate time.*	Not applicable to my child, only for terminally ill patients	Too late. When first diagnosed, no one told us about pall care. We only found out about [Home Hospice] when I went to seek a second opinion from someone friends recommended. We didn't want to engage [Home Hospice] then because I thought kids go there to die. We didn't want that. We dug our heels in. But ever since having [Home Hospice], we actually feel more at peace, always. There needs to be more awareness. Hospitals need to take a proactive approach.	No issues w indicator	My child doesn't need			MAYBE USE THE TERM PALLIATIVE AND SUPPORTIVE CARE. AND PERHAPS DEFINE WHAT YOU MEAN.
**Over the past 6 months, my child's healthcare workers…**								
11	…were approachable when we needed advice about my child's care .	I would say yes, my wife would say no. So I think sometimes.	Discussed negative experience about HOD being insensitive to mother's religious beliefs. Filed a complaint to hospital and received unsatisfactory response, no follow-up. 3rd complaint filed already. Incidence gave mother panic attacks and created awkwardness.	I think about it when he's hospitalized, what's the advice they give, or homecare, in general is the HCP able to work with us? They do what they can. If cannot we will go [to the hospital].	No issues w indicator			
12	…worked as a team towards common goals for my child's care.	No issues w indicator	I think about it during hospitalizations.	No issues w indicator	No issues w indicator			

13	…organized my child's appointments to reduce our hospital visits.	No issues w indicator	No issues w indicator. Departments did not communicate at all, and we were going to hospital every 2 weeks. I don't even know how we did it.	No issues w indicator	No issues w indicator			
14	…put in effort to build a trusting relationship with us.	No issues w indicator	No issues w indicator. Very transactional. They only focus on medical caregiving. Only one doctor that has journeyed with us from the start went out of his way, stayed longer hours, talked to us, built that trust.	No issues w indicator	No issues w indicator			
15	…kept us ~~*fully*~~ *well* informed about my child’s condition.	This is very subjective, 'fully' makes me uncomfortable because I don't know what I don’t know.	No issues w indicator	No issues w indicator	No issues w indicator	Does replacing 'fully' with 'well' reduce some of that absoluteness which P3 is not comfortable with?	well is better than fully	
16	…communicated with us in a way that was sensitive to our needs.	No issues w indicator	No issues w indicator	No issues w indicator	Don't understand this question. I thought emotional needs. So shd clarify that it's emotional needs.			
17	…gave us enough time to think about decisions for my child's care.	No issues w indicator	No issues w indicator	No issues w indicator	No issue with indicator			

18	~~…did not bother us unless it was something important.~~	I assume they never bother us UNLESS it's important	No issues w indicator	I'm sure that the info they want to give, be in in or out of hospital, they will inform us. Maybe info did not need to inform immediately, but eventually it will flow down. The personnel has the responsibility to relay info, but now or a few hours later? 'Bother' is not that much lah. They have to uphold. I don't think there's a good way to ask this cos all parents are too different.	This question sounds very negative. Not sure what qn is trying to achieve. Not sure how to decide if impt or not but even if impt, I also want to get involved. Sounds like meant to be a postive qn, but came across negative. Maybe 'did not stress us'?	Indicator is captured by 16. Suggest to remove since parents struggle with it.	ok	
19	…communicated with my child in a way that was sensitive to his/her needs.	Not Applicable because my child is too young	Not applicable because he is non-verbal, mostly non-responsive.	No issues w indicator	Emotional or physical or what needs? Or biological needs? Shd be holistic. Nursing- biological. Social worker- emotional. Elicit each domain separately.	P8 struggled but the rest did not raise the same concern about wanting to divide into domains. Perhaps keep testing as-is.		
20	…did all they could to identify and manage my child's medical issues.	No issues w indicator	No issues w indicator	No issues w indicator	No issue with indicator			
21	…treated my child in a timely manner.	This question… I stumble. Because I HAVE to trust them; my response is not an absolute one but a subjective one, but I have to give them benefit of doubt.	No issues w indicator	No issues w indicator	No issue with indicator			

22	…avoided treatments and investigations that were not aligned with our goals for my child’s care.	No issues w indicator… sometimes they do things that retrospectively we think… hmm… did you really need to?	No issues w indicator	Misunderstood the statement, thinks its abt HCWs having to follow parents and cannot avoid treatments	No issue with indicator			
23	…managed my child's physical symptoms to make sure he/she was comfortable.	Especially in ICU, I feel this is subjective. The staff prioritize the condition over her 'comfort' and I have to go with that.	No issues w indicator	No issues w indicator	Stumbled on physical symptoms. Not sure if it meant fever or pain. Sounds like movement and exercise and pain. Should call it physiological symptoms, like fever, nausea, vomiting.	To address P6 and P8, Replace 'physical symptoms' with 'pain and discomfort'?		
24	…took responsibility for my child's wellbeing when he/she was under their care.	I feel item 21-24 are all about trust- I cannot answer them objectively but I assume always because I trust them.	No issues w indicator	No issues w indicator	No issues w indicator			
25	…told us what to look out for so that we knew when my child was unwell.	No issues w indicator	No issues w indicator	No issues w indicator	No issues w indicator			
26	…equipped us with skills so that we could confidently care for my child.	No issues w indicator	No issues w indicator.	No issues w indicator	No issues w indicator			
27	…acknowledged and affirmed our efforts in caring for my child.	No issues w indicator	Again, I have to respond in the context of SP. Hospital is very transactional, except one doctor.	No issues w indicator	No issues w indicator			

28	…~~gave us opportunities to~~ *listened to us when we* advocate*d* or ~~speak~~ *spoke* up for my child.	No issues w indicator	In what context? Because we do it, whether they give us the choice or not. Also, I think it's more about when he is hospitalized and decisions have to be made urgently, like patient might be in pain- not so much to outpatients.	This item is odd because I will speak up regardless. It's more abt listening or not.	No issues w indicator	Suggest to address P3 and P9's concern by replacing 'opportunities' with listening to the input, thus combining indicator 42.	the opportuniti es bit is not as good as just listen	
29	…showed us ~~genuine~~ care and concern.	I would remove the word 'genuine', I find that really uncomfortable because to be honest- I don't know. They do show care and concern but sometimes I feel it's just because it's their job and they have to. Is it genuine though? I doubt so.	Again, I respond thinking about SP. Definitely not the hospital. So… 'usually'.	No issues w indicator	No issue with indicator. Similar to 32,33. Can consolidate?		agree - take out genuine	
30	…supported our hopes for my child.	No issues w indicator	No issues w indicator	No issues w indicator	This is tricky. What is hope? I think everyone hopes their child will recover but is it fully? Shd be supported our goals or our decisions. Because hope of full recovery can be unrealistic, and if its unrealistic, how can HCPs support our so called false hope? Need to pain a realistic and reasonable picture, like support my goals.	Abstract for some, perhaps necessarily so- but perhaps acceptable for others. Keep testing.	agree that this one is quite abstract	

31	…prepared us for what may lie ahead.	No issues w indicator	I think you need to add 'try to'. They simply are not trained to do this, and they will never be able to prepare us for a journey they will never and are not walking. They try to be diplomatic- but that's all they can do.	No issues w indicator	No issue with indicator			
32	…provided us with a compassionate listening ear.	No issues w indicator	No issues w indicator	No issues w indicator	No issue with indicator but similar to 29			
~~33~~	~~*…gave us appropriate time and space if we wished to be alone after receiving difficult news about*~~ *my child.*	This I think is already captured by 16 and 17. And I also need to pause and think about WHEN we actually needed this time and space… I don't think we actually ever needed it so this would be Not Applicable to me.	No issues w indicator	No issues w indicator	No issue with indicator but similar to others	To address P6 and P8, suggest removing indicator	ok	
34	…facilitated *our* access to available parent support networks.	Change the frequency-based response to Yes or No.	No issues w indicator	He misunderstands statement and thinks about coordination, supporting parents at home rather than parent-support groups	No issues w indicator			
35	…guided us on how to reduce my child's medical costs.	I kind of feel that this is not the role of HCWs sometimes if we are using equipment that are not hospital-acquired.	No issues w indicator	No issues w indicator	No issues w indicator			

36	… fostered ~~a~~ personal*ized interactions* ~~*relationship*~~ with my child.	This also makes me uncomfortable. Was it just professional or more, actually personal? I think it should be rephrased to "went above and beyond their professional capacity"	Hospital confirm no. SP they still text us regularly about him even when he's hospitalized and they are not seeing him.	No issues w indicator	Personal r/s meaning what? Sounds like extending beyond doctor-patient r/s. Change to build a personalized interaction with my child?	To make the statement more specific and reduce the subjectivity, what about 'made efforts to foster personalized interactions with my child'?	why not just fostered pers. Interactions …	CHANGES OK
37	…helped my child access appropriate facilities or services *for my child's* ~~*stim*~~*ulation play* and engagement.	What are these 'facilities for play'? Can provide examples or clarify? Then thought about EIPIC program.	SP did tell us about day respite, someone can watch him for a few hours…	No issues w indicator	No issues w indicator	Instead of 'play', what about 'stimulation' as it will be more widely applicable		
38	… assessed my child's developmental milestones and progress.	No issues w indicator	No issues w indicator. No one assessed him.	They don't because he cannot be assessed. But I go by perception because we have a r/s, not fair just if a line item is NA	No issues w indicator			
39	… informed us of all available *medical* ~~*management*~~ options for my child's condition(s).	All' is really loaded- I don't know that they did. Also, make response options Yes and No.	Can you specify what management? Medical? Financial?	No issues w indicator	No issues w indicator			
40	… clearly explained the advantages and disadvantages of all options for my child so that we could make informed decisions.	I would say 'usually' because sometimes they volunteer the information, but sometimes they need prompting before they give me this information.	No issues w indicator	No issues w indicator	No issues w indicator			

41	… discussed with us how ~~the scope of~~ care could be tailored to *provide* ~~improve~~ *my child's* comfort *for my* ~~child.~~	Add Not applicable option- she doesn't need palliative care. Maybe to make it more broadly applicable, specify provide comfort for my child who has XXX condition.	No issues w indicator	More about giving the parent-caregivers options for home care to make him more comfortable and more efficient, not really care delivered by HCWs.	Stumbled. Not clear. Thinks it's like his chemo can tailor dosage or feeding? Is it more about intensity or dosage of care? Scope… I think everyone wants the widest scope possible but wider doesn't mean more intense but all gentle and mild.			
42	~~… listened to our inputs regarding my child's care.~~ *COMBINE WITH 28*	No issues w indicator	No issues w indicator	Not relevant to me because I'd like to think we make the most rational decision. So it rarely differs from the HCWs' advice. They can't and don't override our decision. They can't stop us. Usually HCWs will listen to parents when things are gray.	No issues w indicator	Suggest to combine with 28	ok	
43	… ~~offered the opportunity for us to be~~ involved *us as much as we wanted to* in decision-making about my child’s care.	Opportunity to be involved' I think is not appropriate, we're the parents… I think it's more about the extent of decision-making.	I think remove the 'offered the opportunity for us to be involved'. We should always be making the decisions, it's ALWAYS on the parents. Re-phrase.	Not relevant to ask like this; it is a conversation. Before then, no one has an answer, weigh the benefits, pros and cons, and usually very useful.	No issues w indicator	To address concerns, removing 'offered the opportunity' and replacing that with 'involved as much as wanted to' may be more accurate.	yes. Offered opportunity is odd	
44	… considered our preferences for treatments when providing care to my child.	No issues w indicator	No issues w indicator	No issues w indicator	No issues w indicator			

45	...were respectful of our spiritual or religious beliefs and practices.	I feel Not Applicable because we haven't had ay requests or interactions about it- no issues, rather. So maybe it's Always? When should Not Applicable be used?	No issues w indicator	No issues w indicator	No issues w indicator			
46	...offered us opportunities to contribute to the community of seriously ill children if we wanted to e.g., letting me support other families, sharing my experience with other parents, or participating in research.	Because our interactions are always all about [her], so this has only come up a few times.	This is the first time. No one offered before.	It's a painful experience to share your exp with other families; they are just trying to help newcomers like us. Maybe rephrase qn more like support u get from other families. It's more a responsible rather than 'high quality' care thing.	No issues w indicator			
47	...offered information on specialized transport for my child.	No issues w indicator	No issues w indicator	No issues w indicator	No issues w indicator			
48	...*addressed* our family's emotional needs related to my child's condition.	Can you add some examples? What does this mean, to 'address'? Does it mean they ask us? Or acknowledge? I feel like this is secondary to [my child].	What does it mean to 'address'? Does texting count? Hospital never talks about our emotional needs. SP texts us and that is actually beneficial but does that count as 'addressed'?	No issues w indicator	No issues w indicator because it's clearly written emotional needs.	Any suggestions from committee on how to clarify what 'addressed' means?		CHANGES OK

49	…referred us to professional psychosocial support when we needed it.	NA- I either didn't need it, or I didn't know that I needed. I also think this should be a Yes/No response, what does it mean to 'always' refer?	The referral to psychosocial support was very unhelpful when actually received. Mother felt the psychiatrist put her at greater risk of suicide. Rephrase to something of 'benefit'.	No issues w indicator	No issues w indicator			
50	…helped us find someone to take care of my child so that we could take a break.	No issues w indicator	No issues w indicator	They offer help. Someone stayed w my kid when wife was delivering.	No issues w indicator			
51	…provided my child with emotional support.	Not Applicable- she is too young.	[He] is non-communicative. So NA.	No issues w indicator	No issues w indicator			
52	… facilitated my child's access to special needs school/day-care.	No issues w indicator	They made that first link for us but then nothing after, does they count as 'facilitated'?	No issues w indicator	No issues w indicator. NA as child is in mainstream school.			
53	… engaged my child's school/day-care *workers?staff? about* ~~on~~ my child's medical needs in school/day-care.	No issues w indicator. They just connected us and then left us to be. They didn't get in touch with the school at all.	No issues w indicator	Don't understand the English. He's receiving some schooling at home so we engage with the school directly. So NA.	Is it engage my child's school/day care providers? To focus on person	about'?	I don't think on is the right preposition	
54	…ensured a smooth transition of care for my child across different care settings *e.g., hospital to home, NICU to CICU*.	No issues w indicator	Need to add examples of where are these care settings.	Thought NA at first. Need to clarify that is ICU to home etc.	No issues w indicator			

55	… attended to my child without undue delay at the Emergency Department.	Again, 'undue' is tricky- I defer to trust rather than my objective knowledge and experience. Context is also important, there was Covid.	Add when self-admitted because if child goes to ED via ambulance it is immediate care. Otherwise, when we self-admit, we wait a long time.	No issues w indicator	No issues w indicator			
55 Did your child spend at least 1 night **in a hospital** in the last 6 months? **YES / NO**								
**IF NO, PLEASE SKIP 56-61 AND GO TO 62.**								
Thinking about all the times when my child stayed **in a hospital in the last 6 months**…								
56	…the diet provided suited my child's *medical* needs.	Noooo. I think should be a Yes/No response	No issues w indicator	No issues w indicator	Again, is it medical or preferential? Medical, yes but preferential, no.			
57	…we were able to stay close to my child.	We didn't get a choice- But I think it was due to Covid.	No issues w indicator	Not appropriate because of COVID. I guess this is a Yes because my wife could access ICU.	No issues w indicator			
58	…Healthcare workers took appropriate action to minimize my child's exposure to infectious diseases.	No issues w indicator	No issues w indicator; they put all the respi kids together to they always catch bugs there.	No issues w indicator	No issues w indicator			

59	…Healthcare workers gave us opportunities to bond with my child e.g., through his/her daily care, activities together.	No issues w indicator	We are always there whether they like it or not. Parents will impose themselves.	No issues w indicator	Bond' is it build r/s with my kid or involve in daily care? I think daily care is just routine but not quality time with my kid, unless for much younger kids that we're not able to interact much with. For older kids, playing, conversations, activities. Add ACTIVITIES into example.			
60	~~…Healthcare workers did their best to create a child-friendly atmosphere.~~	What is a 'child-friendly atmosphere'? What do you mean by this? I mean, they have stickers and stuff?	What does it mean to 'create a child friendly atmosphere'? What is this child friendly atmosphere? I think about nurses going the extra mile, their professionalism, etc.	I don't think it's the HCW's responsibility to create a child-friendly atmosphere. My child is always ICU or HD, child-friendly atmosphere not important as they are bed bound.	No issue w indicator	Suggest to remove this problematic indicator.	ok	
61	…we were given the flexibility to decide who could be at ~~his/her~~ *my child's* bedside in the Intensive Care Unit.	No issues w indicator. Again, not at all- but I think this was more out of the context of Covid.	No issues w indicator	No issues w indicator	Clarify that it's next to CHILD's BEDSIDE.			

62 Has your child been **cared for at home,** in the last 6 months? **YES / NO**

IF NO, PLEASE SKIP 53 – 66.

Thinking about all the times when my child was cared for at home, **healthcare workers**…

63	… made home visits *at sufficient intervals* to support the care of my child .	I don't really know how to respond on the frequency because the Home Care people- they plan, so parents have no control. So frequency-response may be inappropriate.	NA- child has been hospitalized for over 6 months	Not possible to offer daily. Need to qualify how often. Once a month if everything is ok, come in if emergency, things like that.	No issues w indicator	at sufficient intervals'?	suitable is vague	
64	… provided remote consultations.	This makes me think about adhoc situations where I needed to ask for advice urgently on Whatsapp, like number 1.		No issues w indicator	No issues w indicator			
65	…gave me sufficient time to train a long-term caregiver to care for my child *before discharge*.	NA because I was the caregiver.		No issues w indicator	Add BEFORE DISCHARGE, gave me sufficient time…			
66	… did their best to help us avoid unnecessary hospitalizations for my child.	No issues w indicator		No issues w indicator	No issues w indicator			
	LENGTH	OK if you prep respondents, don't let them think it's a short survey.	Ok.	Ok. But if 60 qns to fill out before discharge, will get low response.	Ok. But there were repetitive items, especially surrounding 'compassion', 'listening', that can be combined.			
	OVERALL UNDERSTANDING	Ok.	OK, except the ones discussed.	Ok.	80% ok, some ambiguous and I need to think hard.			
	INSTRUCTIONS	Ok. Didn't read.	Ok.	Ok.	Ok. Skipped it at first.	Cannot rely on long instructions as most don't read.		

Batch 2<br>*Italicized* font and strikethrough: Suggested revisions after steering committee input							
	Over the past 6 months, we…	**P1 (80 mins). Category 3 illness. 14.5 year old male.**	**P7 (65 min). Category 3 illness. 14.5 year old male.**	**P12 (80 min). Category 3 illness. 19 year old male.**	**Facilitator comments**	**Steering committee comments**	**Steering committee comments (cont.)**
1	… had access to a range of medical expertise needed to manage my child's condition(s).	No issues w indicator	**Didn't explore anything new in last 6 months.** Answered always, when asked why, changed to usually.	Very prompted by you. You go to them.	Overall, parents from batch 2 have much older children and have been parent-caregivers for about 12 years. This makes their perspective on the timeframe different when they are such 'seasoned' caregivers. The dynamic changes- they are well-informed, empowered, independent, and taking charge.		
2	… received guidance on how to obtain my child's medical equipment(s) and supplies e.g., medications, medical consumables.	No issues w indicator	No issues w indicator	No issues w indicator			
3	… had access to sufficient financial support for my child's medical expenses *so costs did not stop him/ her from receiving recommended medical care.*	As citizens we always have a kind of citizen subsidy. But other than that, we don't really have. **So I'm not sure what kind of lens I should put here.** Thinking if it's never or seldom.	This one, mm, tricky… right now we have, I supposed. But… **what does sufficient mean right? I wish someone pays fully.** Right now I still have like family savings that can cover at this point but.. Thinking about potentially more subsidies and all those.. there is quite limited support due to income range... means testing is usually very hard to meet. So 'sometimes'.	No issues w indicator	How about qualifying 'sufficiency' with "had access to sufficient financial support for my child's medical expenses **so costs did not stop him/her from receiving medical care"? That makes it clearer that ability to receive medical care indicates sufficiency.**	the logic becomes quite complex. I would try to keep it simpler, iether jus the access part of the cost did not part	Added 'recommended'

			Because we know those subsidies exist but we're not eligible.				
4	… had a healthcare worker/team that coordinated my child's care across different care services.	No issues w indicator	No issues w indicator	Not quite sure which to choose because it's there but I have to ask for help and be thick skinned to get that service.			
5	… received consistent information from different healthcare workers.	Because he's under many specialists, not patient-centric they don't come tgt, just individually there's a pri doc and the various specialists. Every discussion, the parents are the one with the overall picture. So we always need to cross check, check last consultation from various specialists.	No issues w indicator	No issues w indicator			
6	… had access to sufficient financial support for my child's non-medical expenses e.g., special needs education, speech therapy etc.	I apply same rules as 3, to be consistent. Really non-medical expenses we're still on our own.	No issues w indicator	Always because I look for the appropriate information			
7	… received appropriate allied health support (e.g., physiotherapists, speech therapists) to meet our goals for my child's development.	No issues w indicator	No issues w indicator	No issues w indicator			

8	***In the last 6 months, did your child formally receive any advice or care from a palliative or supportive care team or specialist(s)? YES / NO / I DON'T KNOW*** *Description: *Palliative care for children is the active total care of the child's body, mind and spirit, and also involves giving support to the family.*<br>*… were able to discuss ~~my child's treatment plan with a palliative care team or palliative care specialist(s) if we wan~~ted ~~to.~~*	Yes and no. He was dischared under pall care and deemed stable. Pall care has a diff qualifying criteria to admit patients under their care, And we were discharged from their care in 2019 (for now) but im still in touch on personal basis so if we need any advice i still can. So formally, no, but functionally yes. [Ticked No]	*In the last 6 months,* **did your child FORMALLY receiv~~inge~~ any advice or care from a palliative or supportive care team or specialist(s)? YES / NO**<br>**Palliative care is the active holistic care of individuals with serious health-related suffering due to severe illness, aiming to improve the quality of life of patients, their families, and their caregivers (WHO). The service ~~provides a holistic care approach via the multidisciplinary team that encompasses psychological, emotional and spiritual support. Supportive care and palliative medicine is appropriate at any age and at any stage in a serious illness, and it can be provided along with curative treatment.~~*	No issues w indicator	This construct is very tricky.<br><br>We similarly need to decide if **formal/specialist** palliative care is the focus, or if **generalist and/or informal palliative care** support should be included. I think we should ask about FORMAL provision of palliative care services. ICPCN's definition: ICPCN: Palliative care for children is the active total care of the child's body, mind and spirit, and also involves giving support to the family.	be a little more humble with the definitiom. What if they got soething that was a simple version than what you define	
9	We were introduced to a palliative or supportive care team *or* specialist(s) at an appropriate time.		Does it mean an actual, real dedicated pall care or if I feel I get sufficient support from hospital/school/social worker? So formal or informal service or just whether I feel supported? **If not formal, then this questions is already similar to 8 and 5, like a team.**	No issues w indicator			ADDED 'OR'
**Over the past 6 months, my child's healthcare workers…**							
	It's been so many years and he knows we are capable. Our relationship has progressed and is now very stable. So 6						

	months is too short since we barely see anyone now.						
10	… ~~had a healthcare worker/team that supervised the management of my child's medical needs~~ *…worked together to ensure my child's medical condition(s) are well managed.*	No issues w indicator	Not sure. If he has a few doctors… from what I understand [the hospital's] doctors work as a team. They do kind of communicate. So heart doctor has his genetic notes etc. It's more about whether his medical needs are well-managed by the various disciplines without miscommunication. Whether or not they are all on the same page, whether it's one person or one team, it's no different from parent's perspective. So it's more important that they're all well informed and no miscommunication happens.	No issues w indicator	As pointed out by P7: having someone supervising the management of child's medical needs is less important than the medical needs being actually well-supervised/managed. That is the ultimate goal. Hence, What about changing to **"worked together to make sure his/her medical condition(s) are well managed.."?**	communicated well to make sure .. Is also a complicated logic	Communicated well with who? Each other? I don't think they would know this
11	…were approachable when we needed advice about my child's care .	No issues w indicator	No issues w indicator	No issues w indicator			
12	…worked *together* ~~as~~ *a* ~~team~~ towards common goals for my child's care.	No issues w indicator	No issues w indicator	No issues w indicator	If indicator 10 is rewritten, this becomes slightly repetitive although not entirely the same. 10 focuses on management while 12 focuses on common goals.		
13	…organized my child's appointments to reduce our hospital visits.	No issues w indicator	No issues w indicator	No issues w indicator			

14	…put in effort to build a trusting relationship with us.	No issues w indicator	No issues w indicator	No issues w indicator			
15	…kept us well informed about my child’s condition.	No issues w indicator	Hesitated- then agreed, but that it's parents who usually inform them about child's condition than other way around.	Usually we are the one who keeps them informed.			
16	…communicated with us in a way that was sensitive to our needs.	No issues w indicator	No issues w indicator	No issues w indicator			
17	…gave us enough time to think about decisions for my child's care.	No issues w indicator	No issues w indicator	No issues w indicator			
18	*…were responsive in ~~did all they could to identifying and~~ managing my child's medical issues.*	No issues w indicator	I feel that this is unfair because it's very perception-based, not necessarily fair. It's purely how I feel. It may not reflect the truth. I think it should not be asked because I'm not involved, I can only see when I visit, but behind the scenes? Do they discuss or not at all? Not something I can judge.	Discussion basis. Open consultation. Other parents come to me because I answer faster! And they look to me as authority. But some parents feel more comfortable and confident talking to me. Because if I call [the hospital], I can't reach ANYONE. They tell you they revert in 3 days etc. Hospitals are very 'administrative' and they feel scared to ask doctors and they don't dare to ask on weekends e.g. my son threw up his medicine. Can you separate this question into those on a maintenance basis- open date, versus urgency basis with regular appointments?	In response to P7 and p12, what about rewriting indicator to focus on responding to parental priority for responsiveness? **"…were responsive in identifying and managing my child's medical issues"?**	identifying AND managing. Double barrelled?	
	~~…treated my child in a timely manner.~~	No issues w indicator	No issues w indicator	No issues w indicator	**If we modify indicator 18, this indicator becomes repetitive.**		

19	…avoided treatments and investigations that were not aligned with our goals for my child’s care.	No issues w indicator	No issues w indicator	No issues w indicator			
20	…managed my child's physical symptoms to make sure he/she was comfortable.	No issues w indicator	No issues w indicator	Sometimes I had to adjust my expectations because they can't resolve his symptoms.			
21	…took responsibility for my child's wellbeing when he/she was under their care.	No issues w indicator	No issues w indicator	No issues w indicator			
22	…~~told us~~ *kept us up-to-date about* what to look out for so that we knew when my child was unwell.	I have a problem with 'over the past 6 months'; because he has been quite stable. Episodes of dystonia etc happened much earlier. So if limited to 6 months not accurate. Now more stable so the time limit is tricky. So tweak to past 6 months OR significant milestones during treatment? Cos milestones I can easily remember the high need period, for abt 2 years in a row where his condition was unstable. And they told us how to recognize his symptoms.	No issues w indicator	Not relevant; we haven't seen the doctor for more than 6 months	To encompass parents whose child is unstable & also parents whose child's condition is more stable, would **"kept us up-to-date about what to look out for so that we knew when my child was unwell"**?	we .. We .. My.. Inconsistent	
23	…equipped us with skills so that we could confidently care for my child.	No issues w indicator	No issues w indicator	No issues w indicator			
24	…acknowledged and affirmed our efforts in caring for my child.	No issues w indicator	No issues w indicator	No issues w indicator			
25	…listened to us when we advocated or spoke up for my child.	No issues w indicator	No issues w indicator	No issues w indicator			

26	…showed us care and concern.	No issues w indicator	No issues w indicator	No issues w indicator			
27	…supported our hopes for my child.	No issues w indicator	What does that mean? I didn't share with them my hopes… doctors will never say all will be fine, they always give the worst case scenario so I think it conflicts with what they are supposed to do. I don't expect the HCWs to support my hopes for my child. So I would say seldom but I don't expect that of them, not a fair expectation of the HCWs. Unless it's for social workers only, maybe it's more about your mental health, being encouraging and supportive.	No issues w indicator	P7- what if parents don't even share their hopes? She rightly pointed out whether or now HCWs know their hopes, it's about being supportive 7 encouraging.		
28	…prepared us for what may lie ahead.	No issues w indicator	No issues w indicator	Not relevant to me; because [my child] is already 19 but very very delayed. And from a developmental angle, we don't know what their timelines are. Versus when he was younger and they could try to manage our epectations. But we don't ask these qns anymore, we just focus on trying to catch up. I think you should ask 2 separate questions, and Specify medical and developmentally, what lies ahead?	Do not think there is a need to separate medical from developmental as they are heavily inter-dependent.		
29	…provided us with a compassionate listening ear.	No issues w indicator	No issues w indicator	No issues w indicator			

30	…guided us on how to reduce my child's medical costs.	No issues w indicator	No issues w indicator	No issues w indicator			
31	… fostered personalized interactions with my child.	No issues w indicator	No issues w indicator	No issues w indicator			
32	…helped ~~my child~~ *us* access appropriate facilities or services for my child's stimulation and engagement *e.g., disability-friendly facilities, early intervention centres*.	No issues w indicator	Add examples because it's a bit awkward to read.	This one… after graduating from school, services are TERRIBLE. Really, really terrible. Parents of young adults are struggling. They turn away most of us due to vacancies, where there are programs, staff are not well trained to care for special needs, so they turn us away because of lack of training. It's REALLY terrible. A lot of focus is on pediatrics. You guys shd look at for 18 and above or below. It's complete exclusion. I'm pissed off with the word inclusivity, because its very specific only for certain disabilities.	P12 points out key structural differences for pre- and post-18 years. Suggest we keep to 18 years and below to focus on children still within the pediatric health+social care system.	drop 'my child's"	CHANGED 'MY CHILD' TO 'US'
33	… assessed my child's *physical, cognitive and emotional development* ~~*developmental milestones*~~ and progress.	No issues w indicator	Add an NA option because he is deteriorating. He has no usual developmental milestones, so it's not so relevant besides newborn development.	NEVER. I had to do it all myself. And there is a huge gap: my child aged above 16, but has the brain and body of a child. Because the adult diseases set in, but the body is developmentally challenged from the angle of a pediatric person. Adult doctors and therapists cannot address our kids' needs.	Although inaccurate, most think developmental milestones=newborn milestones. **Suggest a broad yet accurate description of a child's holistic development: 'physical, cognitive and emotional development',** which essentially defines developmental milestones and yet is accurate + relevant to parents whose children have diff developmental pathways.		

34	… informed us of ~~all~~ *the range of* available medical options ~~for~~ *to manage* my child's condition(s).	Because he has rare condition, we don't have a specialized doc. So if I put in terms of the symptom control, his daily condition, then yes, I would put usualy. But if im looking for more advancement in options or technologies around the world versus what i'm offered, doctors don't offer us. The parents are the one who keep tabs and bring it up to the doctor.	We don't know other options, sometimes we search and we ask the doctors but otherwise we don't know.	No issues w indicator	All' is too big a word, but but alludes to the importance of giving choices. What about "informed us of the range of medical options 'to manage' my child's condition(s)?"	all is a big word	
35	… clearly explained the advantages and disadvantages of all options for my child so that we could make informed decisions.	No issues w indicator	No issues w indicator	No issues w indicator			
36	… discussed with us how care could be tailored to improve my child's comfort.	No issues w indicator	No issues w indicator	No issues w indicator			
37	… involved us as much as we wanted to in decision-making about my child's care.	No issues w indicator	No issues w indicator	No issues w indicator			
38	… considered our preferences for treatments when providing care to my child.	No issues w indicator	No issues w indicator	No issues w indicator			

39	…~~*offered us opportunities*~~ *asked us if we wanted* to contribute to the community of seriously ill children ~~*if we wanted to*~~ e.g., letting ~~*me*~~ *us* support other families, sharing ~~*my*~~ *our* experience with other parents, or participating in research.	No issues w indicator	No issues w indicator	This was Never discussed- so it is NA or Never? A lot of families are just worried about themselves. It has to come from the heart. It's not about offering us, but it's about US doing it. Families who are willing to help will offer the help. Perhaps rephrase it: Have you been asked? You should ask parents regularly if they are willing to help other families. We have plenty of capable parents who are willing to help but never asked.	Taking P12's suggestion and feedback: What about "**… asked us if we wanted to contribute to the community of seriously ill children e.g. letting me support other families…**"		SHOULD IT BE 'MY' OR 'OUR'?
40	…communicated with my child in a way that was sensitive to his/her needs. *ADDED NA OPTION*	My child cannot comprehend and cannot communicate so you should add an NA option.	Add an NA option because my child is non-communicative.	I don't know how to answer. My child is non-verbal but he has thoughts and feelings. They don't have the time to understand him. And parents don't want to pay for the extra time.			
41	…were respectful of our spiritual or religious beliefs and practices.	No issues w indicator	No issues w indicator	No issues w indicator			

42	…~~facilitated our~~ *helped us to* access ~~to available~~ parent support *groups* ~~networks.~~	Usually or sometimes- not so much because they're not willing but because the parent support doesn't exist… I myself had to lead a support group. So it depends a lot on HCW awareness because a lot of us are informal. If the doctors didn't journey with us, they wouldn't be aware of the informal support groups like us. Can you add a statement to see how aware the HCWs are about these networks? Separate from whether they facilitate access?	No issues w indicator	No issues w indicator	Not direct feedback but wondering if **'helped us to access parent support groups'** may be simpler, more colloquial? Will add an NA option.	na if such groups don't exist	
43	…offered information on specialized transport for my child.	No issues w indicator	Can't remember… I should put NA because I didn't ask for it. So I don't have the experience	No issues w indicator			
44	…addressed our family's emotional needs related to my child's condition.	No issues w indicator	No issues w indicator	No issues w indicator			
45	…referred us to professional *emotional* ~~psychosocial~~ support when we needed it.	No issues w indicator	No issues w indicator	No issues w indicator	Since we use 'emotional support' in indicator 47, suggest we change this to the same for consistency.		
46	…~~helped us find~~ *found* someone *else* to take care of my child *when we needed help* ~~so that we could take a break~~.	We know they exist but we don't know if they will meet his needs and also very costly. Checking into hospice care is too costly. I put seldom because we didn't ask but they didn't offer. *DIDN'T PUT NEVER BECAUSE THE SERVICES EXIST*	Not sure whether to use NA or never. Never because I never asked or because they never offer? I would have thought of NA because I didn't ask. I don't think it's an expectation of HCWs… maybe friends, relative or other parent support groups.	No issues w indicator	Parents struggled to answer this item because they did not ask for it. I think we need to clarify "**when we needed help**"	my … we..	

47	…provided my child with emotional support.	No issues w indicator	No issues w indicator. NA but stumbled because child is quite nonresponsive.	No issues w indicator			
48	… ~~facilitated~~ *helped* my child~~'s~~ access ~~to~~ special needs school/day-care.	Stumbled- to what expectation is 'facilitation'? To gain entry, to write a referral letter is no problem. But for rare disorder, it's a problem. If he is not ASD or down syndrome etc, there are few schools. Always long queue and we are always behind. So the qualification criteria... there's nothing within the system that allows us to put in our child.	No issues w indicator. Write referral.	Again, I think this is only relevant up till 18 years old only then we are kicked from the system.	Need to clarify what counts as 'facilitated' if we want to use that word as it's tricky. Why not same as indicator 34, "helped my child access", which is less demanding?		
49	… engaged my child's school/day-care staff about my child's medical needs in school/day-care.	No issues w indicator	No issues w indicator. They write memos on his conditions, and referrals, etc but maybe not so much engage. They don't directly engage, they engage through me- so Engage is too much, communication is too much (unless you clarify it).	No issues w indicator			
50	…ensured a smooth transition of care for my child across different care settings e.g., hospital to home, NICU to CICU.	No issues w indicator	No issues w indicator	No issues w indicator			
51	… attended to my child without undue delay at the Emergency Department.	No issues w indicator	No issues w indicator	No issues w indicator			
Did your child spend at least 1 night **in a hospital** in the last 6 months? **YES / NO**							
**IF NO, PLEASE SKIP 56-61 AND GO TO 62.**							

Thinking about all the times when my child stayed **in a hospital in the last 6 months**…					
52	…the diet provided suited my child's medical needs.	NA. Because it's only asking about the last 6 months- we do yearly sleep study for him. He's on a special diet, they don't have the facilities to prepare his diet. But it's still important to ask. Because a lot of families on special milk. And we need to give lead time to bring in the right milk for his stays.	NA; not hospitalized	NA; not hospitalized	
53	…we were able to stay close to my child.	No issues w indicator			
54	…Healthcare workers took appropriate action to minimize my child's exposure to infectious diseases.	No issues w indicator			
55	…Healthcare workers gave us opportunities to bond with my child e.g., through his/her daily care, activities together.	No issues w indicator			
56	…we were given the flexibility to decide who could be at my child's bedside in the Intensive Care Unit.	No issues w indicator			
Has your child ***lived at home*** ~~***cared for at home,***~~ in the last 6 months? **YES / NO**		This question is not clear- meaning bed bound? Cannot go to school? Very confusing. Clarify from a medical or social perspective? Too wide. Now we're all alone- we have no one who is regularly connected to us.	Maybe make it simpler: "Has your child been living at home, in the last 6 months?"		

**IF NO, PLEASE SKIP 53 – 66.**							
Thinking about all the times when my child lived at home ***in the last 6 months*~~, health~~care workers**…							
57	…~~made home visits~~ *provided sufficient consultations (physical and/or*~~—~~ ~~*remote)*~~ ~~*at sufficient*~~ *intervals* to support the care of my child .		No issues w indicator. Initially answered always but actually was not referring physical consultations- only realised difference after reading 58. Please Clarify.	My child is out of the pediatric system.	Both P1 and P7 share key info; not all parents will feel the want or need for physical consult. More about meeting parent's needs while caring for child at home by being available to be consulted. Combine 57 and 68?		
	~~… provided remote consultations.~~	Thinks about emails. Can get in touch that way.	Can combine this with 57? Because we choose teleconsult, it's cheaper and also physical home visit was stopped during Covid. They only opened up recently but they give us option. And we still chose teleconsult. More about meeting our needs, his stability.	My child is out of the pediatric system.			
58	…gave me sufficient time to train a long-term caregiver to care for my child before discharge. *ADD NA OPTION*	No issues w indicator	No issues w indicator	NA because I'm the long term caregiver	Add an NA option		
59	… did their best to help us avoid unnecessary hospitalizations for my child.	No issues w indicator	No issues w indicator	Stumbled. Ihave to always ask for help. Not them asking me whether I need help. So this is not realistic. They don't check on him.			
	LENGTH		Ok.	Ok. Experience is fine.			

	OVERALL UNDERSTANDING		Ok. A lot of them I think about whether or not I expected the HCWs to do this and that. Others should not be ambiguous. Shift definition of HCWs into the question page, repeat at the top.	*Narrow the age to below 18. Then, child is usualy transitioned.<br>*Trying to cover medical + social services is really hard. Now, our minds are so separated: School vs medical. So it's hard to capture information about both. Have to capture each part separately.<br>*Definition of HCWs can be clearer: doctors, nurses? MSW? Because not representative of the support we get as parents. Schools are important.<br>*REPEAT HCW definition on each page.<br>*AND PRIVATE VS PUSLIC SECTOR. Private is very money-driven. Therapists from hospital may give free consultations etc. I think mindset is always hospital-based because my interpretation is limited to hospital so my exp is biased to the hospital. *When using the work 'healthcare' only because we then exclude our important players. So when we read the qn, if i read 'healthcare', I only think of hospital. And I struggle.<br>*Can you add a question: what do you hope to see moving forward?	SUGGESTIONS FOR THE NEXT ITERATION TO BE TESTED:<br><br>1. Remind who HCWs cover every other page<br>2. Reduce age coverage to 18 years old and below.<br>3. Remind that 'healthcare' covers both health (e.g. hospitals) and social care (e.g. community services) settings<br>4. Replace use of 'healthcare' with 'care' i.e. 'care workers' and 'care settings', to reduce the hospital-bias		
	INSTRUCTIONS	Ok.	Skipped the instructions altogether. Too long	6 months is too short- covid in the way, lots of limitations,			

				and he is quite stable. Maybe without Covid it would have been different?<br>For kids after 18 like me, we are really on our own. The healthcare workers just refer us out to [a government agency for adults with disabilities]. So maybe reduce the scope to kids who have not graduated from schooling? Like before 18? Because after that EVERYTHING changes- medical, healthcare, social workers, accessibility etc. Totally different ballgame. So think about it- because [the country's] system stops at 18, because anything pediatric stops at 18.			

Batch 3
Italicized font and strikethrough: Suggested revisions after steering committee input

	**Over the past 6 months, we…**	**P10 (80 mins) Category 2 illness. 13.5 year old boy.**	**P4 (55 mins). Category 3 illness. 5.5 year old boy.**	**P11 (50 mins). Category 4 illness. 5.5 year old boy.**	**Facilitator comments**	**Steering committee comments**	**Steering committee comments (cont.)**
1	… had access to a range of medical expertise needed to manage my child's condition(s).	No issue with indicator	No issues with indicator	No issues with indicator			
2	… received guidance on how to obtain my child's medical equipment(s) and supplies e.g., medications, medical consumables.	No issue with indicator	No issues with indicator	No issues with indicator			

3	… had access to sufficient financial support for my child's medical expenses so costs did not stop him/her from receiving recommended medical care.	Parents will just laugh because for middle class parents we all wish we had more financial support. Means testing.. Yeah. But it's subjective in that way, we can't ask for it if we're not able to access because of the means testing.	No issues with indicator	No issues with indicator	AS P10 says, it might be subjective, but the intent of this indicator is to elicit self-perceived financial sufficiency. With the added "so that costs did not stop child from receiving medical care' I think it is less problematic.	I don't think 'so that costs…etc" makes it clearer - may be rather less clear what exactly you mean	looks good to me.
4	… had a ~~*healthcare*~~ worker/team that coordinated my child's care across different care services.	No issue with indicator	No issues with indicator	No issues with indicator			
5	… received consistent information from different healthcare workers.	No issue with indicator	Initially struggled to understand what is 'consistent' then said Ah ok 'aligned information'.	No issues with indicator			
6	… had access to sufficient financial support for my child's non-medical expenses e.g., special needs education, speech therapy etc *so that costs did not stop him/her from receiving recommended non-medical care.*	Same as 3… I'm with [a charity] so we have access to some support. But never sufficient, it's just SOMETHING. 'Sufficient' is very subjective but another word to capture that 'sufficiency', or broken down more into diff questions: 1. evidence of means-testing?	I think parents want to say something more, add a free-text box as it depends on how much help they get; some really might not be aware. And big differences for children with insurance vs no insurance. They might really want to voice up their struggles for financial indicators.	No issues with indicator	Am ok to add a free-text box if the committee is in agreement. Also suggest to qualify like indicator 3 with "so that costs did not stop him/her from receiving recommended non-medical care"	same concern with 'so that costs etc" - does it really help clarify? Also, a free text box makes the scale more complex to score. How would you use it? Is it just an opportunity for them to share their feelings ? Cannot see how you easily can embed it in an overall score	I like the red. I don't think free test is very useful.

7	… received appropriate allied health support (e.g., physiotherapists, speech therapists *and care from other non-doctors or nurses)* to meet our goals for my child's development.	No issue with indicator	I don't understand the term 'allied health support'. To me, as I'm not in the medical field, I don't know the term and the allied health term distracted me.	I want to elaborate more; our govt. therapy support is insufficient. From a hospital, early intervention perspective, not enough. Their skills, their frequency, none of it is enough. We can't quit our jobs to attend with them. So I seek out private therapy, which is costly, but we have to balance cost with effectiveness for [him]... it's all connected, they have to be paid better, which will drive up cost too, but that's fine. Hospital needs more and more experienced therapists.	Am ok to add a free-text box if the committee is in agreement. Unfortunately I don't think there is a more layman term for allied health.	same concern with free text box. What would it be for? Adding text boxes changes the nature of the overall scale. I think allied health is ok since you do try to explain in parentheses. But can you give better definition, 'e.g' = for example - is not so clarifying.	I would avoid free text. See if my change is ok.
8	In the last 6 months, did your child formally receive any advice or care from a palliative or supportive care team or specialist(s)? YES / NO / I DON'T KNOW *Description: *Palliative care for children is the active total care of the child's body, mind and spirit, and also involves giving support to the family.* *Pediatric Palliative care is specialized medical care for people living with a serious illness. Palliative care focuses on providing relief from the symptoms and stress of the illness. The goal is to improve*	No issue with indicator	No issues with indicator	Don't know what is palliative care and the definition is not clear. And no one focuses on his mind and spirit sounds very 'cheem' [slang for complicated], and not used in our Asian context. The definition is not helpful.	The definition tested is used by both WHO and ICPCN. But to parents this is very vague. What about: Pediatric Palliative care is specialized medical care for people living with a serious illness. Palliative care focuses on providing relief from the symptoms and stress of the illness. The goal is to improve quality of life for both the child and the family.	current definition sounds ok	In the last 6 months, did your child formally receive any advice or care from a palliative or supportive care team or specialist(s)? YES / NO / I DON'T KNOW *Description: * Pediatric Palliative care is specialized medical care for people living with a serious illness.The goal is to improve quality of life for*

	quality of life for both the child and the family.						*both the child and the family.*
9	We were introduced to a palliative or supportive care team or specialist(s) at an appropriate time.	No issue with indicator	No issues with indicator				
**Over the past 6 months, my child's healthcare workers…**							
10	…worked together to ensure my child's medical condition(s) are well managed.	No issue with indicator	No issues with indicator	No issues with indicator			
11	…were approachable when we needed advice about my child's care .	No issue with indicator	No issues with indicator	No issues with indicator			
12	…worked together towards common goals for my child's care.	No issue with indicator	No issues with indicator	No issues with indicator			
13	…organized my child's appointments to reduce our hospital visits.	No issue with indicator	No issues with indicator, but no need to repeat the 'Always to Never' headers again	No issues with indicator			
14	…put in effort to build a trusting relationship with us.	No issue with indicator	No issues with indicator	No issues with indicator			

15	…kept us well informed about my child's condition.	No issue with indicator	No issues with indicator	No issues with indicator			
16	…communicated with us in a way that was sensitive to our needs.	No issue with indicator	No issues with indicator	No issues with indicator			
17	…gave us enough time to think about decisions for my child's care.	No issue with indicator	No issues with indicator	No issues with indicator			
18	…were responsive in managing my child's medical issues.	No issue with indicator	No issues with indicator	No issues with indicator			
19	…avoided treatments and investigations that were not aligned with our goals for my child's care.	No issue with indicator	No issues with indicator	No issues with indicator			
20	…managed my child's physical symptoms to make sure he/she was comfortable.	No issue with indicator	No issues with indicator	No issues with indicator			
21	…~~*took responsibility for*~~ *ensured* my child's wellbeing when he/she was under their care.	No issue with indicator	"to ensure my child's well-being"? I prefer that as then I feel the assurance from HCWs that they are caring for my child	No issues with indicator			ok
22	…kept us ~~*up-to-date*~~ *updated* about *symptoms* ~~*what*~~ to look out for so that we knew when my child was unwell.	No issue with indicator	Stumbled on 'kept us up-do-date'- meaning there's changes along the way… means catching symptoms… the sentence, I have to pause because I'm distracted by 'up-do-date'. "Kept us updated of the symptoms that we should know when my child was unwell"	No issues with indicator	Replace 'up-to-date' with 'updated' and 'what' to look out for with 'symptoms' to look out for.		ok

23	…equipped us with *knowledge and* skills so that we could confidently care for my child.	No issue with indicator	‘Skills' doesn't seem like the right term. Caregiver knowledge is more suitable.	No issues with indicator	Add in 'knowledge'?	ok. But knowledge and skill are not the same, do you now make it into a double barreled question	ok
24	…acknowledged and affirmed our efforts in caring for my child.	No issue with indicator	No issues with indicator	No issues with indicator			
25	…listened to us when we ~~*advocated or*~~ spoke up for my child.	No issue with indicator	Just use advocate OR spoke up, keep things simple.	No issues with indicator			
26	…showed us care and concern.	No issue with indicator	No issues with indicator	No issues with indicator			
27	…supported our hopes for my child.	No issue with indicator	Stumbled. Repeated statement a few times. Is it goals? But hope is a better work because we hope rather than set goals. But I think the verb 'supported' doesn't align with 'hopes'. I think 'supported our goals for my child' is more accurate for the word support.	We never really verbalized what's our hopes for my child. This is more abstract. Not so relevant because our hope is… [sighs] what do we hope for? Doctors always give 'safe' answers. They don't really 'support' anything, they will just give generic answers or tell us the hard truth and it's a hit on the head.’	Goals' are not the same as 'hope'; hope is abstract and transcends reality. The key is that HCWs do not 'crush' parental hopes, so agree that 'supporting' hopes is not appropriate- but I struggle to improve this.	you can squash, crush or kill hope. But support hope sounds odd. Help maintain hope?	I think hopes is ok. I don't like 'our hopes' and 'my child' but leave that to you.
28	…prepared us for what may lie ahead.	No issue with indicator	Stumbled at 'lie ahead'. What about 'prepared us for the possible journey ahead'.	No issues with indicator	Suggest no change		
29	…provided us with a compassionate listening ear.	No issue with indicator	No issues with indicator	No issues with indicator			

30	…guided us on how to reduce my child's medical *expenses ~~costs~~ e.g. access to subsidies, financing schemes*.	Hesitated. Not sure what it actually is asking about: HCWs? I always stumble on the cost-related items… I don't think they ever talked about reducing medical costs… Can you add examples? E.g. Access to funding/subsidies, schemes.. Otherwise it doesn't jump out, since hospitals always take money from us. Most medical social workers always talk about selling our car, cutting your insurance stuff like that, so if you clarify it's about schemes etc, it'll be clearer.	Use 'expenses' rather than costs.	No issues with indicator	Suggest to replace costs with 'expenses' and add in examples.	strictly speaking the child does not have any expenses, it is the family or parents who do. But may be ok.	
31	… fostered personalized interactions with my child.	No issue with indicator	No issue with indicator	No issues with indicator			
32	~~*…helped us access appropriate facilities or services for my child's stimulation and engagement e.g., disability-friendly facilities, early intervention centres, special education*~~ *schools*.	Appropriate services; WHAT are these, and WHO for? Specify. For Below 7, schools for 7 onwards (both special education (SPED) and mainstream schools). For non-SPED schools, what are the facilities? Because access Disability-friendly facilities, no. Early intervention centres, only for younger parents. For us on the older side, for him what we're looking at, usually it's just school itself- school-wise, the teachers do tell us about this. Can you break down care into schools also (and specify that SPED teachers are also social care workers). What are 'disability-friendly facilities'?	A mouthful, but can understand.	No issues with indicator	Suggest to remove this indicator- with all the changes suggested it would already be covered by 'allied health support' and 48 on access to special needs schools/daycare	ok	ok

		e.g. playgrounds, not many know. Government early intervention programs, everybody knows. For older kids, it will be SPED schools. 18 and above, what then? That their care has been taken over well by DAC, daycare centres? Or some facility also? If we consider those, healthcare workers may not discuss any of these. Once kids are older, it's SPED teachers that inform parents of everything so best to specify SPED schools inside. Also, 'disability-friendly facilities' is very broad; maybe take that out and focus on the services.					
33	… assessed my child's physical, cognitive and emotional ~~development and~~ progress.	No issue with indicator	No issues with indicator	I don't know if there's proper 'assessment' of his development and progress, it's more like when we see the doctor she will just observe him but I don't know if it's meant to be more medically tested or not. Can you clarify if it's some formal medical assessment or general doctor observing him?	I think likely no need to change- it can be either 'formal' or 'informal'		changes ok
34	… informed us of the range of available medical options to manage my child's condition(s).	No issue with indicator	No issues with indicator.	No issues with indicator			
35	… clearly explained the advantages and disadvantages of all options for my child so that we could make informed decisions.	No issue with indicator	No issues with indicator	No issues with indicator			

36	… discussed with us how care could be tailored to improve my child's comfort.	No issue with indicator	No issues with indicator	No issues with indicator			
37	… involved us as much as we wanted ~~to~~ in decision-making about my child’s care.	No issue with indicator	Remove the 'to'	No issues with indicator			
38	… considered our preferences for treatments ~~*when providing care*~~ *given* to my child.	No issue with indicator	"considered our preferences for treatments to my child" is sufficient.	No issues with indicator	Replace 'when providing care' with 'given'		ok
39	…asked us if we wanted to contribute to the community of seriously ill children e.g., letting us support other families, sharing our experience with other parents, or participating in research.	No issue with indicator	"this" puts us more cohesively together, "this community of seriously ill children"	No issues with indicator	I suggest not to use 'this' community; it might spark a sense of isolation for parents not actively part of the community.		ok
40	…~~*communicated with*~~ *treats* my child in a *kind and respectful way* ~~*that was sensitive to his/her needs*~~. *AND REMOVE NA OPTION*	This is a tricky question because my child is non-verbal, but non-verbal cues are still relevant. So it's not true that it's always NA just because child is non-verbal. But the use of the term 'sensitive' is tricky; what about was kind and respectful in communicating with my child? Because we cannot except them to be sensitive, they don't know our child. But we as parents still want people to be kind and most importantly respectful to the child. Whether they are verbal, non-verbal, we want our child to be treated with respect. 'Sensitive' is very subjective.	No issues with indicator	No issues with indicator	Agree that we have met a lot of pushback with this item due to many children being non-verbal; parents struggle to define what is 'sensitive' to a non-verbal child. P10's suggestion is effective: treats my child in a kind and respectful way'?	ok	ok

41	…were respectful of our spiritual or religious beliefs and practices.	No issue with indicator	No issues with indicator	NA- in a way, yeah, none of the doctors or nurses say anything with regards to this, which is good lah.			
42	…helped us to access parent support groups.	AH. OK. MM… sometimes because a lot of the time doctors don't, maybe due to PDPA. It's mostly organically, parent to parent and we reach out via internet.	No issues with indicator	No issues with indicator			
43	…offered information on specialized transport for my child.	No issue with indicator	No issues with indicator	No issues with indicator			
44	…addressed our family's emotional needs related to my child's condition.	No issues with indicator	No issues with indicator	No issues with indicator			
45	…~~referred~~ *offered* us ~~to~~ professional emotional support *e.g. counsellors, if* ~~when~~ we needed it.	No issue with indicator but therapists are not cheap… it's a chicken or egg situation? And I had to ask for it.	No issues with indicator but add examples e.g. psychologists, MSWs…	No issues with indicator	CHANGE 'when' to 'IF' and 'referred us' to 'offered us'?	unclear whose support they are offering. Their own or somebody else's?	ok
46	…*helped us to find* ~~found~~ someone ~~else~~ to take care of my child when we needed help *e.g. respite care, hospice care*.	Can you add an example e.g. respite care or hospice care	"helped us to find someone", cannot use 'found' someone… "when we needed this help"	No issues with indicator	Add examples and add 'help' us to find someone (similar to original)		ok
47	…provided *emotional support to* my child *e.g. music therapy, counselling* ~~with emotional support~~.	Add example, what is emotional support? E.g. Art therapy	"provided emotional support to my child"	I think this is not applicable. How do they provide him with emotional support- how? Every time we visit them it's very tasked focused. He's not verbal…. So how to emotionally support him?	Add example and switch sentence structure around.	is music therapy really emotional support? It can be, but it can be other things as well.	ok
48	… helped my child access special needs school/day-care.	No issue with indicator	No issues with indicator	No issues with indicator			

49	… ~~*engaged*~~ *communicated* my child's *medical needs in* school/day-care *to their staff* ~~*about my child's medical needs in*~~ *school/* ~~*day care*~~ ~~*e.g. do*~~*ctor talked to the school about my child's feeding needs.*	Don't understand what is 'engage the school'? What about 'connect with'? And add an example like 'doctor talks to school about child's medical needs'	Stumbled on "engaged". It's more like "informed" my child's school/day care staff about… but informed is too shallow, there's some handover of information too. Need to think about that verb, get a Masters in English (laughs)	No issues with indicator	Communicated may be a better word for engage that is less contentious? And add example: "communicated my child's medical needs in school/day-care to their staff e.g. doctor talked to the school about my child's feeding needs."	complicated sentence?	ok
50	…ensured a smooth transition of care for my child across different care settings e.g., hospital to home, NICU to CICU.	No issue with indicator	No issues with indicator	No issues with indicator			
51	… attended to my child ~~*without*~~ *within a reasonable amount of time* ~~*undue delay*~~ *when we brought him/her to* — *at* the Emergency Department.	Hospital needs to work on this process. I Hesitated in choosing an answer. What is 'undue delay', is it immediately? Because they have so many protocols, what can or cannot be passed? Process-issues. What's the purpose of asking this in the survey? Might need to ask extra questions if you want to focus on this. Because if my child is very sick, then confirm. So need more context e.g. 'if child is very sick', then at moment at triage, then yes. So be more specific.	No issues with indicator	No issues with indicator	We got VERY similar comments from P3 and P6 from Batch 1 of parents. Suggest to revise to focus on self-admitted ED admission (parents always say when it's ambulance, treatment is instantaneous) and 'within a reasonable amount of time'?		ok
Did your child spend at least 1 night **in a hospital** in the last 6 months? **YES / NO**							

**IF NO, PLEASE SKIP 52-56 AND GO TO 57.**							
Thinking about all the times when my child stayed **in a hospital in the last 6 months**…							
52	…the diet provided suited my child's medical needs.	No issue with indicator	NA- child has not been hospitalized in the last 6 months.	NA- child has not been hospitalized in the last 6 months.			
53	…we were able to stay close to my child.	No issue with indicator					
54	…Healthcare workers took appropriate action to minimize my child's exposure to infectious diseases.	No issue with indicator					
55	…~~*Healthcare workers gave us opportunit*~~*ies to* *we were able to* bond with my child e.g., ~~*through*~~ *taking part in* his/her daily car~~*e or in acti*~~*v or* activities together.	Like, ask us to do things with our child? Clarify the example of 'through his/her daily care'. What is 'gave us opportunities' since we're not in hospital by choice, because it's a given that we have to do all the care, not 'given' the opportunity so remove that 'daily care'.					ok
56	…we were given the flexibility to decide who could be at my child's bedside in the Intensive Care Unit.	No issue with indicator					
	~~*Has your child* ***lived at home***~~ ***Have you cared for your child at home i****n the last 6 months?* **YES / NO**	Such a touchy question. I thought it's whether my child died or not. Since you are asking about being cared for at home why not just ask that? "**Have you cared for your child at home in the past 6 months?"**			Follow P10's suggestion	different meaning from before: the child could live at home but not being cared for by the parents, either because someone else did it or before the parents neglected it	ok
**IF NO, PLEASE SKIP 57 – 59.**							

	Thinking about all the times when *caring for* my child ~~lived~~ at home ***in the last 6 months, healthcare workers****...*	Sounds like my child passed on, why not make it straight forward, **'While caring for my child at home over the past 6 months'...**					ok
57	...provided sufficient consultations (*home ~~visits ph~~ysical* and/or remote *consultations*) to support the care of my child .	We didn't need. But what are 'physical consultations'? Specify home visit. And still, I think It is not applicable to parents whose child is stable at home like me.	No issues with indicator	No issues with indicator			ok
58	...gave me sufficient time to train a long-term caregiver to care for my child before discharge. ADD NA OPTION	Odd to have this question, because you're asking for the last 6 months at home, why before discharge? If he has been home for many years? It is relevant to ask for recently discharged parents only. Not under this theme, move to the ICU/hospital portion BEFORE the child even goes home.	No issues with indicator	Seems like it should come earlier on	Move to the inpatient indicators		ok
59	... did their best to help us avoid unnecessary hospitalizations for my child.	No issues with indicator	No issues with indicator	No issues with indicator			
	LENGTH		Impression: it's long. Try to reduce and eliminate the first page. Some of the items repeat behind; I see financial a few times. E.g. the reducing financial costs, had sufficient access. Medical expertise is similar to providing us knowledge, etcetc. If the first 8 indicators are	OK.			

			actually mentioned in the other indicators with HCWs are the subject with us as the receiver, than maybe they can eliminated (or vice versa).			
	OVERALL UNDERSTANDING	Health' versus 'social' care; still struggle to put organizations like club rainbow into it. Can the social care questions be separated? Then the header can be changed (the anchor text) and the desciption of who we're talking about? Then my thinking can be more organized.<br><br>Or a better term than healthcare workers to be more all-encompassing?	Ok.	OK.		

Batch 4
Italicized font and strikethrough: Suggested revisions after steering committee input

[Revised instructions]
Dear parent,

We are interested to know about your family’s experience with the health and social care system for your child’s illness. Think of all the healthcare settings and workers* who have managed your child’s illness over the past 6 months.

*Healthcare workers refer to a broad range of paid hospital, clinic, or community-based health and social care workers that your child has seen for his/her illness(es). These include doctors, nurses, physiotherapists, speech therapists, social workers, administrative personnel, pharmacists, etc.

	**Over the past 6 months, we…**	**P2 (50 mins). Category 4. 14 year old boy.**	**P5 (60 mins). Category 1. 5.5 yr old boy. <u>Cannot read English</u>.**	**Facilitator comments**	**Steering committee comments**	**Steering committee comments (cont.)**	**Steering committee comments (cont.)**
1	… had access to the ~~*a*~~ range of medical expertise needed to manage my child's condition(s).	No issue with indicator	"expertise" is difficult. My English no good.	Agree that "expertise" is not the easiest word for less educated parents, but cannot come up with something easier. Any ideas from committee?	Why not just say 'experts'	Are you suggested "had access to the range of experts needed to manage my child's condition(s)"? Excluding the word 'medical' and reverting expertise back to experts? The original indicator was 'experts'	

	… received ~~*guidance*~~ advice from healthcare workers on how to obtain my child's medical equipment(s) and supplies~~, suc~~h as *e.g.,* medications, medical consumables.	I think supplies and equipment are very different. I get equipment from HCWs but supplies from other parents and online because they're much cheaper. Can you separate them and also clarify in the statement "From HCWs"?	"guidance" is a difficult word	I do not think we should separate them. Getting outside because it is cheaper is a choice for some if they feel it is suited to their needs more so than formal channels. But, importantly, they ought to still receive guidance on how to get both supplies AND equipments via formal channels. Is "Advice" a simpler word to use than "Guidance"? Suggest to move the indicator down to change the stem to "Over the past 6 months, healthcare workers.... advised us on how to obtain XXX"	agree. I like 'advised'.		
2	… had access to sufficient financial support for my child's medical expenses so costs did not stop him/her from receiving recommended medical care.	No issue with indicator	Support from where? Hospital side?				
3	… had a care worker/team that ~~*coordinated*~~ organized my child's care across different care services.	No issue with indicator	"Arrange" easier than coordinate, I don't know that word (coordinate).	How about "Organized" as an easier term than "coordinate"?	hmm. Not sure	I also thought of "integrated" as being closer to "coordinated", but not sure if these terms are understandable by those less proficient in English. Thoughts?	
4	… received consistent information from different healthcare workers.	No issue with indicator	No issue with indicator				
5	… had access to sufficient financial support for my child's non-medical expenses, *for ~~exam~~ple e.g.,* special needs education and speech ~~the~~rapy etc so that costs did not stop him/her from receiving recommended non-medical care.	No issue with indicator	Does not understand the term 'financial support'.				

6	… received appropriate allied health support *(~~e.g., i~~*including physiotherapists, speech therapists therapists and care from other non-doctors or nurses to meet our goals for my child's development.	Hmm. Private or public? I assume this survey asks about public only.	No issue with indicator	Fair comment from P2- we did not intend to restrict this to public service providers since public + private service providers together form the health system. Do we want to clarify in definition of healthcare workers?	I don't think this matters.		
7	In the last 6 months, did your child formally receive any advice or care from a palliative or supportive care team or specialist(s)? YES / NO / I DON'T KNOW<br>*Description: *Palliative care for children is the active total care of the child's body, mind and spirit, and also involves giving support to the family.*<br>*Pediatric Palliative care is specialized medical care for people living with a serious illness. ~~Palliative care focuses on providing relief from the symptoms and stress of the illness.~~ The goal is to improve quality of life for both the child and the family.*	No issue with indicator (Child scheduled for first palliative consult next month)	No idea what is palliative care.		ok		
8	We were introduced to a palliative or supportive care team or specialist(s) at an appropriate time.	No issue with indicator but I think we need to ask whether the WAY the palliative consultant was introduced was appropriate and gentle or not. For us we were in denial for a long time and it took us many months to come to terms with it, especially spiritually. We needed our pastor, who is also a doctor, to change our mind.		Spiritual support captured by other indicators; communicating in a sensitive manner captures the 'way' the pall consultant is introduced.	if they don't know what PC or SC is then this question is a problem.	This question is only displayed to those who respond "Yes" to the prior, so they should already know PC or SC	
	**Over the past 6 months, my child's healthcare workers…**						
9	… advised us on how to obtain my child's medical equipment(s) and supplies such as medications, medical consumables.	No issue with indicator	No issue with indicator	Moved down to change the stem and shorten item.	ok		

10	…worked together to ensure my child's medical condition(s) are well managed.	No issue with indicator	No issue with indicator				
11	…were approachable when we needed advice about my child's care .	No issue with indicator	No issue with indicator				
12	…worked together towards common goals for my child’s care.	No issue with indicator	No issue with indicator				
13	…organized my child's appointments to reduce our hospital visits.	No issue with indicator	No issue with indicator				
14	…put in effort to build a trusting relationship with us.	No issue with indicator	No issue with indicator				
15	…kept us well informed about my child’s condition.	No issue with indicator	No issue with indicator				
16	…communicated with us in a sensitive *way* ~~*that was sensitive to our needs*~~*.*	No issue with indicator	What is "sensitive"? Sensitive about what. I can understand they are "understanding towards us". The English too deep.	P5 is the first parent to struggle with this so I think largely, the layman's understanding is alright.	I don't love this. What are their needs?	Agree on the term 'needs' being iffy. How about more simply "communicated with us in sensitive manner"?	
17	…gave us enough time to think about decisions for my child's care.	No issue with indicator	No issue with indicator				
18	…were responsive in managing my child's medical issues.	No issue with indicator	No issue with indicator				
19	…avoided treatments and investigations that were not aligned with our goals for my child’s care.	No issue with indicator	This means they don't anyhow do things right?				
20	…managed my child's physical symptoms to make sure he/she was comfortable.	No issue with indicator	No issue with indicator				
21	… ensured my child's wellbeing when he/she was under their care.	No issue with indicator	No issue with indicator				
22	…kept us updated about symptoms to look out for so that we knew when my child was unwell.	No issue with indicator	No issue with indicator				
23	…equipped us with skills so that we could confidently care for my child.	No issue with indicator	No issue with indicator				

24	…acknowledged ~~*and affirmed*~~ our efforts in caring for my child.	No issue with indicator	What is "Affirm"?	Perhaps just remove 'and affirm' and keep acknowledged for simplicity.	agree.		
25	…listened to us when we ~~*advocated or*~~ spoke up for my child.	It can be more specific, about managing expectations. With respect to treatments, risks and desired outcomes, on a day-to-day basis it's all about managing our expectations as parents.	What is "advocated"? I understand "Spoke up"	Suggestion from P2 is not really aligned with the intent of the question. I suspect she misunderstood the statement.	If use 'us' then why not 'our' child?	Committee raised the same concern earlier; it was the committee's agreement last time to keep to 'my child' since it is one parent respondent answering each time only. Committee to revisit this decision.	
26	…showed us care and concern.	No issue with indicator	No issue with indicator				
27	…*helped us maintain* ~~supported~~ our hopes for my child.	Every parent has different hopes. Maybe need to specify "they articulate a range of possible outcomes for my child so that I feel my hopes are supported".	No issue with indicator	P2's suggestion describes one way of supporting hopes but is not the only way. "Helped us maintain our hopes" was then trialed with P5 who had no issue. Suggest we go with "helped us maintain our hopes for my child"	Same issue. Our and 'my' seems odd to me.		
28	…prepared us for what may lie ahead.	Can you add a timeline? What may lie ahead in the next year, or 3 years, 5 years?	No issue with indicator	Timeline is probably too complicated as doctors can rarely provide such long-term preparations. Maybe just specify "prepared us for what may lie ahead for my child's condition(s)"?	I don't think the red is needed	Leave out the red.	
29	…provided us with a ~~*compassionate*~~ *kind* listening ear.	Compassion is very subjective. I think it's much more important to us that HCWs are non-judgmental so I would replace compassionate with non-judgmental.	What is "compassionate"? I understand "kind".	Suggest to use a less bombastic word; "kind' or "Non-judgmental" are both find to me, but non-judgment is harder to understand	why not 'provided a kind listening ear'. You can probasbly be kind and	True. Kindness is closer to compassion anyway.	

					judgemental<br>.		
30	…~~guided~~ *advised* us on how to reduce my child's medical expenses, for example ~~e.g.~~ access to subsidies, financing schemes.	No issue with indicator	No issue with indicator. But same struggle to understand "guide".	Suggest to replace 'guided us' with 'advised us', which is easier to understand.	ok		
31	… interacted well with my child ~~*fostered personalized interactions with*~~ *my child.*	No issue with indicator	Don't understand. But don't know how to simplify.	"Fostered" is a big word. Is "interacted with my child in a personalized way" more simple?	What is a personalized way? I don't get this. 'caring'?	What about "interacted with my child in a way that catered to his/her communicative needs[or preferences]"? Again, the word needs here- but I think it is accurate. E.g. if the child is non-verbal, he still has other communicative needs	interacted... in a personalize d way does not sound very good<br><br>I think the latter is also complicated What about interacted well with my child or something more simple like that
32	… assessed my child's physical, cognitive and emoti~~onal~~ *~~progress~~* development.	No issue with indicator	Can you use "development" rather than 'progress'. I don't understand "Progress".		ok		
33	… informed us of the range of available medical options to manage my child's condition(s).	No issue with indicator	No issue with indicator				
34	… clearly explained the advantages and disadvantages of all options for my child so that we could make informed decisions.	No issue with indicator	When words are long I cannot read. But when you read out, I understand.				

35	… discussed with us how care could be ~~*tailored*~~ *adjusted* to improve my child's comfort.	No issue with indicator	What is "Tailored"? I understand "adjusted".	OK to replace tailored with 'adjusted'	ok		
36	… involved us as much as we wanted in decision-making about my child's care.	No issue with indicator	No issue with indicator				
37	… considered our preferences for treatments given to my child.	No issue with indicator	No issue with indicator				
38	…asked us if we wanted to contribute to the community of seriously ill children ~~*e.g.*~~*, for example* letting us support other families, sharing our experience with other parents, or participating in research.	No issue with indicator	No issue with indicator				
39	…treats my child in a kind and respectful way.	No issue with indicator	No issue with indicator				
40	…were respectful of our spiritual or religious beliefs and practices.	No issue with indicator	No issue with indicator				
41	…helped us to access available parent support groups.	No issue with indicator	No issue with indicator				
42	…offered information on specialized transport for my child.	No issue with indicator	No issue with indicator				
43	…~~*addressed*~~ *supported* our family's emotional needs related to my child's condition, for example offering us counselling.	No issue with indicator	What is "addressed"? For me "support" I understand. I really don't understand addressed.	How about replacing addressed with 'supported' and to combine indicator 45 with this?	why not 'supported our family's emotional needs'	offered us couselling , if we needed it dont think we need : if we needed it	
	~~*…offered us professional emotional support e.g. counsellors, if we needed it.*~~	No issue with indicator	Same as above, no need to repeat.				
44	…helped us to find someone to take care of my child when we needed help, for example ~~e.g.~~ respite care, hospice care.	No issue with indicator	No issue with indicator				
45	…provided emotional support to my child, *for example* ~~*e.g.*~~ music therapy, counselling.	NA because they leave this to the school.	No issue with indicator	P2's response should not be NA. However, she excludes special needs school from care settings and thus put NA. This is a common perception of parents of older children (who are out of daycare).	not sure what to do	I think a minority of respondents will misuse the NA- but that is hard to avoid. We can see how this indicator	e.g > e.g is not really clear to many, say for example - this may be other places too

						performs in the pilot test. Lots of NAs will tell us we likely ought to exclude this.	
46	… helped my child access special needs school/day-care.	NA because I think the healthcare team doesn't know about the options. So they did not.	No issue with indicator	P2's response should be 'Never' rather than NA as she is describing a lack of knowledge on HCWs' part leading to a lack of service provision.	?	I think a minority of respondents will misuse the NA- but that is hard to avoid. We can see how this indicator performs in the pilot test. Lots of NAs will tell us we likely ought to exclude this.	
47	… communicated my child's medical needs in school/day-care to their staff, *for ~~exa~~mple a e.g. d*octor talked to the school about my child's feeding needs.	I don't think this is the role of HCWs. I see this as the parent's role. Don't need to ask as this is not realistic either, so not relevant.	No issue with indicator	We could decide to leave in or take out- other parents have indicated that it is very helpful when HCWs do inform the school/daycare know about the child's unique medical needs e.g. through memos. Leave it to committee.	if important then leave in.	I think best to leave in as this is the first parent to explicitly say "it's not the role of HCWs". Let's see how this indicator performs in the pilot test. Lots of NAs will tell us we likely ought to exclude this.	
48	…ensured a smooth transition of care for my child across different care sett~~ings~~ *e.g., for example* hospital to home, NICU to CICU.	No issue with indicator	No issue with indicator				

49	… attended to my child within a reasonable amount of time when we brought him/her to the Emergency Department.	No issue with indicator	No issue with indicator				
Did your child spend at least 1 night **in a hospital** in the last 6 months? **YES / NO**							
**IF NO, PLEASE SKIP 52-56 AND GO TO 57.**							
Thinking about all the times when my child stayed **in a hospital in the last 6 months**…							
50	…the diet provided suited my child's medical needs.	NA- I bring my own milk powder otherwise hospital charges me for a full tin, very expensive there.	No issue with indicator				
51	…we were able to stay close to my child.	No issue with indicator	No issue with indicator				
52	…Healthcare workers took appropriate action to minimize my child's exposure to infectious diseases.	No issue with indicator	No issue with indicator				
53	…we were able to bond with my child ~~e.g.~~, for example taking part in his/her daily care, or in activities together.	No issue with indicator	No issue with indicator				
54	…we were given the flexibility to decide who could be at my child's bedside in the Intensive Care Unit ~~*e.g.*~~, *for example* replacing caregivers.	No issue with indicator	Can put example like "Replace caregiver"?				
55	…gave me ~~*sufficient*~~ enough time to train a long-term caregiver to care for my child before discharge.	No issue with indicator	What is "Sufficient"? Can use "enough"?	Ok to replace sufficient with 'enough' for simplicity.	ok		
Has ~~**Have you cared for**~~ **your child been cared for at home** in the last 6 months? **YES / NO**							
**IF NO, PLEASE SKIP 57 – 59.**							
Thinking about all the times when ~~*caring for*~~ *my child was* ~~*at*~~ *home* ***in the last 6 months***, **healthcare workers**…							
56	…provided *enough* ~~*sufficient*~~ consultations (home visits and/or remote consultations) to support the care of my child .	No issue with indicator but maybe can add an indicator specifically for home care asking about "we are able to contact someone responsive for urgent advice when we need it."	Maybe just say Provided "enough support" (e.g. home visits and/or remote consultations) to support the care	If we replace consultations with support, support becomes repeated twice. I think OK to leave the first word as consultations since it's a specific service. Also, the ability to contact someone is captured by early indicators, just perhaps not	revision seems ok		

				specifically for the home setting. But likely no need to rehash.				
57	… did their best to help us avoid unnecessary hospitalizations for my child.	No issue with indicator						
	LENGTH	Ok.						
	OVERALL UNDERSTANDING	85% ok, except those I said were still vague and can be specified.	OK as long as you explain to me. But some words really too difficult. Can make more simple. My English not so good, my school not so high. (Max secondary level edu)	P2 does not include special needs school as part of care settings. This is a common way of thinking amongst parents of older children but less common amongst parents of younger children, forming an interested dynamic.				
