# Supplementary material for: Development of the Parental Experience with Care for Children with Serious Illnesses (PRECIOUS) quality of care measure
Source: BMC Palliat Care. 2024 Mar 8;23:66. doi: 10.1186/s12904-024-01401-x (PMC10921687; doi:10.1186/s12904-024-01401-x)
Supplement: Supplementary file 4 — Additional file 4. [file 12904_2024_1401_MOESM4_ESM.pdf]

Additional file 4. 56-item, content-validated PRECIOUS measure

***PaRental Experience with care for Children with serIOUS illnesses* (PRECIOUS).**

INSTRUCTIONS

Dear parent,

We are interested in your experience with your child’s healthcare (optional) from [Click or tap here to enter text.] **over the last 12 months**. Sharing your views will help improve the quality-of-care in the future.

While responding to the following statements, think of all the healthcare workers who have managed your child’s illness or worked with your family **in the last 12 months** (optional) at [Click or tap here to enter text.].

Healthcare workers refer to a broad range of workers that your child has seen for his/her illness(es) and/or that you have interacted with due to your child’s illness. These can include doctors, nurses, therapists, and other healthcare workers.

For each of the following statements, please choose the response option that best reflects your experience, while incorporating the perspective of other family members who care for your child.

While responding to the following statements, think of all the healthcare workers who have managed your child’s illness or worked with your family **in the last 12 months**. Healthcare workers refer to a broad range of workers in hospital, clinic, or community-based healthcare and social care settings that your child has seen for his/her illness(es) and/or that you have interacted with due to your child’s illness. These can include doctors, nurses, therapists, and other healthcare workers.

For each of the following statements, please **choose the response option that best reflects your experience.**

ITEMS & STEMS

Over the past 12 months, we…

	Always	Usually	Sometimes	Seldom	Never
1 …had access to the range of medical expertise needed to manage our child's condition(s).	O	O	O	O	O
2 …had access to sufficient financial support for our child's medical expenses so costs did not stop him/her from receiving recommended medical care.	O	O	O	O	O
3 …had a care worker/team that organized our child's care across different care services.	O	O	O	O	O
4 …received consistent information from different healthcare workers.	O	O	O	O	O
5 …had access to sufficient financial support for our child's non-medical expenses, for example special needs education and speech therapy, so	O	O	O	O	O

that costs did not stop him/her from receiving recommended non-medical care.					
6 …received appropriate allied health support including physiotherapists, speech therapists and care from other non-doctors or nurses, to meet our goals for our child's development.	O	O	O	O	O

7 In the last 12 months, did your child formally receive any advice or care from a palliative or supportive care team or specialist(s)? [DISPLAY LOGIC: IF NO, 8 NOT DISPLAYED]

**Pediatric Palliative care is specialized medical care for people living with a serious illness. The goal is to improve quality of life for both the child and the family.*

O Yes

O No

O I don't know

8 Were you introduced to a palliative or supportive care team or specialist(s) at an appropriate time?

O Too late

O Late

O Neither too late nor too early

O Early

O Too early

Over the past 12 months, our child's healthcare workersⓘ...

	Always	Usually	Sometimes	Seldom	Never
9… advised us on how to obtain our child's medical equipment(s) and supplies, such as medications, medical consumables.	O	O	O	O	O
10 ...worked together to ensure our child's medical condition(s) are well managed.	O	O	O	O	O
11 …were approachable when we needed advice about our child's care.	O	O	O	O	O
12 …worked together towards common goals for our child’s care.	O	O	O	O	O
13 …organized my child's appointments to reduce our hospital visits.	O	O	O	O	O
14 …put in effort to build a trusting relationship with us.	O	O	O	O	O

15 …kept us well informed about our child’s condition.	O	O	O	O	O
16 …communicated with us in a sensitive way.	O	O	O	O	O
17 …gave us enough time to think about decisions for our child's care.	O	O	O	O	O
18 …were responsive in managing our child's medical issues.	O	O	O	O	O
19 …avoided treatments and investigations that were not aligned with our goals for our child’s care.	O	O	O	O	O
20 …managed our child's physical symptoms to make sure he/she was comfortable.	O	O	O	O	O
21 …ensured our child's wellbeing when he/she was under their care.	O	O	O	O	O
22 …kept us updated about symptoms to look out for so that we knew when our child was unwell.	O	O	O	O	O
23 …equipped us with skills so that we could confidently care for our child.	O	O	O	O	O
24 …acknowledged our efforts in caring for our child.	O	O	O	O	O
25 …listened to us when we spoke up for our child.	O	O	O	O	O
26 …showed us care and concern.	O	O	O	O	O
27 …helped us maintain our hopes for our child.	O	O	O	O	O
28 …prepared us for what may lie ahead.	O	O	O	O	O
29 …provided us with a kind listening ear.	O	O	O	O	O
30 …advised us on how to reduce our child's medical expenses e.g. access to subsidies, financing schemes.	O	O	O	O	O
31 … interacted well with our child.	O	O	O	O	O
32 ...assessed our child's physical, cognitive and emotional development.	O	O	O	O	O
33 … informed us of the range of available medical options to manage our child’s condition(s).	O	O	O	O	O
34 … clearly explained the advantages and disadvantages of all options for our child so that we could make informed decisions.	O	O	O	O	O

35 … discussed with us how care could be adjusted to improve our child's comfort.	O	O	O	O	O
36 … involved us as much as we wanted in decision-making about our child's care.	O	O	O	O	O
37 … considered our preferences for treatments given to our child.	O	O	O	O	O
38 …asked us if we wanted to contribute to the community of seriously ill children, for example letting us support other families, sharing our experience with other parents, or participating in research.	O	O	O	O	O
39 …treated our child in a kind and respectful way.	O	O	O	O	O

Over the past 12 months, our child's healthcare workersⓘ...

	Always	Usually	Sometimes	Seldom	Never	NA
40 …were respectful of our spiritual or religious beliefs and practices.	O	O	O	O	O	O
41 …helped us to access available parent support groups.	O	O	O	O	O	O
42 …offered information on specialized transport for our child.	O	O	O	O	O	O
43 …supported our family's emotional needs related to our child's condition, for example offering us counselling.	O	O	O	O	O	O
44 …helped us to find someone to take care of our child when we needed help, for example respite care, hospice care.	O	O	O	O	O	O
45 …provided emotional support to our child, for example music therapy, counselling.	O	O	O	O	O	O
46 … helped our child access special needs school/day-care.	O	O	O	O	O	O
47 ...communicated our child's medical needs in school/day-care to their staff, for example a doctor talked to the school about our child's feeding needs.	O	O	O	O	O	O
48 …ensured a smooth transition of care for our child across different care settings, for example hospital to home, NICU to CICU.	O	O	O	O	O	O
49 … attended to our child within a reasonable amount of time when we brought him/her to the Emergency Department.	O	O	O	O	O	O

Did your child spend at least 1 night in a hospital in the last 12 months? [DISPLAY LOGIC: IF NO, 50-55 NOT DISPLAYED].

O No

O Yes

Thinking about all the times when our child stayed in a hospital in the last 12 months…

	Always	Usually	Sometimes	Seldom	Never	NA
50 …the diet provided suited our child's medical needs.	O	O	O	O	O	O
51 …we were able to stay close to our child.	O	O	O	O	O	O
52 …Healthcare workers took appropriate action to minimize our child's exposure to infectious diseases.	O	O	O	O	O	O
53 …we were able to bond with our child, for example taking part in his/her daily care, or in activities together.	O	O	O	O	O	O
54 …we were given the flexibility to decide who could be at our child's bedside in the Intensive Care Unit, for example replacing caregivers.	O	O	O	O	O	O
55 …gave us enough time to train a long-term caregiver to care for our child before discharge.	O	O	O	O	O	O

Has your child been cared for at home in the last 12 months? [DISPLAY LOGIC: IF NO, 56-57 NOT DISPLAYED].

O No

O Yes

Thinking about all the times when our child was home in the last 12 months, healthcare workers…

	Always	Usually	Sometimes	Seldom	Never	NA
56 ...provided enough consultations (home visits and/or remote consultations) to support the care of our child.	O	O	O	O	O	O
57 … did their best to help us avoid unnecessary hospitalizations for our child.	O	O	O	O	O	O
