# Supplementary material for: Development of the Parental Experience with Care for Children with Serious Illnesses (PRECIOUS) quality of care measure
Source: BMC Palliat Care. 2024 Mar 8;23:66. doi: 10.1186/s12904-024-01401-x (PMC10921687; doi:10.1186/s12904-024-01401-x)
Supplement: Supplementary file 5 — Additional file 5. [file 12904_2024_1401_MOESM5_ESM.pdf]

Additional file 5 - PRECIOUS Pilot-testing results at item-level											
n	*Indicator*	*N valid*	*% floor (never)*	*25% percentile*	*Mean (SD)*	*50% percentile*	*75% percentile*	*% ceiling (Always)*	*Min*	*Max*	*Skew*
p1	…had access to the range of medical expertise needed to manage our child's condition(s).	30	0	2	2.67 (0.99)	3	3	20	1	4	-0.36
p2	…had access to sufficient financial support for our child's medical expenses so costs did not stop him/her from receiving recommended medical care.	30	6.7	1	2.23 (1.10)	3	3	6.7	0	4	-0.47
p3	…had a care worker/team that organized our child's care across different care services.	30	20	1	1.87 (1.31)	2	3	10	0	4	-0.03
p4	…received consistent information from different healthcare workers.	30	3.3	2	2.4 (0.97)	2.5	3	10	0	4	-0.41
p5	…had access to sufficient financial support for our child's non-medical expenses, such as special needs education or speech therapy, so that costs did not stop him/her from receiving recommended non-medical care.	30	13.3	1	1.7 (1.09)	2	3	3.3	0	4	0.13
p6	…received appropriate allied health support including physiotherapists, speech therapists and care from other non-doctors or nurses, to meet our goals for our child's development.	30	3.3	2	2.20 (0.92)	2	3	6.7	0	4	-0.14
			Yes	*No*	*I Don't Know*						
p7	In the last 12 months, did your child formally receive any advice or care from a palliative* or supportive care team or specialist(s)?<br>*Pediatric Palliative care is specialized medical care for children living with a serious illness. The goal is to improve quality of life for both the child and the family.	30	8	20	2						
			% Too late (0)	*% Late (1)*	*% Neither too late nor too early (2)*	*% Early (3)*	*% Too early (4)*				
p8	We were introduced to a palliative or supportive care team or specialist(s) at an appropriate time.	8	0	12.5	75	12.5	0				
p9	… advised us on how to obtain our child's medical equipment(s) and supplies, such as medications or medical consumables.	30	0	2	2.63 (0.81)	3	3	13.3	1	4	-0.04

p10	...worked together to ensure our child's medical condition(s) are well managed.	30	3.3	2	2.77 (0.97)	3	3	20	0	4	-0.90
p11	...were approachable when we needed advice about our child's care.	30	0	3	2.93 (0.74)	3	3	20	1	4	-0.42
p12	...worked together towards common goals for our child’s care.	30	0	1	2.93 (0.83)	3	3	20	1	4	-0.99
p13	...organized my child's appointments to reduce our hospital visits.	30	13.3	1	2.13 (1.28)	2	3	16.7	0	4	-0.15
p14	...put in effort to build a trusting relationship with us.	30	3.3	2	2.57 (1.14)	3	3	23	0	4	-0.38
p15	...kept us well informed about our child’s condition.	30	3.3	2	2.6 (1.04)	3	3	20	0	4	-0.46
p16	...communicated with us in a sensitive way.	30	6.7	2	2.73 (1.14)	3	4	26.7	0	4	-0.87
p17	...gave us enough time to think about decisions for our child's care.	30	0	3	3.03 (0.72)	3	4	26.7	2	4	-0.05
p18	...were responsive in managing our child's medical issues.	30	0	2	2.8 (0.85)	3	3	23.3	1	4	0.04
p19	...avoided treatments and investigations that were not aligned with our goals for our child’s care.	30	0	2	2.87 (0.82)	3	3	20	1	4	-0.52
p20	...managed our child's physical symptoms to make sure he/she was comfortable.	30	0	2	2.87 (0.73)	3	3	20	2	4	0.20
p21	...ensured our child's wellbeing when he/she was under their care.	30	0	2	2.93 (0.78)	3	4	26.7	2	4	0.11
p22	...kept us updated about symptoms to look out for so that we knew when our child was unwell.	30	6.7	2	2.63 (1.10)	3	3	23.3	0	4	-0.66
p23	...equipped us with skills so that we could confidently care for our child.	30	3.3	2	2.63 (0.93)	3	3	16.7	0	4	-0.53
p24	...acknowledged our efforts in caring for our child.	30	0	2	2.9 (1.44)	3	4	30	2	4	0.19
p25	...listened to us when we spoke up for our child.	30	3.3	2	2.83 (1.02)	3	4	30	0	4	-0.65
p26	...showed us care and concern.	30	3.3	2	2.8 (1.03)	3	4	30	0	4	-0.55
p27	...helped us maintain our hopes for our child.	30	3.3	2	2.47 (1.04)	3	4	20	0	4	-0.10
p28	...prepared us for what may lie ahead.	30	6.7	2	2.43 (1.19)	2	4	26.7	0	4	-0.15
p29	...provided us with a kind listening ear.	30	16.7	2	2.7 (1.12)	3	4	33.3	1	4	-0.14
p30	...advised us on how to reduce our child's medical expenses, such as access to subsidies or financing schemes.	30	10	1	1.7 (1.09)	1.5	2	6.7	0	4	0.45
p31	... interacted well with our child.	30	0	2	2.6 (1.00)	3	3	20	1	4	-0.17
p32	...assessed our child's physical, cognitive and emotional development.	30	3.3	2	2.4 (0.89)	2	3	10	0	4	-0.28
p33	... informed us of the range of available medical options to manage our child’s condition(s).	30	0	2	2.43 (0.94)	2	3	13.3	1	4	0.07

p34	… clearly explained the advantages and disadvantages of all options for our child so that we could make informed decisions.	30	3.3	2	2.63 (1.0)	3	3	20	0	4	-0.48
p35	… discussed with us how care could be adjusted to improve our child's comfort.	30	0	1	2.4 (1.16)	2	3	23.3	1	4	0.11
p36	… involved us as much as we wanted in decision-making about our child's care.	30	0	2	2.87 (0.94)	3	4	26.7	1	4	-0.50
p37	… considered our preferences for treatments given to our child.	30	0	2	2.83 (0.91)	3	4	26.7	1	4	-0.22
p38	…asked us if we wanted to contribute to the community of seriously ill children, such as letting us support other families or participating in research.	30	20	1	1.73 (1.31)	2	3	13.3	0	4	0.32
p39	…treated our child in a kind and respectful way.	30	3.3	3	3.03 (0.89)	3	4	26.7	0	4	-1.56
p40	…were respectful of our spiritual or religious beliefs and practices.	23	0	2	2.91 (0.79)	3	3	21.7	1	4	-0.41
p41	…helped us to access available parent support groups.	26	7.7	1	1.81 (1.2)	3	4	11.5	0	4	0.52
p42	…offered information on specialized transport for our child.	23	34.8	0	1.3 (1.3)	1	2	4.3	0	4	0.52
p43	…supported our family's emotional needs related to our child's condition, for example offering us counselling.	29	13.8	1	1.76 (1.24)	3	4	10.3	0	4	0.35
p44	…helped us to find someone to take care of our child when we needed help, such as respite care or hospice care.	20	30	0	1.5 (1.40)	1	3	10	0	4	0.48
p45	…provided emotional support to our child, such as music therapy or counselling.	24	33.3	0	1.08 (1.1)	1	1.5	4.17	0	4	1.04
p46	… helped our child access special needs school/day-care.	25	32	0	1.68 (1.46)	2	3	12	0	4	0.17
p47	...communicated our child's medical needs in school/day-care to their staff, such as talking to the school about our child's feeding needs.	22	22.7	1	1.77 (1.30)	2	3	9.1	0	4	0.01
p48	…ensured a smooth transition of care for our child across different care settings, such as hospital to home, NICU to CICU.	19	5.3	2	2.58 (0.90)	3	3	10.5	0	4	-0.94
p49	… attended to our child within a reasonable amount of time when we brought him/her to the Emergency Department.	26	7.7	2	2.35 (1.06)	2	3	11.5	0	4	-0.52
	Did your child spend at least 1 night in a hospital in the last 12 months?	22									
p50	…the diet provided suited our child's medical needs.	17	11.8	2	2.35 (1.17)	3	3	11.8	0	4	-0.72

p51	…we were able to stay close to our child.	22	9.1	2	3 (0.98)	3	4	36.4	1	4	-0.63
p52	…Healthcare workers took appropriate action to minimize our child's exposure to infectious diseases.	22	4.5	2	2.82 (1.05)	3	4	27.3	0	4	-0.88
p53	…we were able to bond with our child, such as taking part in his/her daily care, or in activities together.	22	0	2	3.05 (0.90)	3	4	36.4	1	4	-0.49
p54	…we were given the flexibility to decide who could be at our child's bedside in the Intensive Care Unit, such as replacing caregivers.	20	0	3	3.05 (0.69)	3	3.5	25	2	4	-0.06
p55	…gave us enough time to train a long-term caregiver to care for our child before discharge.	13	7.7	2	2.69 (1.11)	3	3	23.1	0	4	-0.89
	Has your child been cared for at home in the last 12 months?	16									
p56	...provided enough consultations (home visits and/or remote consultations) to support the care of our child.	13	15.4	1	2.31 (1.49)	2	4	30.8	0	4	-0.24
p57	… did their best to help us avoid unnecessary hospitalizations for our child.	14	0	2	2.79 (1.19)	3	4	35.7	1	4	-0.43
0 – never ; 1 – seldom ; 2 – sometimes ; 3 – often ; 4 - always											
