# Supplementary material for: Development of the Parental Experience with Care for Children with Serious Illnesses (PRECIOUS) quality of care measure
Source: BMC Palliat Care. 2024 Mar 8;23:66. doi: 10.1186/s12904-024-01401-x (PMC10921687; doi:10.1186/s12904-024-01401-x)
Supplement: Supplementary file 6 — Additional file 6: eFigure 1. [file 12904_2024_1401_MOESM6_ESM.pdf]

DukeNUS Medical School | Lien Centre for Palliative Care

Additional File eFigure 1. Infographic of selected results from Pilot-Testing PRECIOUS

CARING FOR SERIOUSLY ILL CHILDREN IN SG

Our Quality-of-care

Dear team and parents, here we share selected findings from the Pilot-study (n = 30) of the PaRental Experience with care for Children with serIOUS illnesses (PRECIOUS) project.

Quality of Children's Palliative Care, Widger et al.

OVERALL QUALITY-OF-CARE

On a scale of 0 (Poor) to 4 (Excellent), parents rated Singapore's overall quality-of-care for seriously ill children 2.3 / 4.0, **suggesting there is room for improvement.**

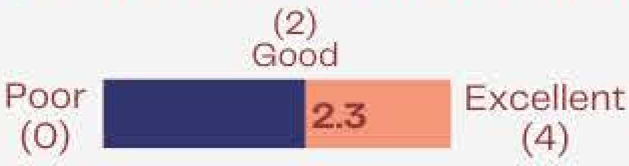

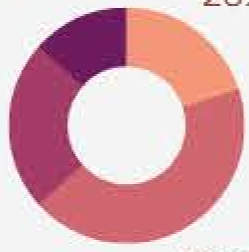

SUPPORT FOR SIBLINGS

Around half of Singapore's seriously ill children have sibling(s)...

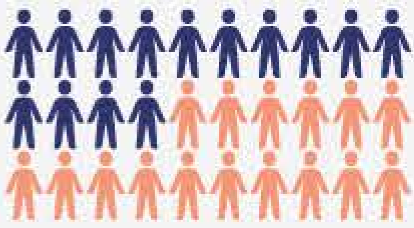

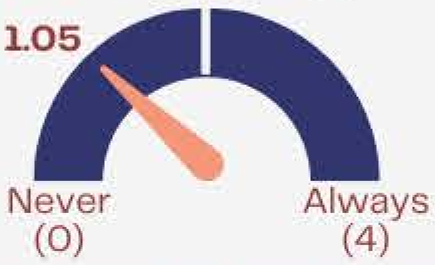

...but Sibling Support is Rarely experienced, **suggesting a need to introduce formal sibling support.**

Measure of Processes of Care, Rosenbaum et al.

MEETING PARENTAL NEEDS

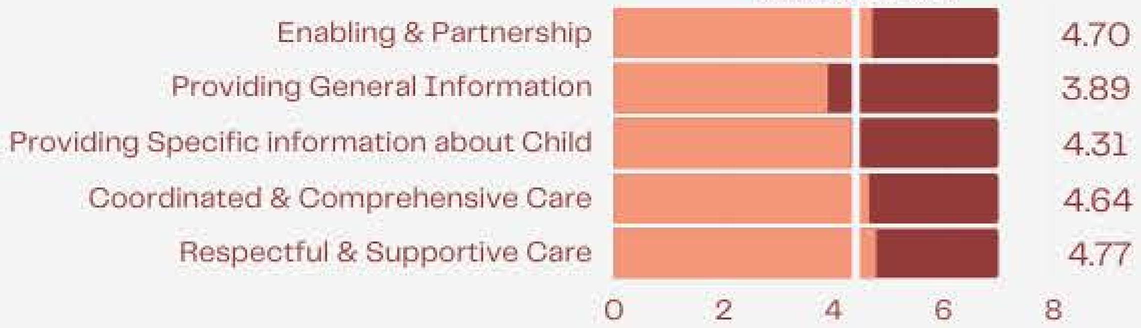

Parental needs are only "sometimes" being met on all scales, **suggesting existing gaps in the responsiveness of current care delivery across all domains.**

PaRental Experience with care for Children with serIOUS illnesses, Ang et al.

SELECTED INDICATORS

"Access to sufficient financial support for child's non-medical expenses (such as therapy), so that costs did not stop our child from receiving recommended non-medical care"

Most parents "Seldom" experience this. **Suggesting re-evaluation of financial support schemes may be needed.**

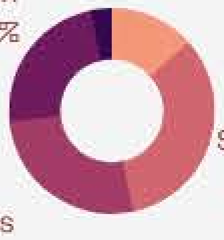

"Providing emotional support to our child"

80% report that it is applicable to their child, yet most never experience any, **suggesting a need to introduce formal child-patient support**

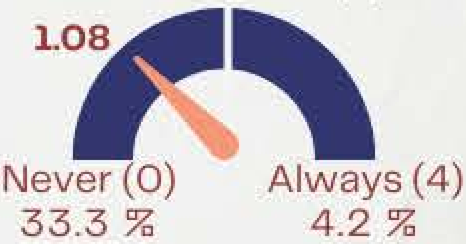

"Treating our child in a kind & respectful way"

Well-performing on average, **suggesting healthcare workers treat child-patients with dignity**

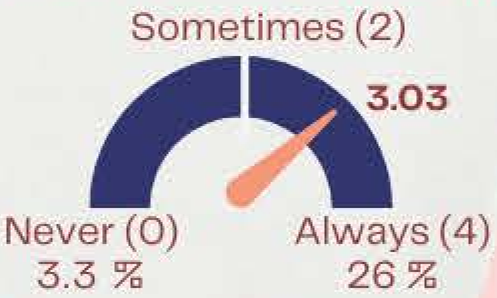

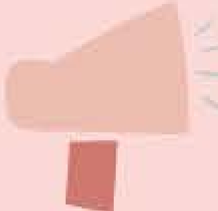

What's next?

1. Confirm these findings in a larger sample of parents
2. Identify, develop & test interventions targeting gaps in care

Your voice matters.
